# Supplementary material for: N-Arylation of 3-Formylquinolin-2(1H)-ones Using Copper(II)-Catalyzed Chan–Lam Coupling
Source: Molecules. 2022 Nov 30;27(23):8345. doi: 10.3390/molecules27238345 (PMC9735505; doi:10.3390/molecules27238345)

Supporting Information

Table of contents

1-Compounds characterization (**6a**, **7a**) 1

2-X-Ray Analytical Data for Compound **9a**........ 1

3-Spectral Characterization of Compounds........ 3

4-Unsuccessful reactions 47

1-Compounds characterization (6a-7a)

2-chloroquinoline-3-carbaldehyde : pale yellow solid. ^{1}H NMR (200 MHz, $CDCl_3$) δ 10.56 (s, 1H), 8.75 (s, 1H), 8.14 – 7.80 (m, 3H), 7.72 – 7.58 (m, 1H).

2-oxo-1,2-dihydroquinoline-3-carbaldehyde: pale yellow solid. ^{1}H NMR (200 MHz, DMSO) δ 12.21 (s, 1H), 10.24 (s, 1H), 8.50 (s, 1H), 7.91 (dd, *J* = 7.9, 1.5 Hz, 1H), 7.66 (ddd, *J* = 8.5, 7.1, 1.5 Hz, 1H), 7.36 (d, *J* = 8.3 Hz, 1H), 7.25 (ddd, *J* = 8.2, 7.1, 1.1 Hz, 1H).

2-X-Ray Analytical Data for Compound 9a

Crystal structure determination

The crystal structure of 9b was determined by X-ray diffraction at 293 K. Data collection was done on a SMART-APEX II CCD diffractometer system. Data was reduced using SAINTPLUS [1]. Empirical absorption corrections were applied using SADABS [2]. The structure was solved by direct methods, completed by Difference Fourier Synthesis, and refined by least-squares using SHELXL [3,4]. All atoms were anisotropically refined, the hydrogen atoms positions were calculated after each cycle of refinement with SHELXL using a riding model for each structure, with C–H distance of 0.93 or 0.96 Å. Uiso(H) values were set equal to 1.2 Ueq of the parent carbon atom. The crystal of 9b was refined giving a R-factor of 17.77% due to poor crystal quality. However, the composition and molecular formula was corroborated using 1H-NMR, 13C-NMR, and High Resolution Mass Spectrometry (HRMS). Additional crystallographic and refinement details are given in Table S1. Structural drawing (Fig. S1) was carried out with DIAMOND-3.2k, supplied by Crystal Impact [5]. Crystallographic data for the structure reported in this paper have been deposited with the Cambridge Crystallographic Data Centre as supplementary publication number CCDC-2205662.

References

[1] M. SAINTPLUS, V6.22, Bruker AXS Inc., Madison,Wisconsin, USA

[2] W. SADABS, V2.05,BrukerAXSInc.,Madison,Wisconsin,USA

[3] G. M. Sheldrick,Acta Crystallogr., Sect. C: Struct. Chem.,2015,71,3–8

[4] G. M. Sheldrick, Acta Crystallogr. Sect. A Found. Adv., 2015, 71, 3–8.

[5] K. Brandenburg, DIAMOND, Version 3.2k, Crystal Impact GbR, Bonn, Germany, 2014.

Table S1. Crystal data and structure refinement for compound **9b**.

CCDC	2205662
Empirical formula	$C_{17}H_{13}NO_2$
Formula weight	263.28
Temperature/K	293(2)
Crystal system	Monoclinic
Space group	*P2₁/c*
a/Å	5.3826(7)
b/Å	23.451(3)
c/Å	10.7551(16)
α/°	90.00
β/°	102.248(14)
γ/°	90.00
Volume/Å^3	1326.7(3)
Z	4
ρ_{calc}mg/mm^3	1.318
m/mm^{-1}	0.087
F(000)	552.0
Crystal size/mm^3	0.092 × 0.083 × 0.052
2Θ range for data collection	5.204 to 51.99°
Index ranges	-6 ≤ h ≤ 6, -28 ≤ k ≤ 28, -13 ≤ l ≤ 12
Reflections collected	16220
Independent reflections	2612[R_{int} = 0.0698, R_{sigma} = 0.0419]
Data/restraints/parameters	2612/6/182
Goodness-of-fit on F^2	1.499
Final R indexes [I>=2σ (I)]	R_1 = 0.1777, wR_2 = 0.2472
Final R indexes [all data]	R_1 = 0.1891, wR_2 = 0.2512
Largest diff. peak/hole / e Å^{-3}	0.26/-0.42

3-Spectral Characterization of Compounds

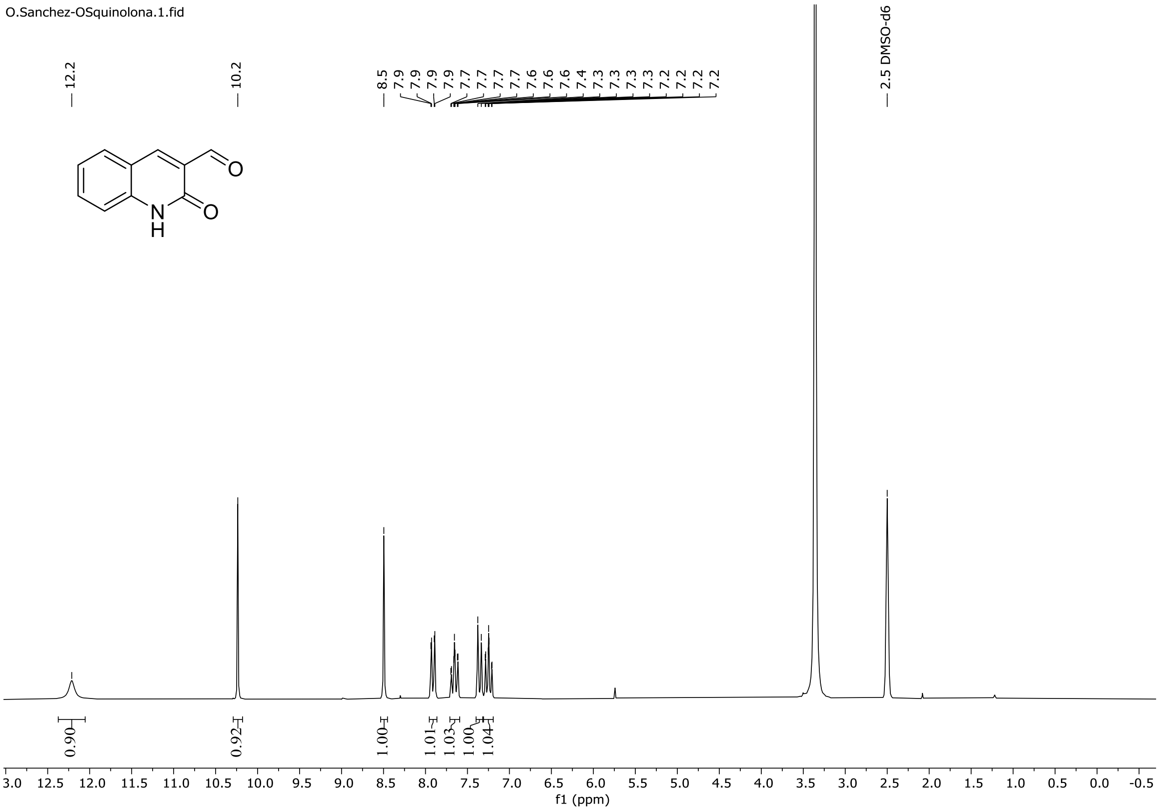

Figure S1. ^{1}H-NMR spectrum of **7a** (DMSO, 200 MHz)

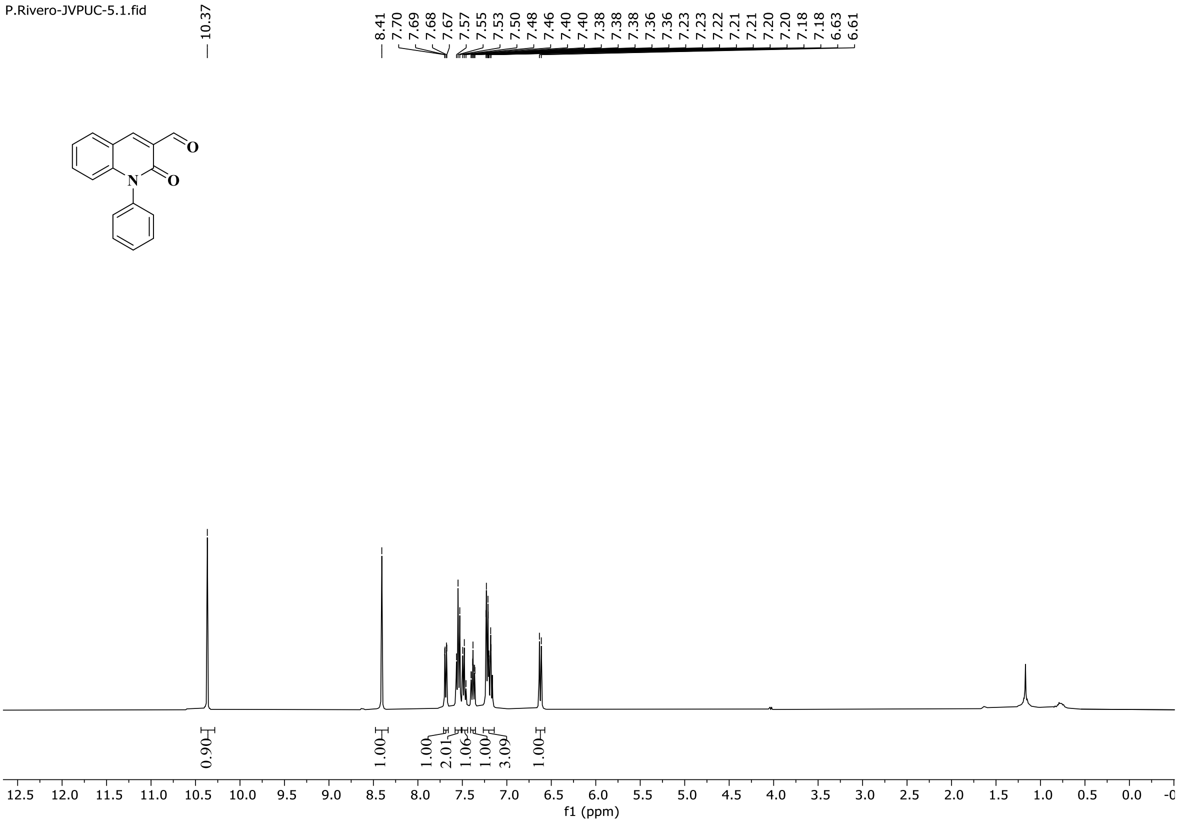

Figure S2. ^{1}H-NMR spectrum of **9b** ($CDCl_3$, 400 MHz)

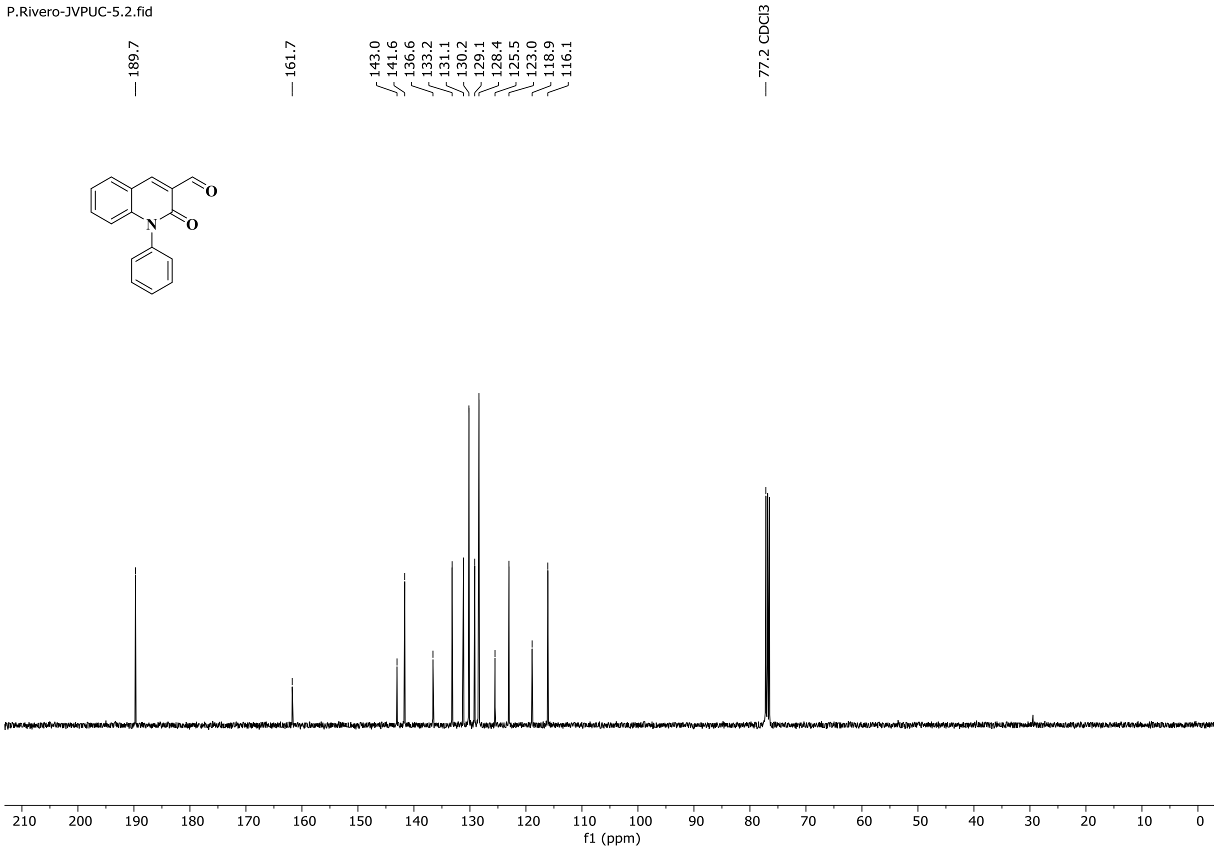

Figure S3. ^{13}C-NMR spectrum of **9b**($CDCl_3$, 101 MHz)

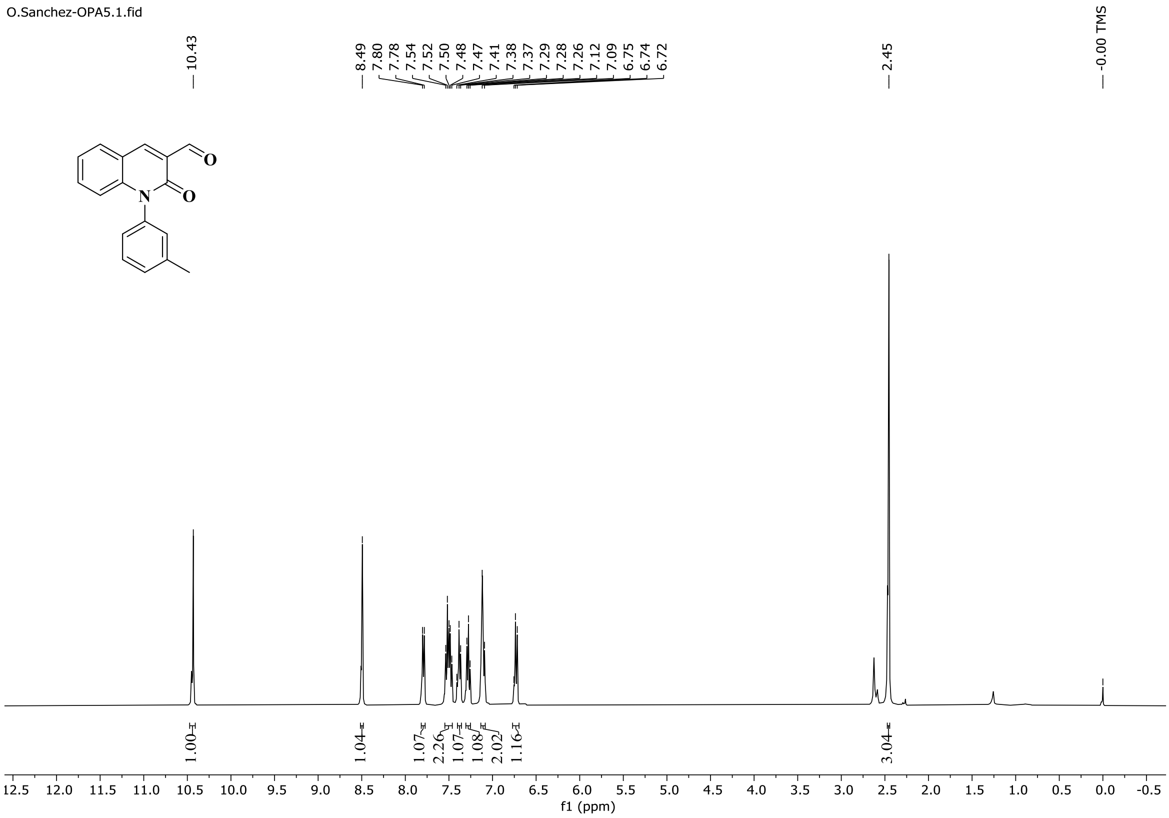

Figure S4. ^{1}H-NMR spectrum of **9c** ($CDCl_3$, 400 MHz)

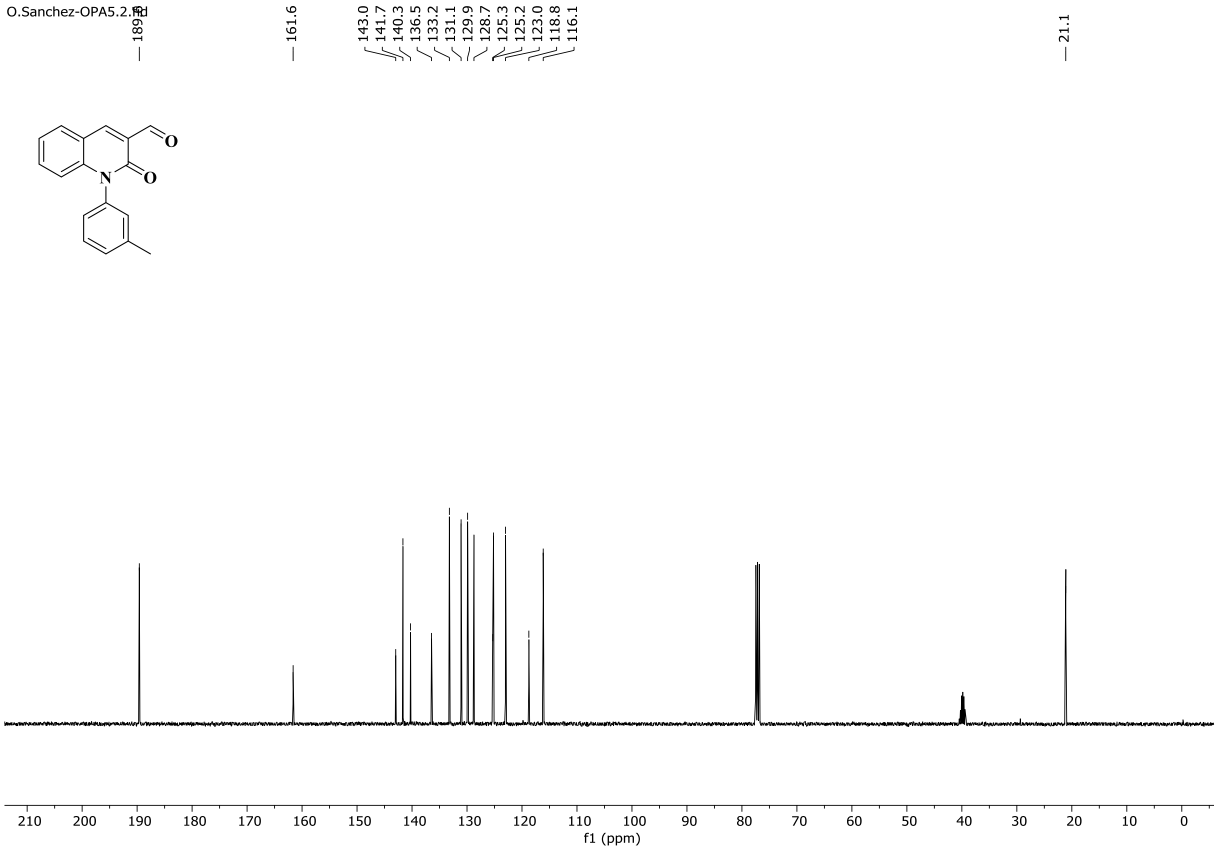

Figure S5. ^{13}C-NMR spectrum of **9c** ($CDCl_3$, 101 MHz)

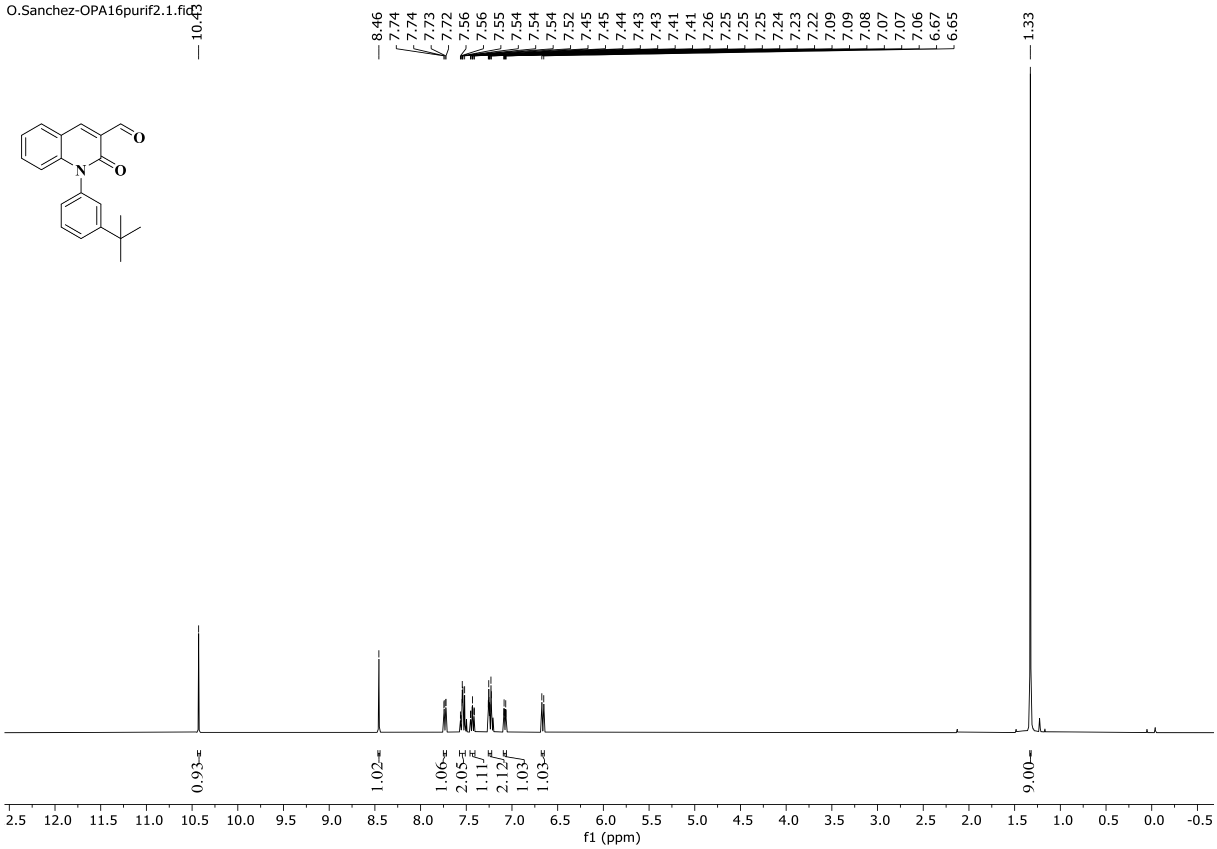

Figure S6. ^{1}H-NMR spectrum of **9d** ($CDCl_3$, 400 MHz)

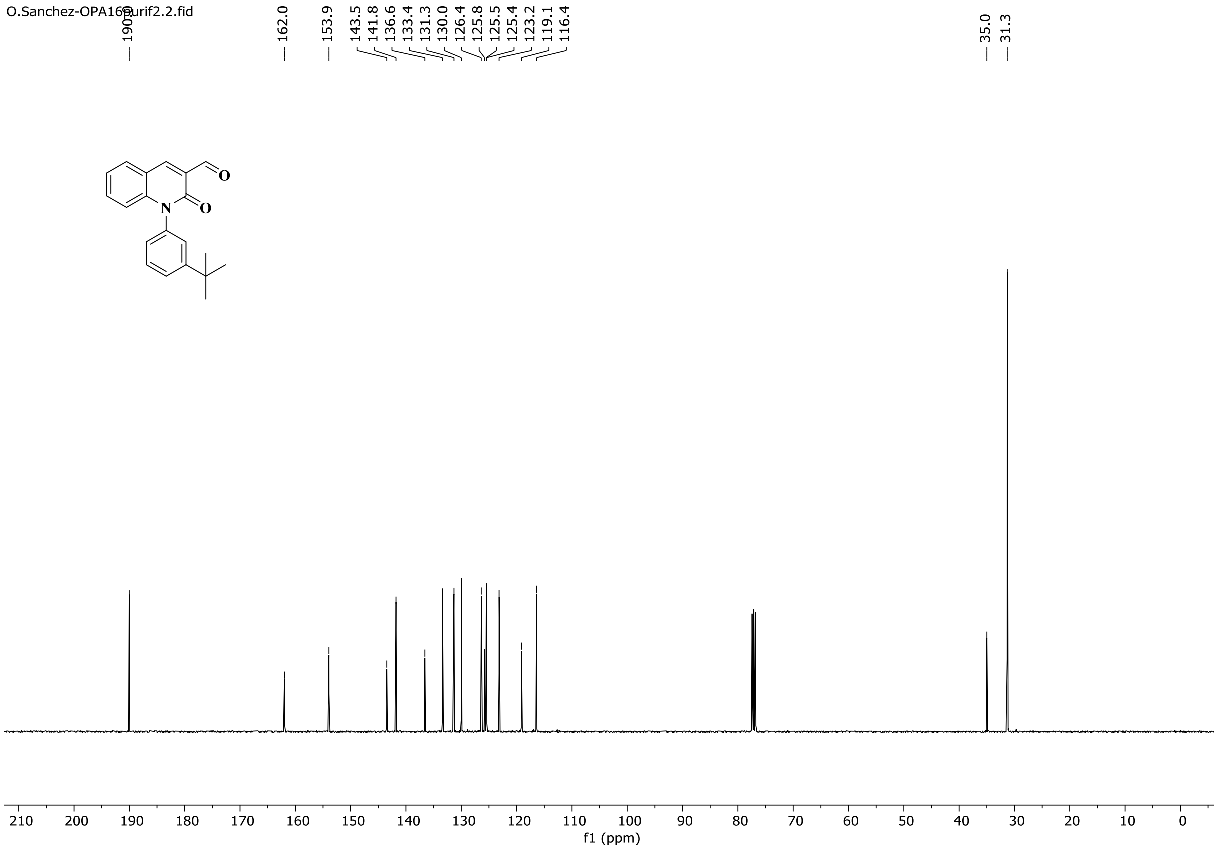

Figure S7. ^{13}C-NMR spectrum of **9d** ($CDCl_3$, 101 MHz)

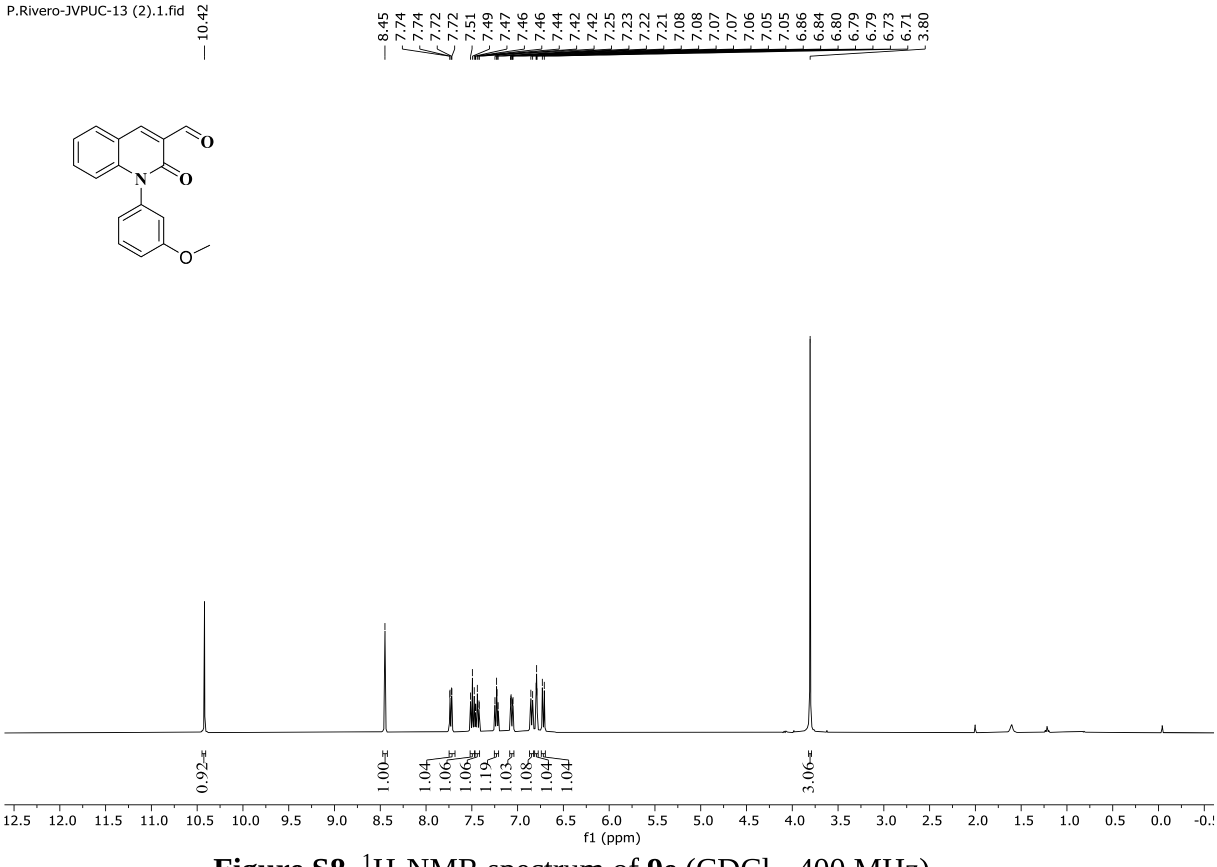

Figure S8. ^{1}H-NMR spectrum of **9e** ($CDCl_3$, 400 MHz)

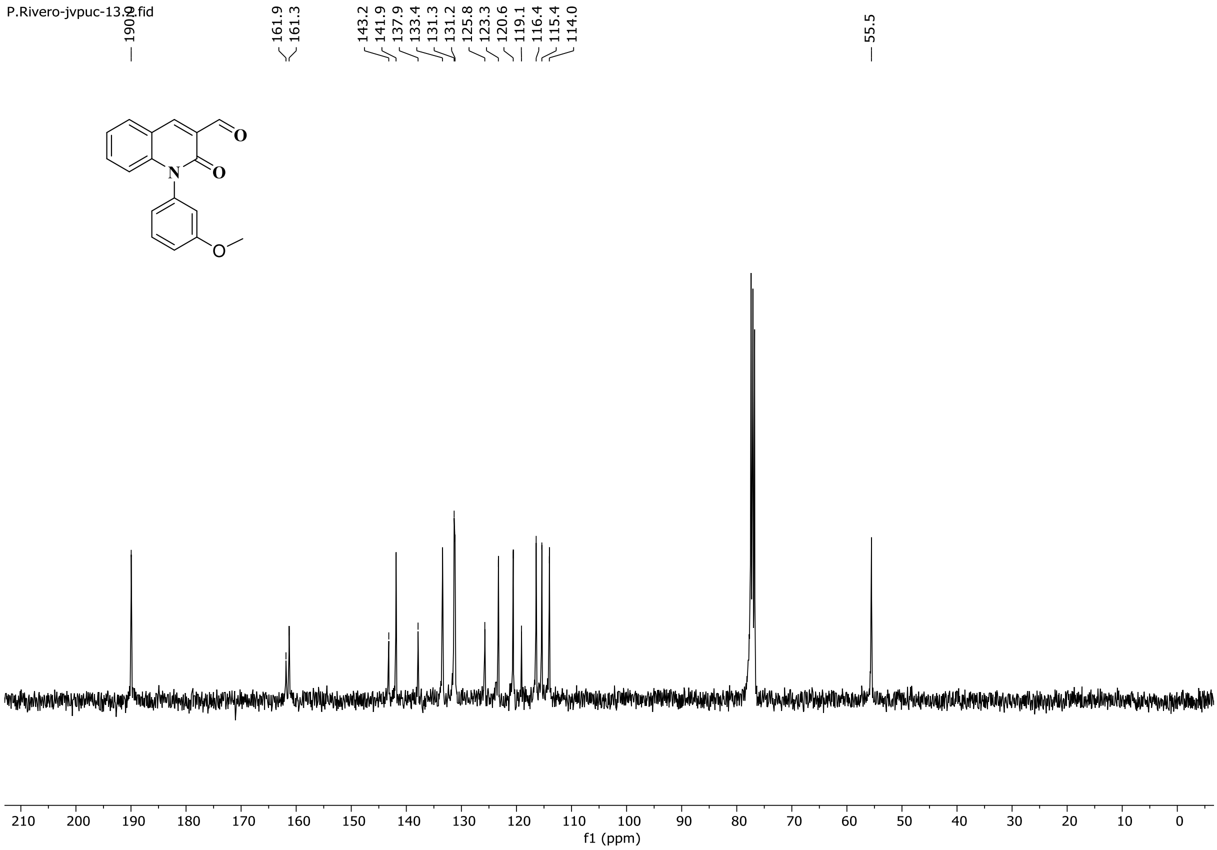

Figure S9. ^{13}C-NMR spectrum of **9e** ($CDCl_3$, 101 MHz)

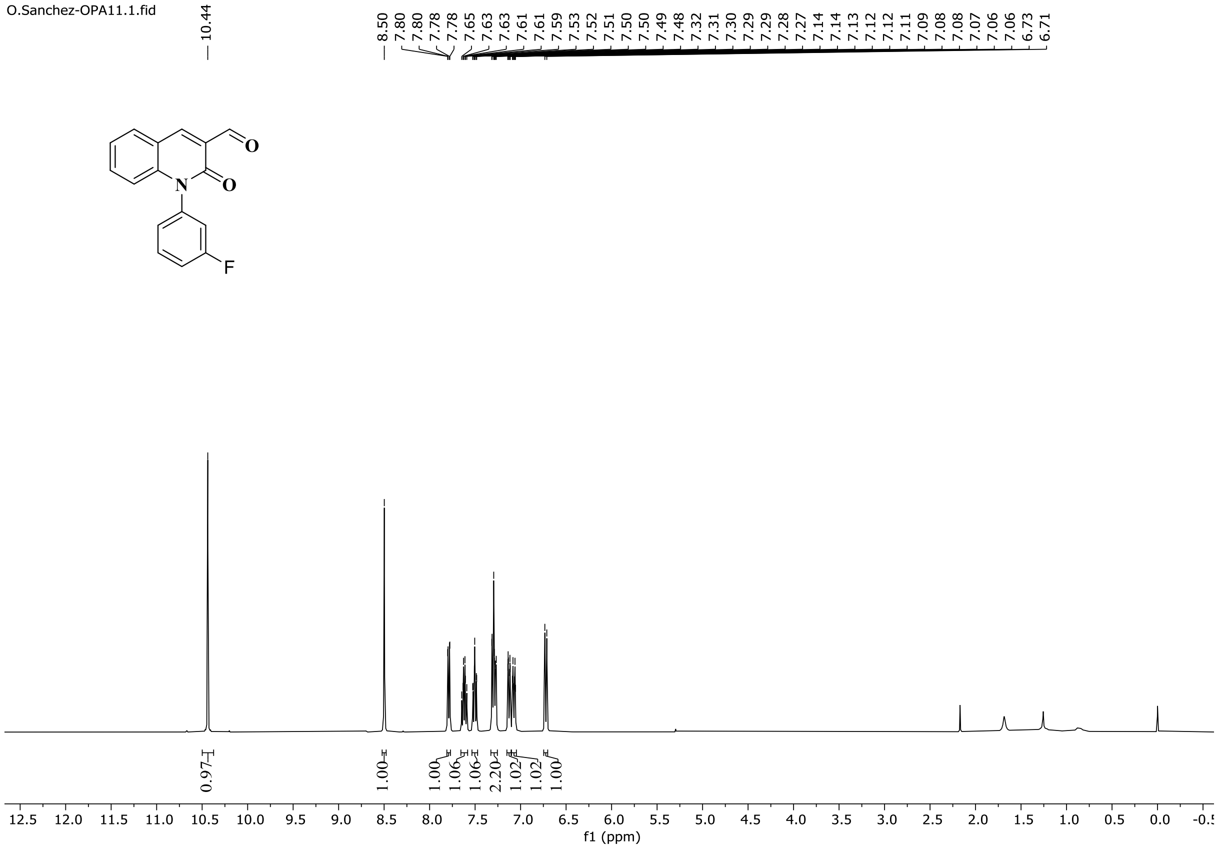

Figure S10. ^{1}H-NMR spectrum of **9f** ($CDCl_3$, 400 MHz)

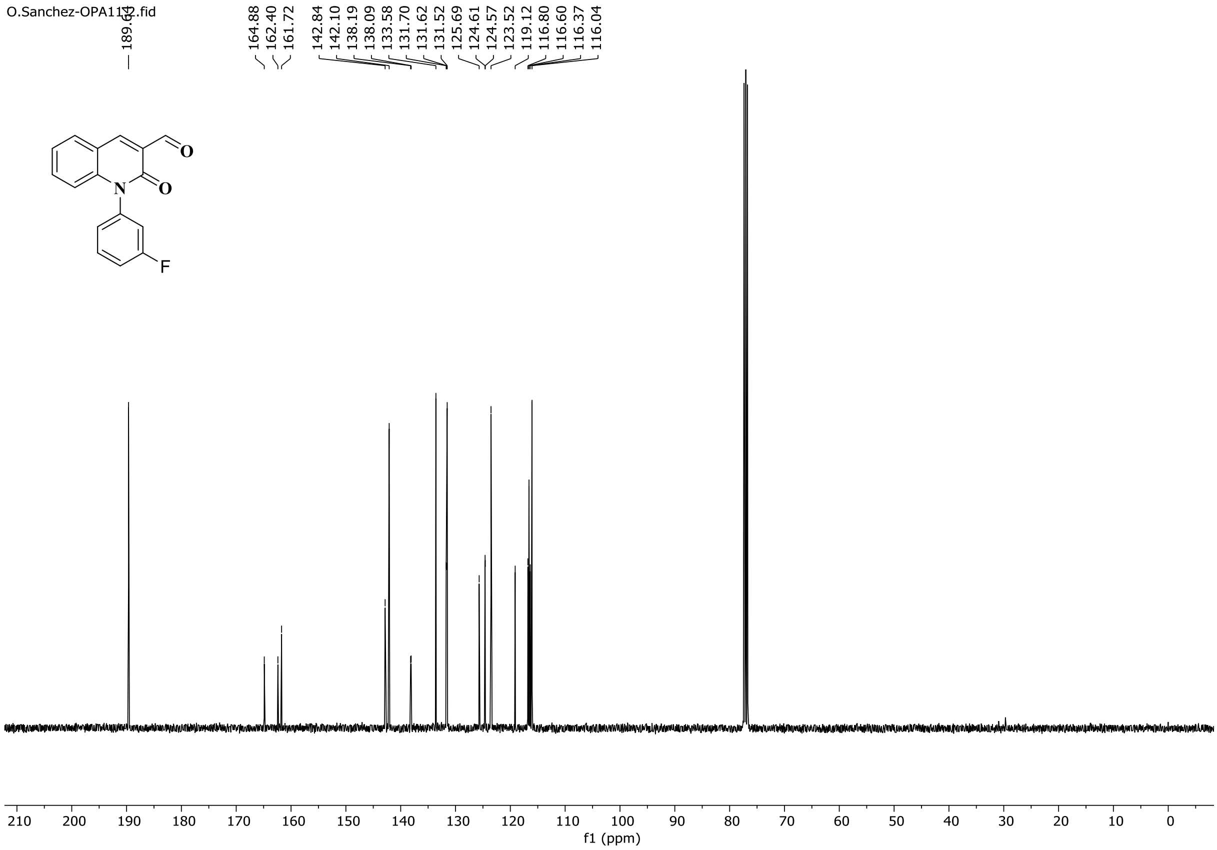

Figure S11. ^{13}C-NMR spectrum of **9f** ($CDCl_3$, 101 MHz)

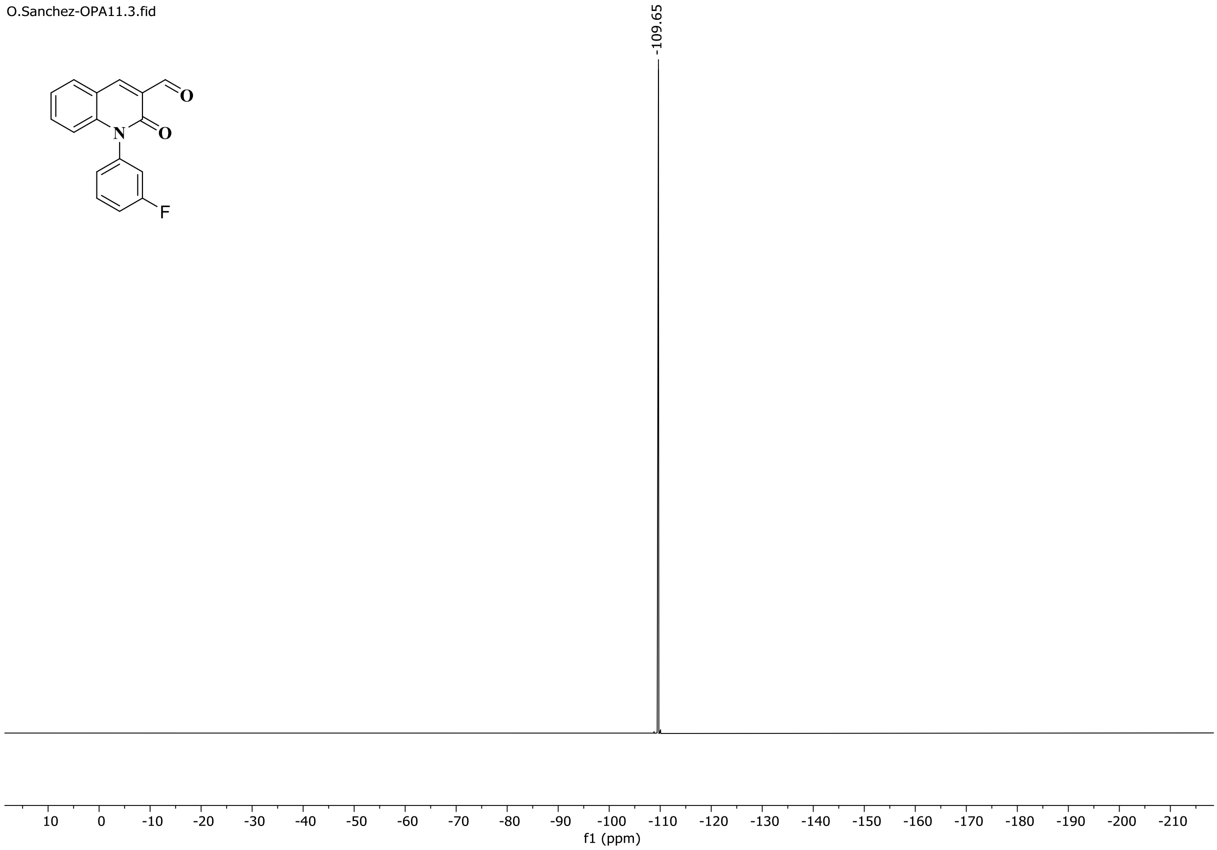

Figure S12. ^{19}F-NMR spectrum of **9f**($CDCl_3$, 376 MHz)

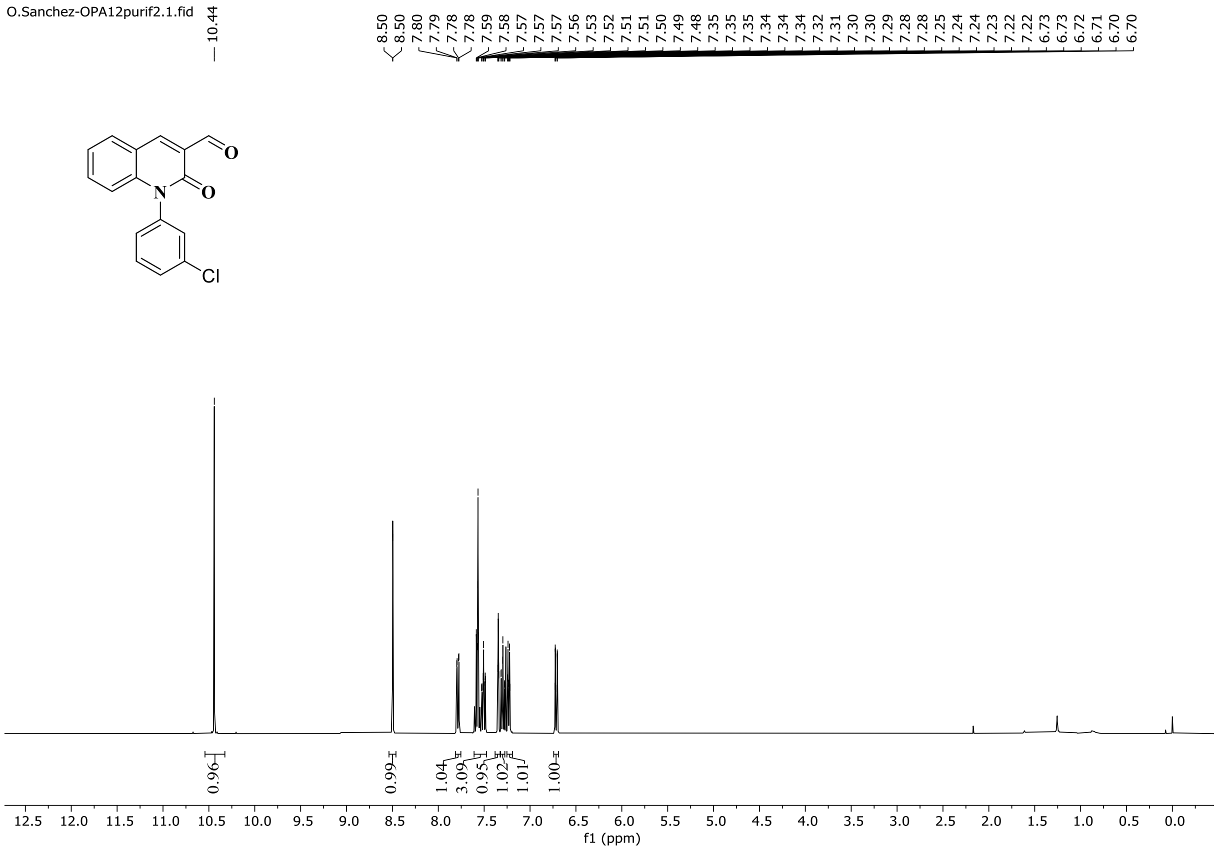

Figure S13. ^{1}H-NMR spectrum of **9g** ($CDCl_3$, 400 MHz)

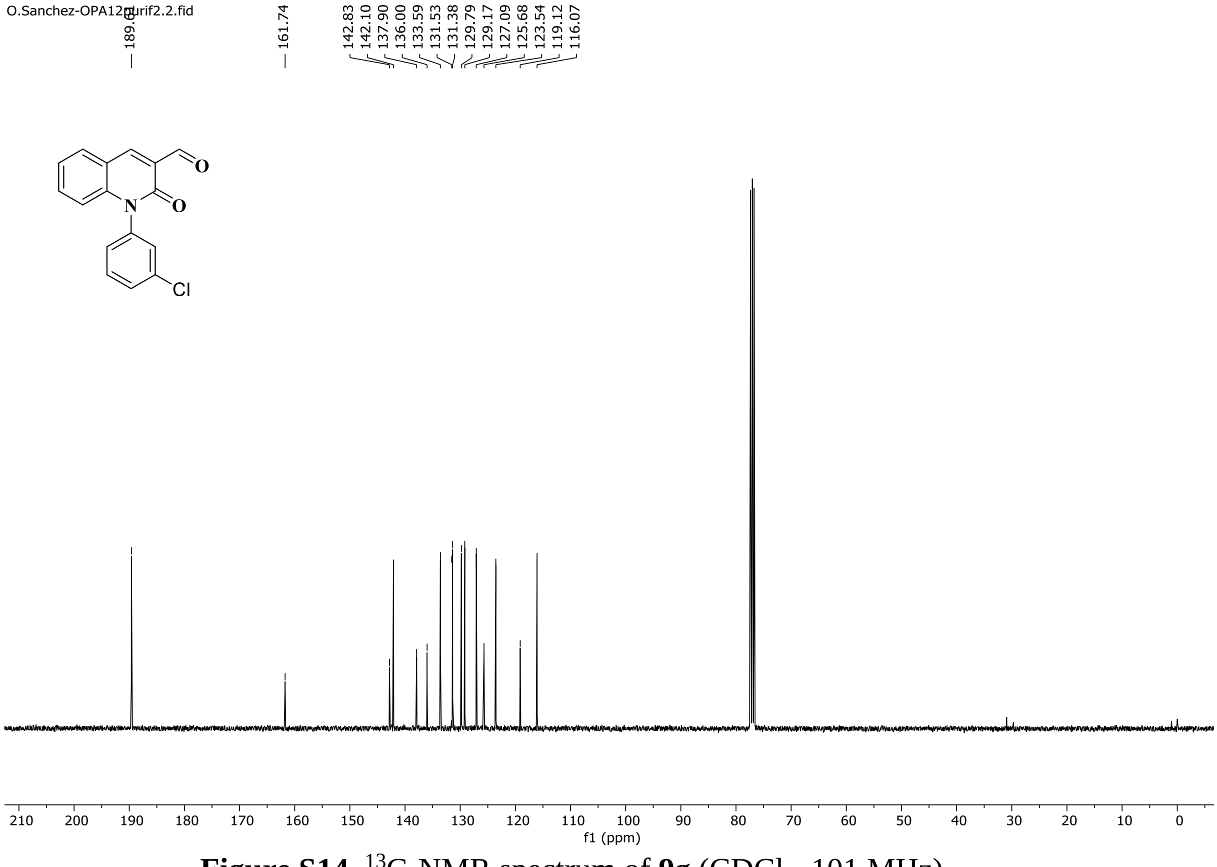

Figure S14. ^{13}C-NMR spectrum of **9g** ($CDCl_3$, 101 MHz)

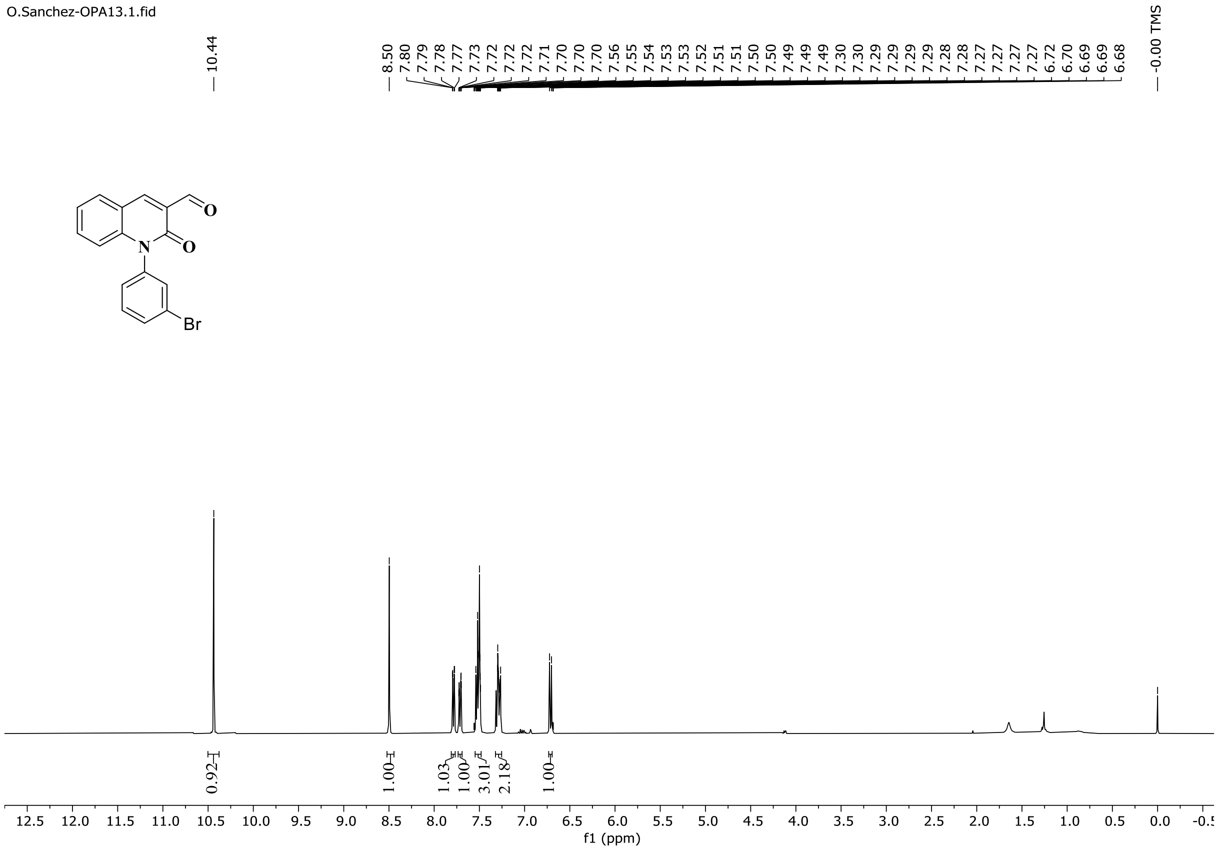

Figure S15. ^{1}H-NMR spectrum of **9h** ($CDCl_3$, 400 MHz)

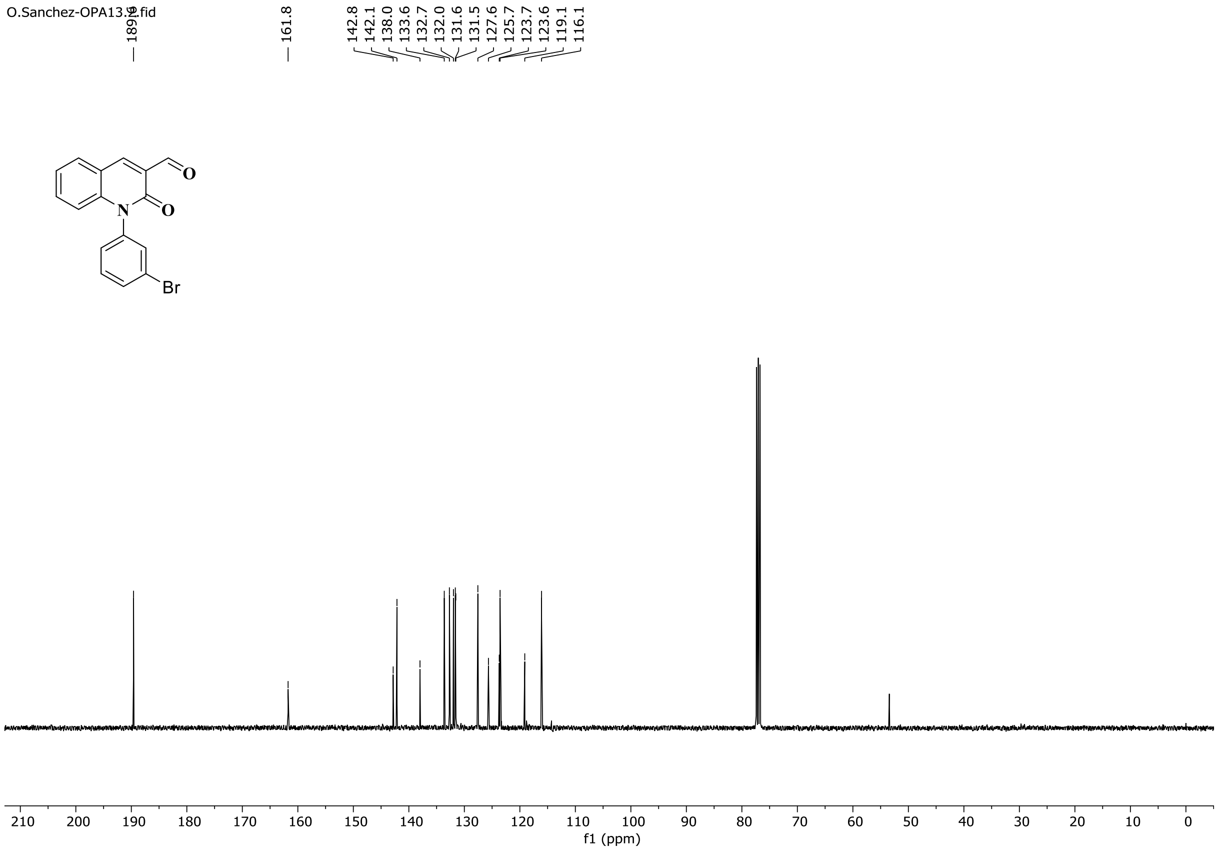

Figure S16. ^{13}C-NMR spectrum of **9h** ($CDCl_3$, 101 MHz)

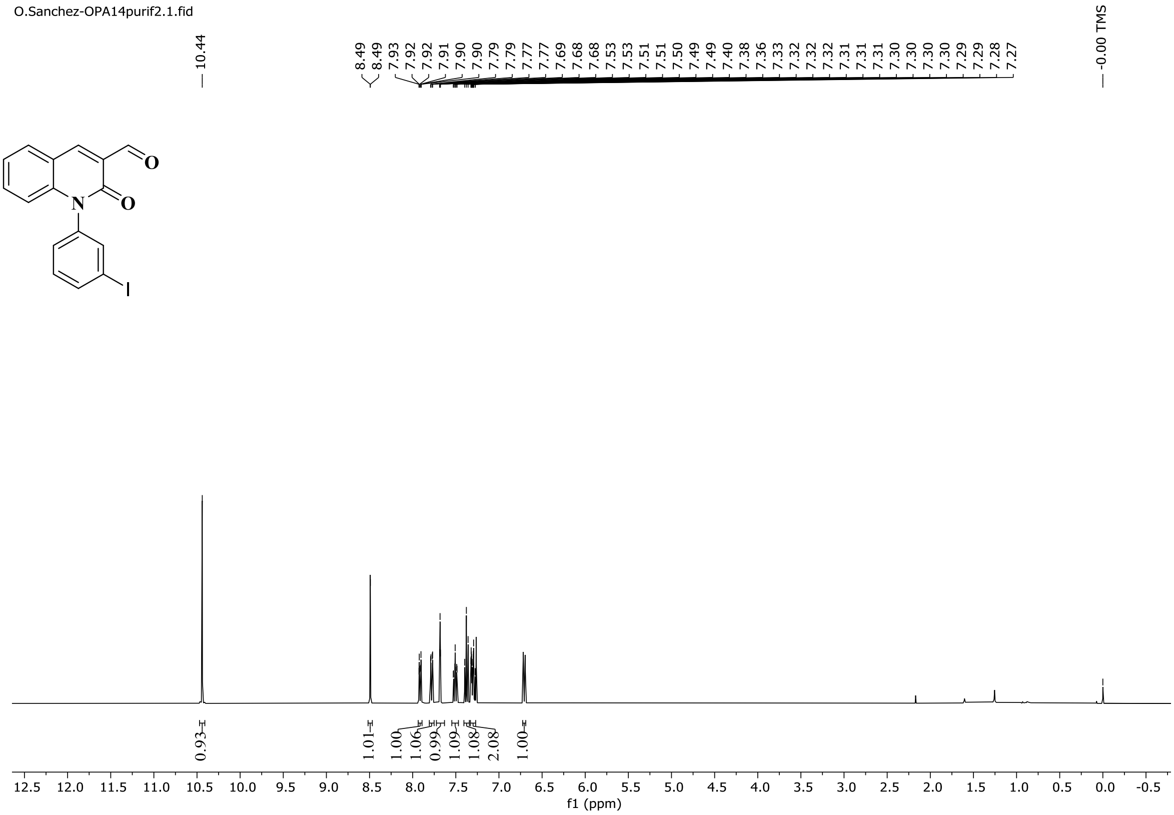

Figure S17. ^{1}H-NMR spectrum of **9i** ($CDCl_3$, 400 MHz)

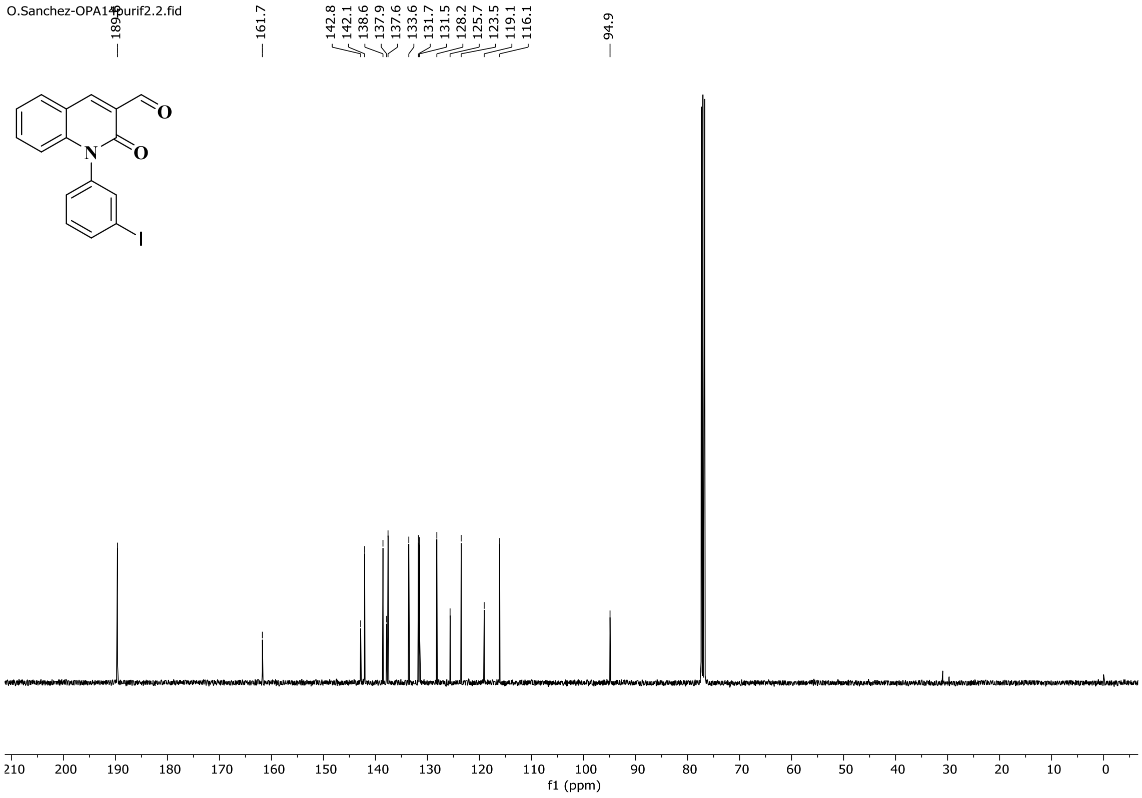

Figure S18. ^{13}C-NMR spectrum of **9i** ($CDCl_3$, 101 MHz)

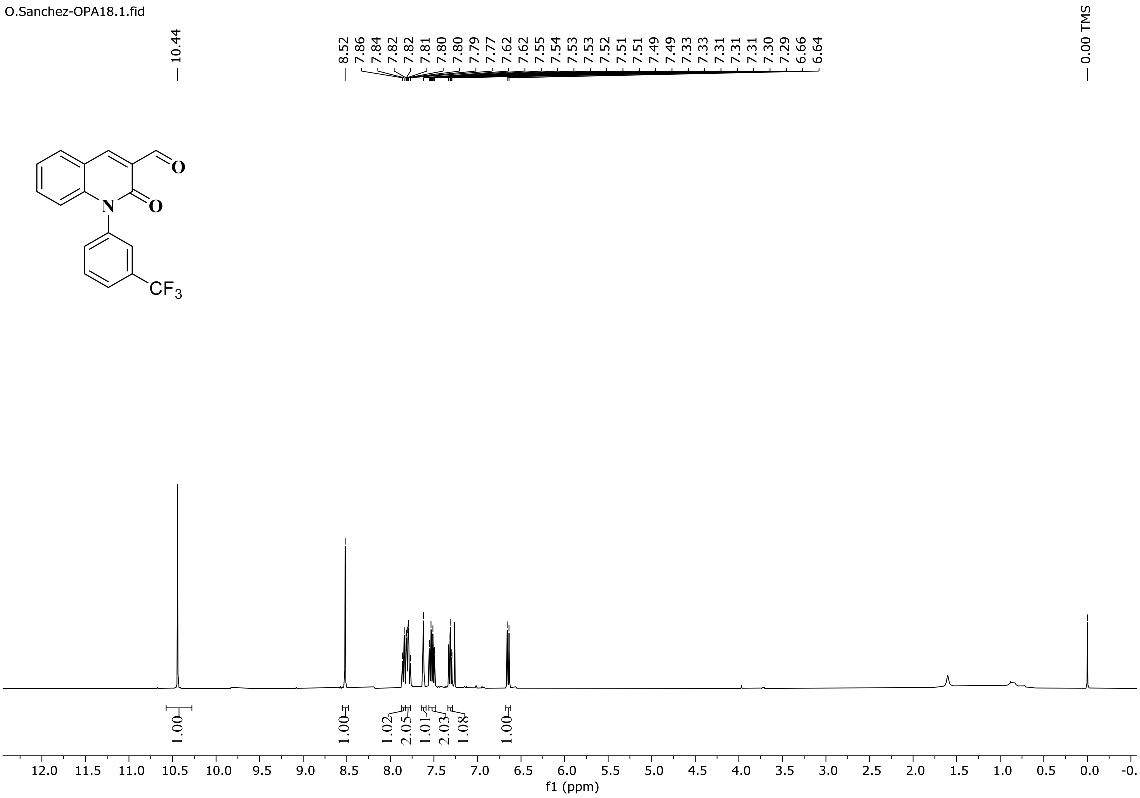

Figure S19. ^{1}H-NMR spectrum of **9j** ($CDCl_3$, 400 MHz)

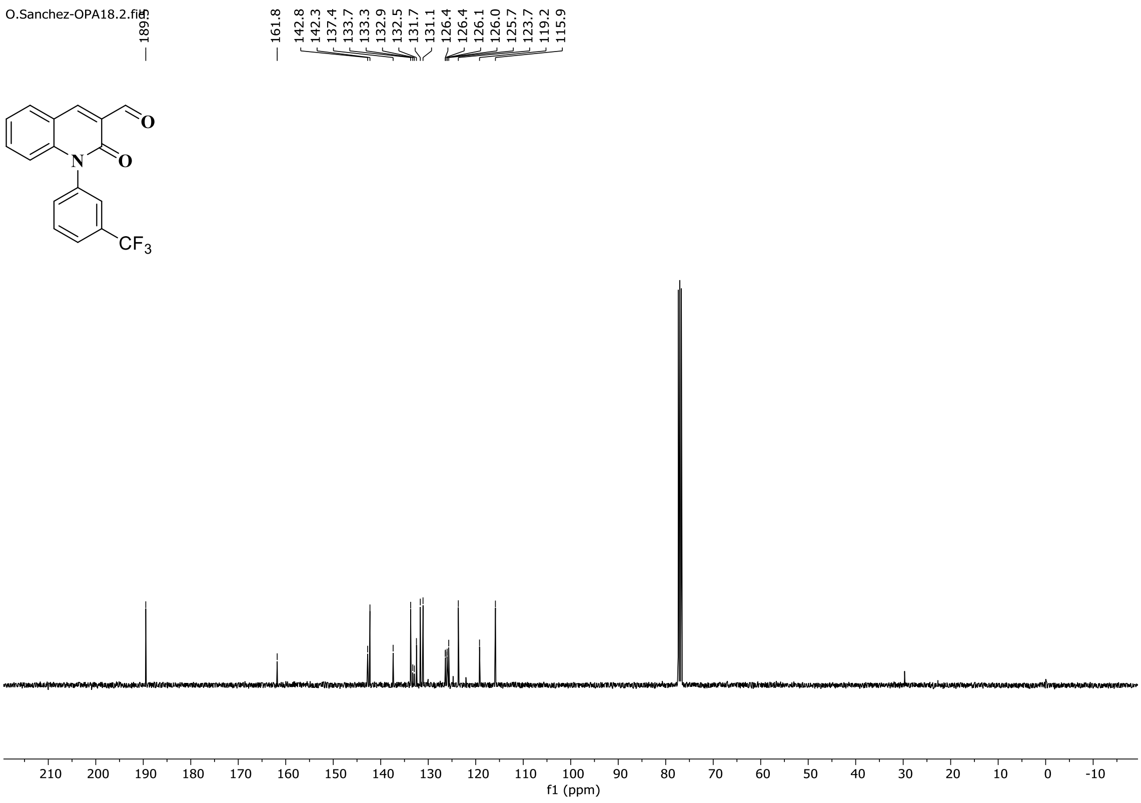

Figure S20. ^{13}C-NMR spectrum of **9j** ($CDCl_3$, 101 MHz)

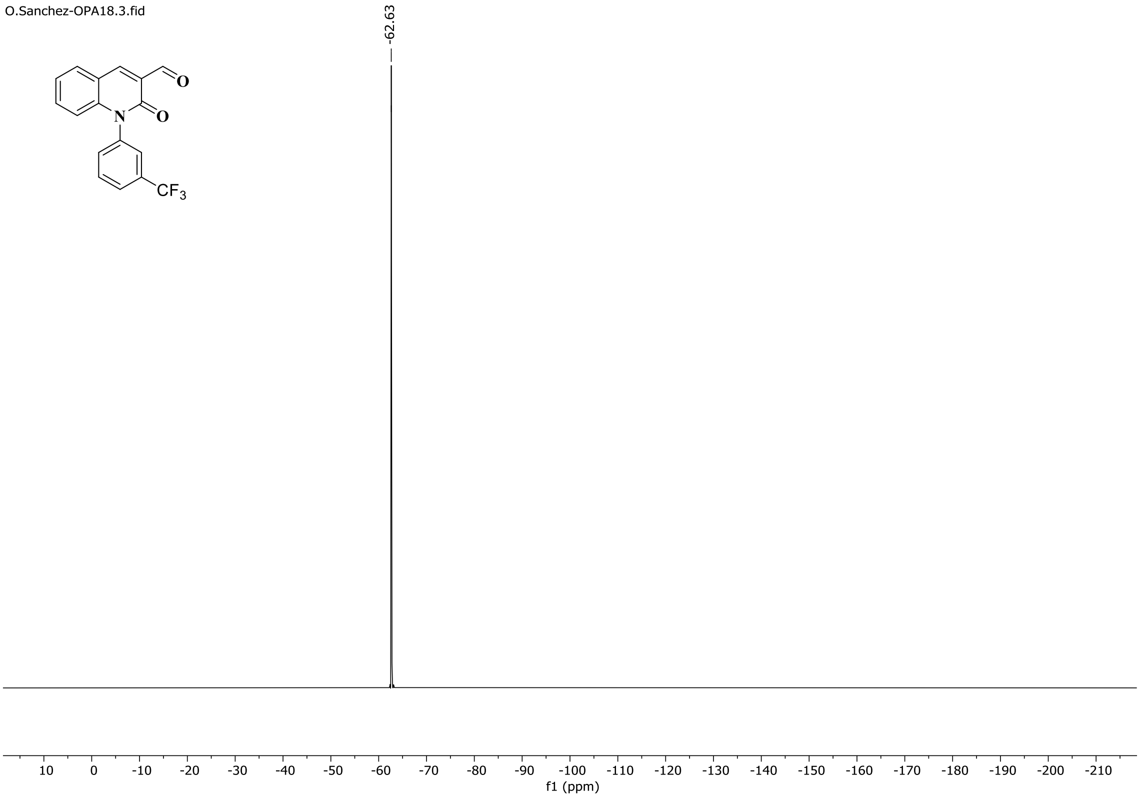

Figure S21. ^{19}F-NMR spectrum of **9j** ($CDCl_3$, 376 MHz)

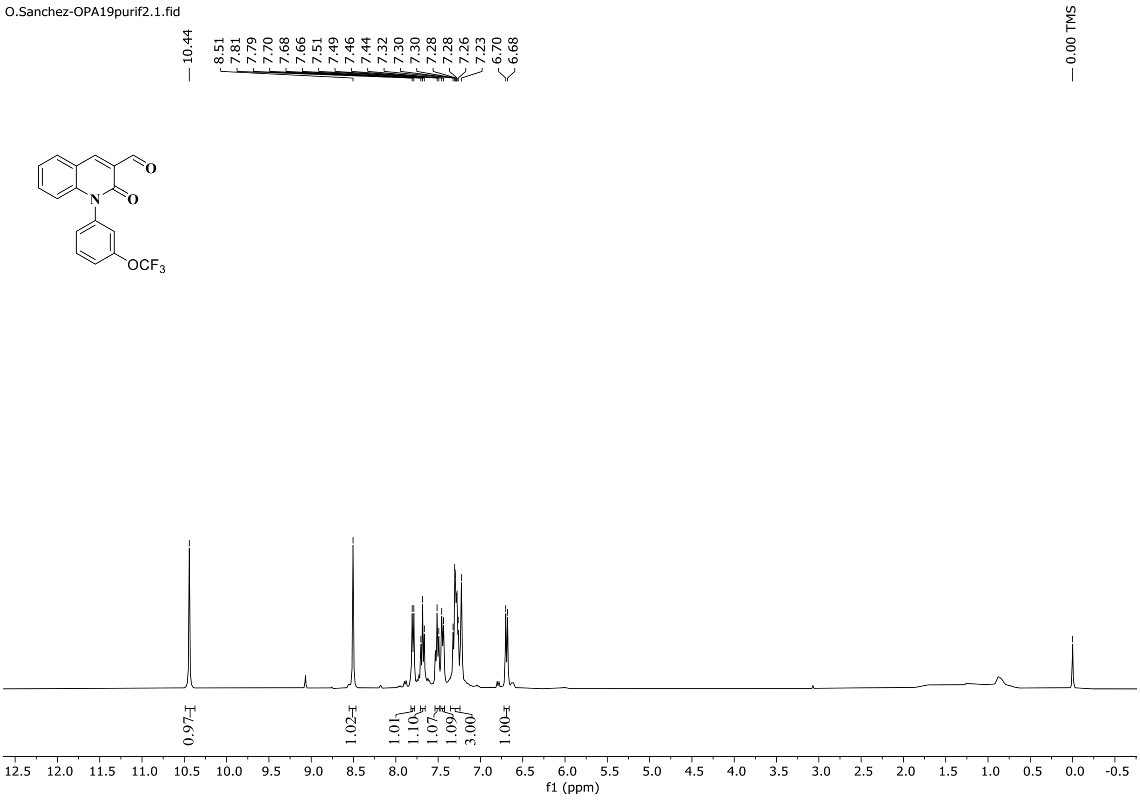

Figure S22. ^{1}H-NMR spectrum of **9k** ($CDCl_3$, 400 MHz)

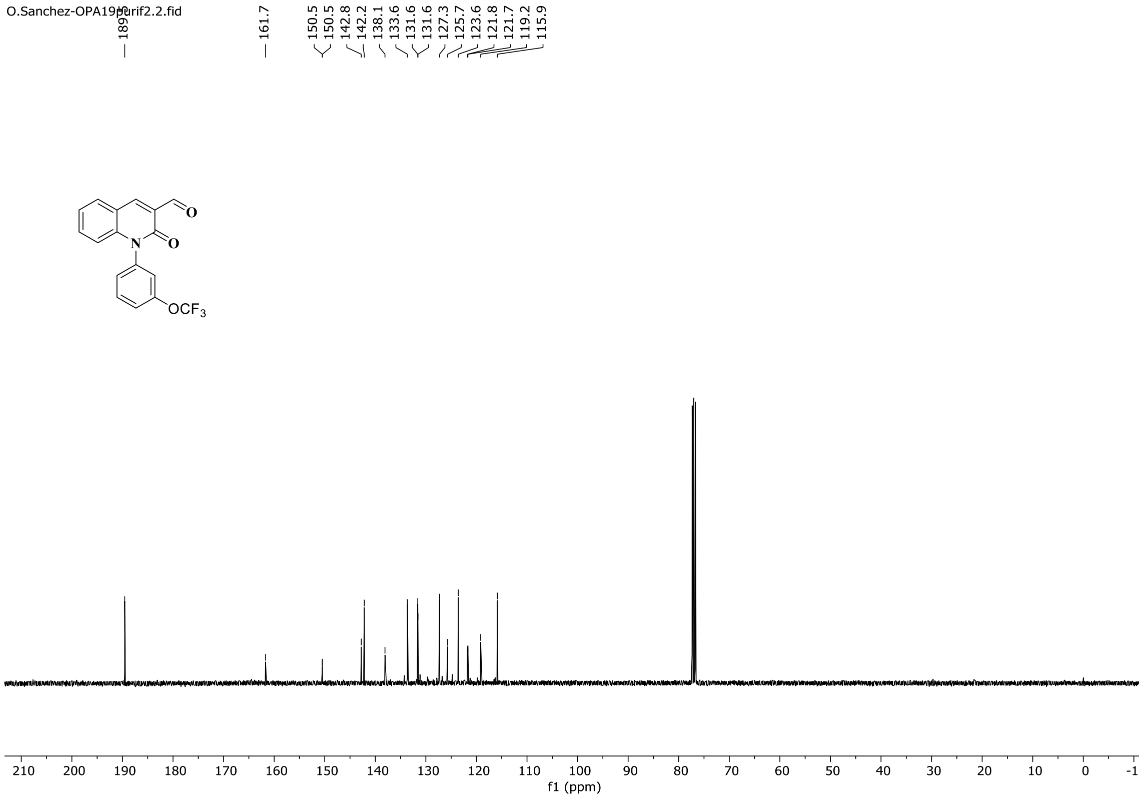

Figure S23. ^{13}C-NMR spectrum of **9k** ($CDCl_3$, 101 MHz)

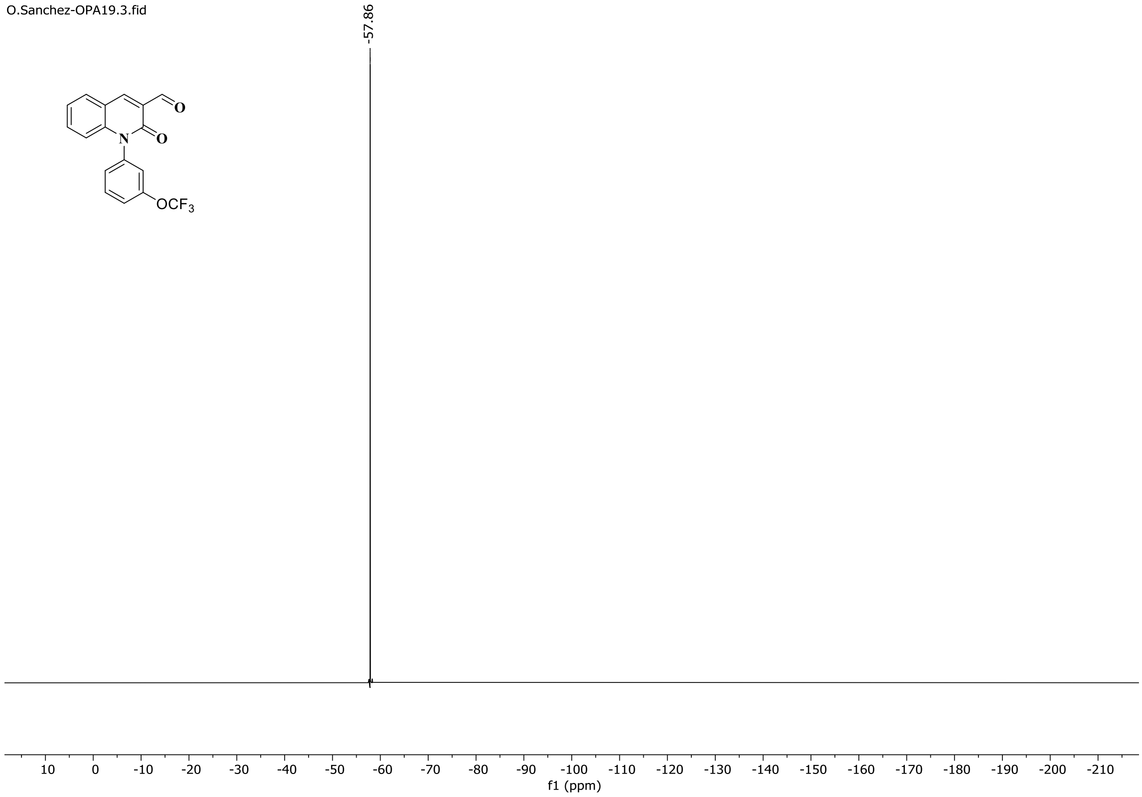

Figure S24. ^{19}F-NMR spectrum of **9k** ($CDCl_3$, 376 MHz)

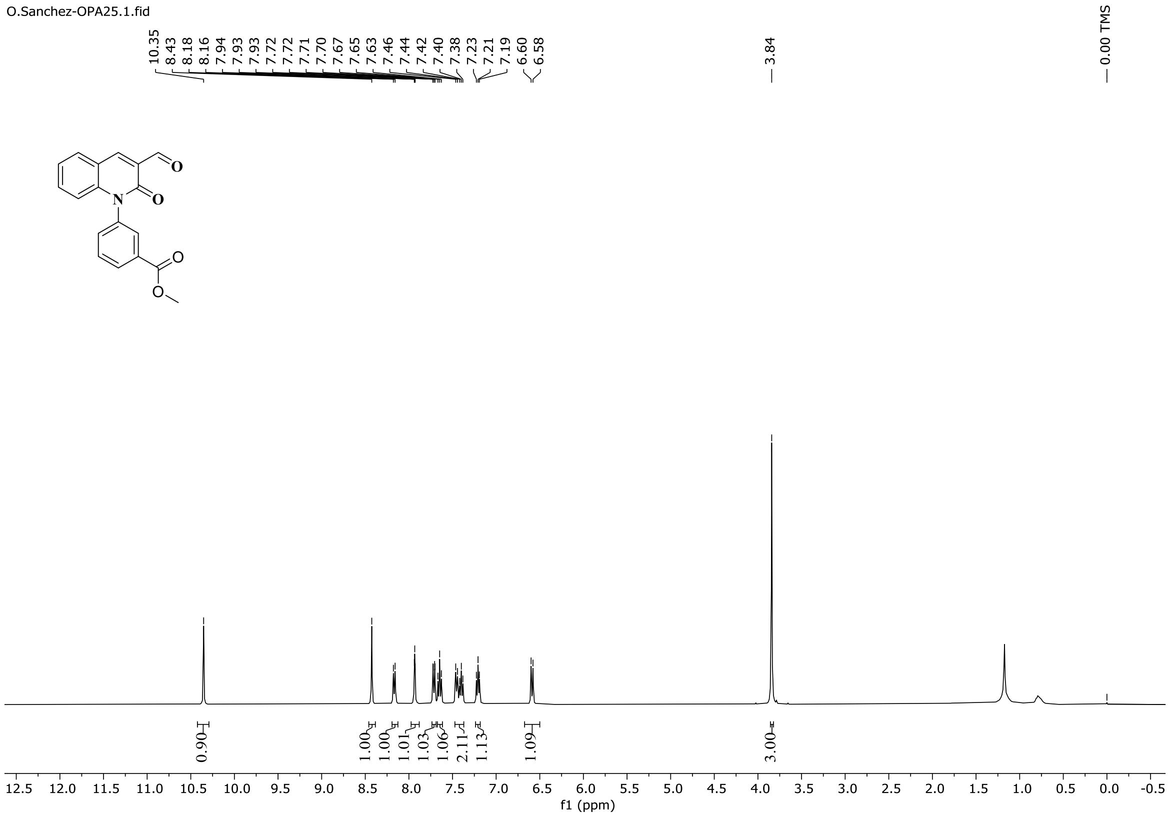

Figure S25. ^{1}H-NMR spectrum of **9l** ($CDCl_3$, 400 MHz)

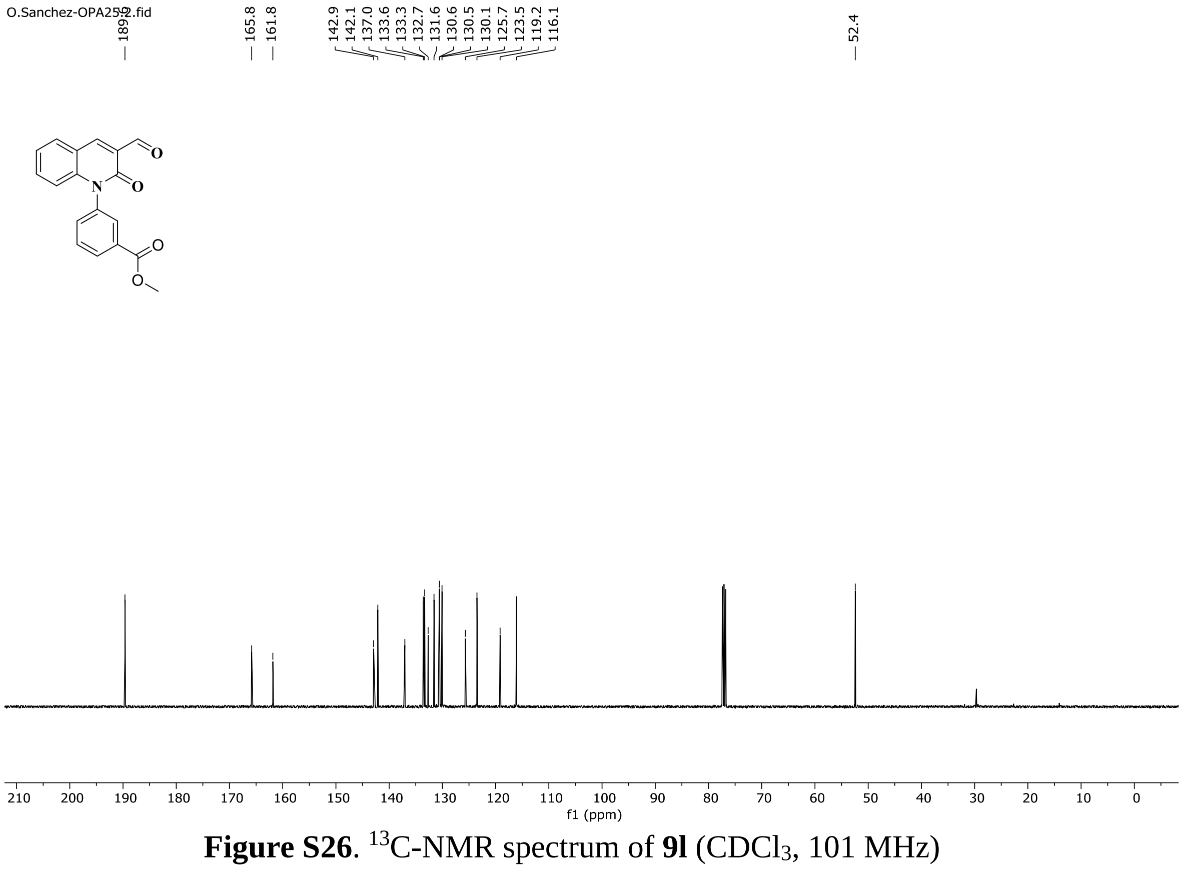

Figure S26. ^{13}C-NMR spectrum of **9l** ($CDCl_3$, 101 MHz)

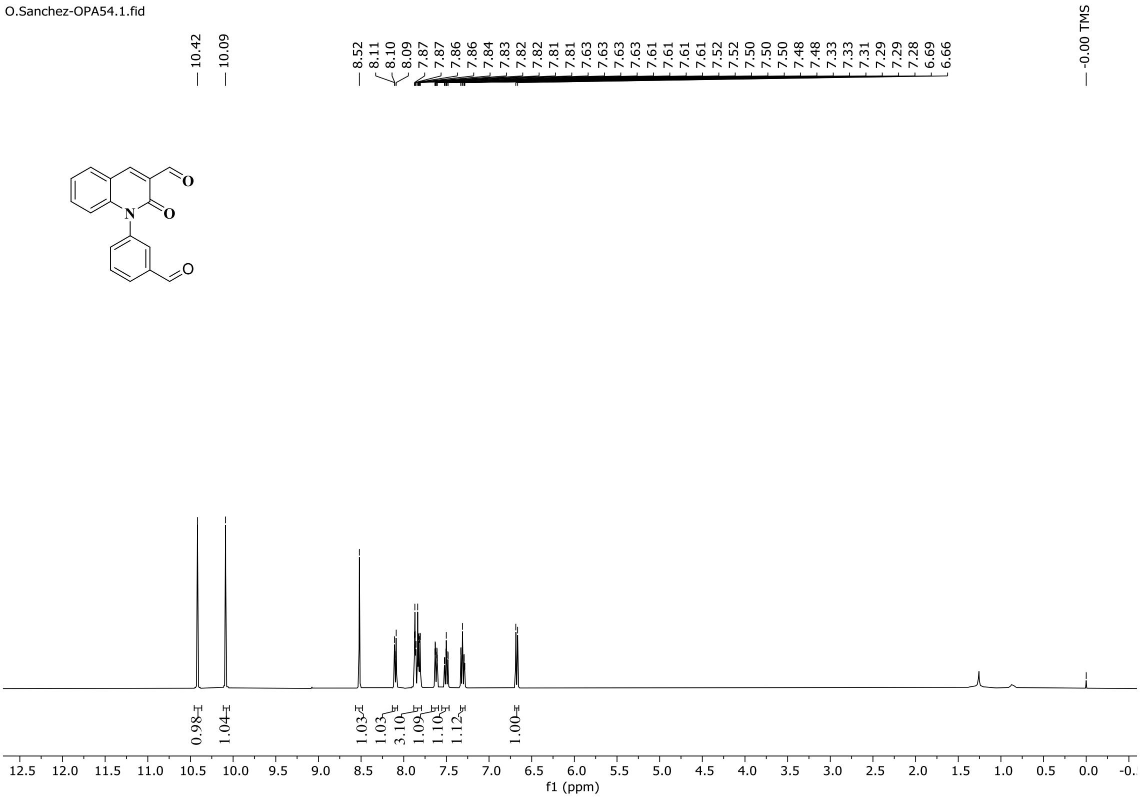

Figure S27. ^{1}H-NMR spectrum of **9m** ($CDCl_3$, 400 MHz)

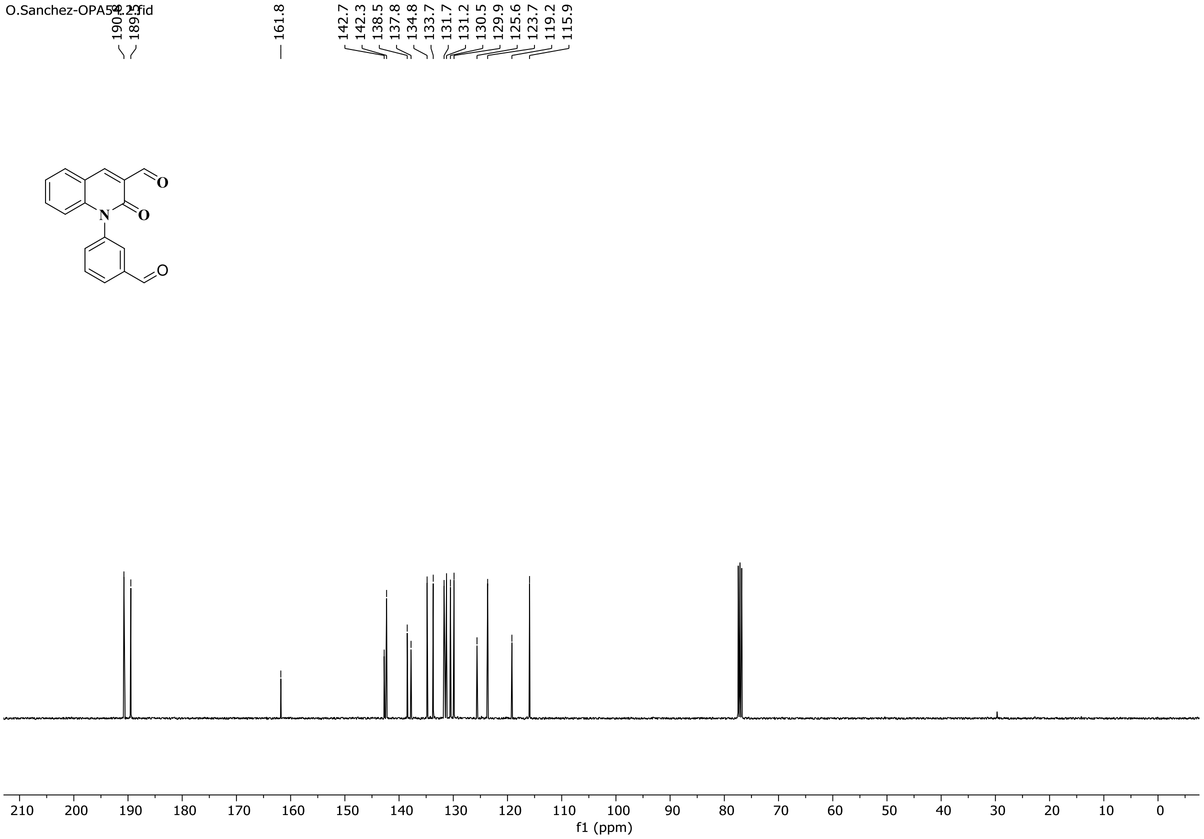

Figure S28. ^{13}C-NMR spectrum of **9m** ($CDCl_3$, 101 MHz)

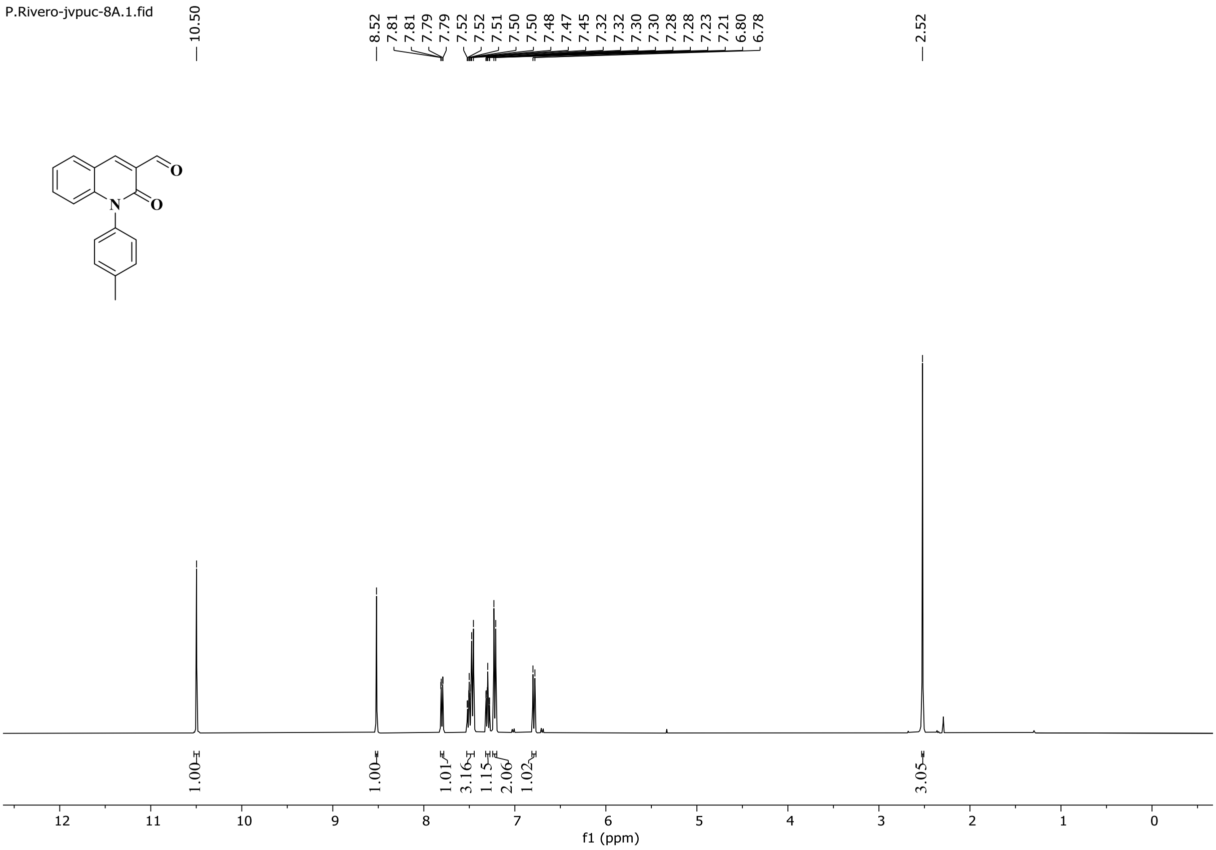

Figure S29. ^{1}H-NMR spectrum of **9a** ($CDCl_3$, 400 MHz)

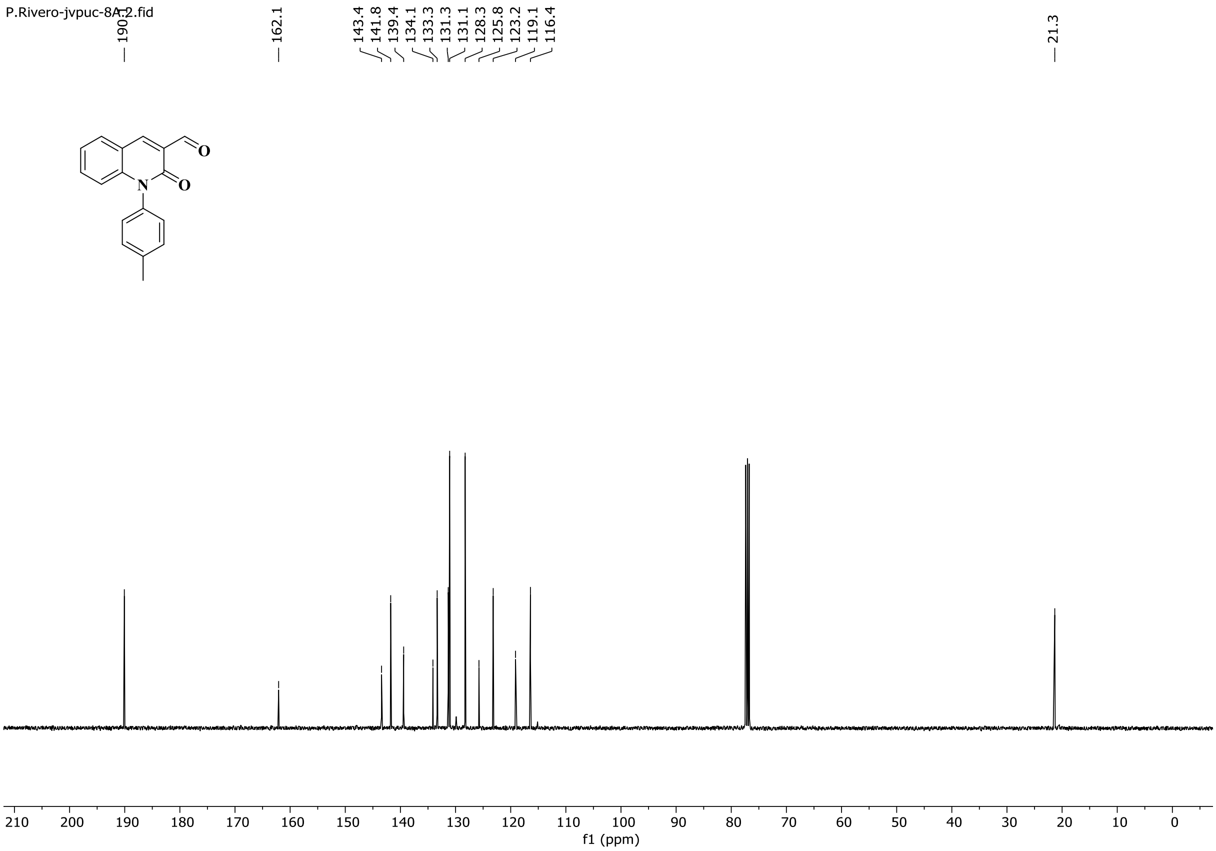

Figure S30. ^{13}C-NMR spectrum of **9a** ($CDCl_3$, 101 MHz)

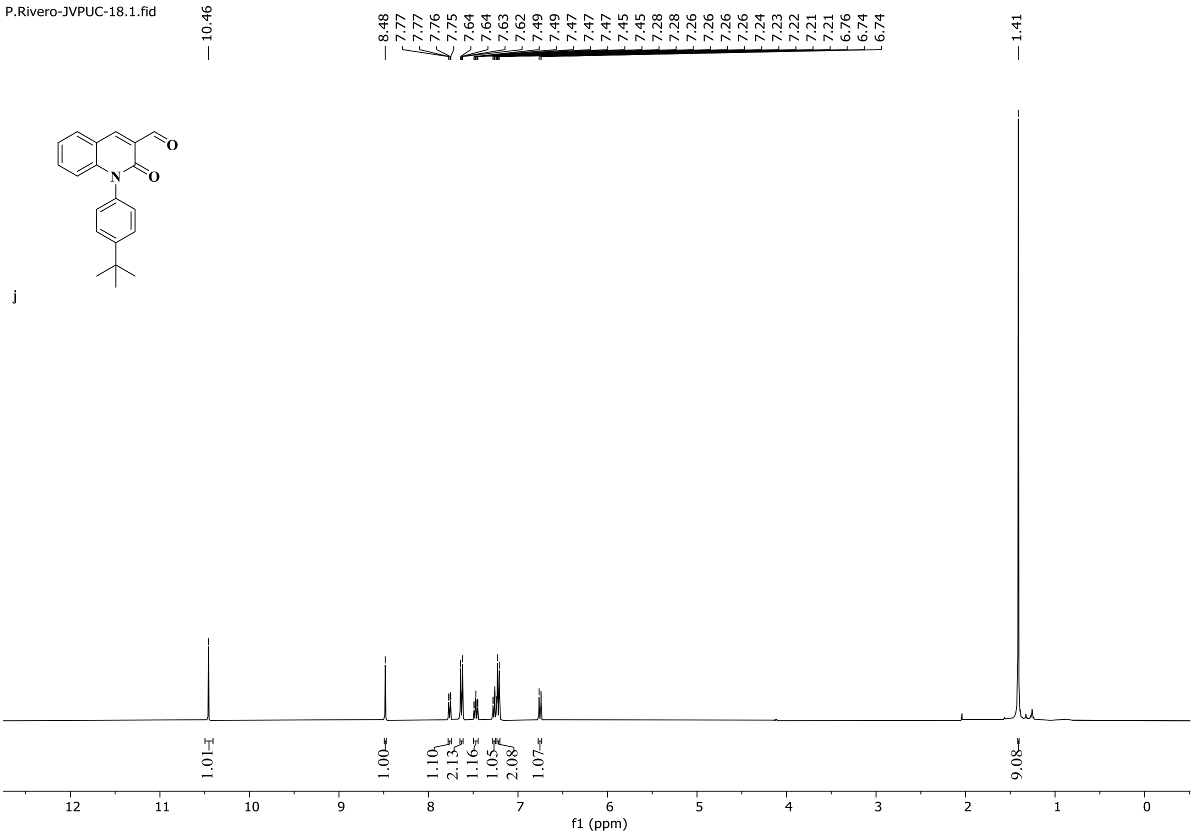

Figure S31. ^{1}H-NMR spectrum of **9n** ($CDCl_3$, 400 MHz)

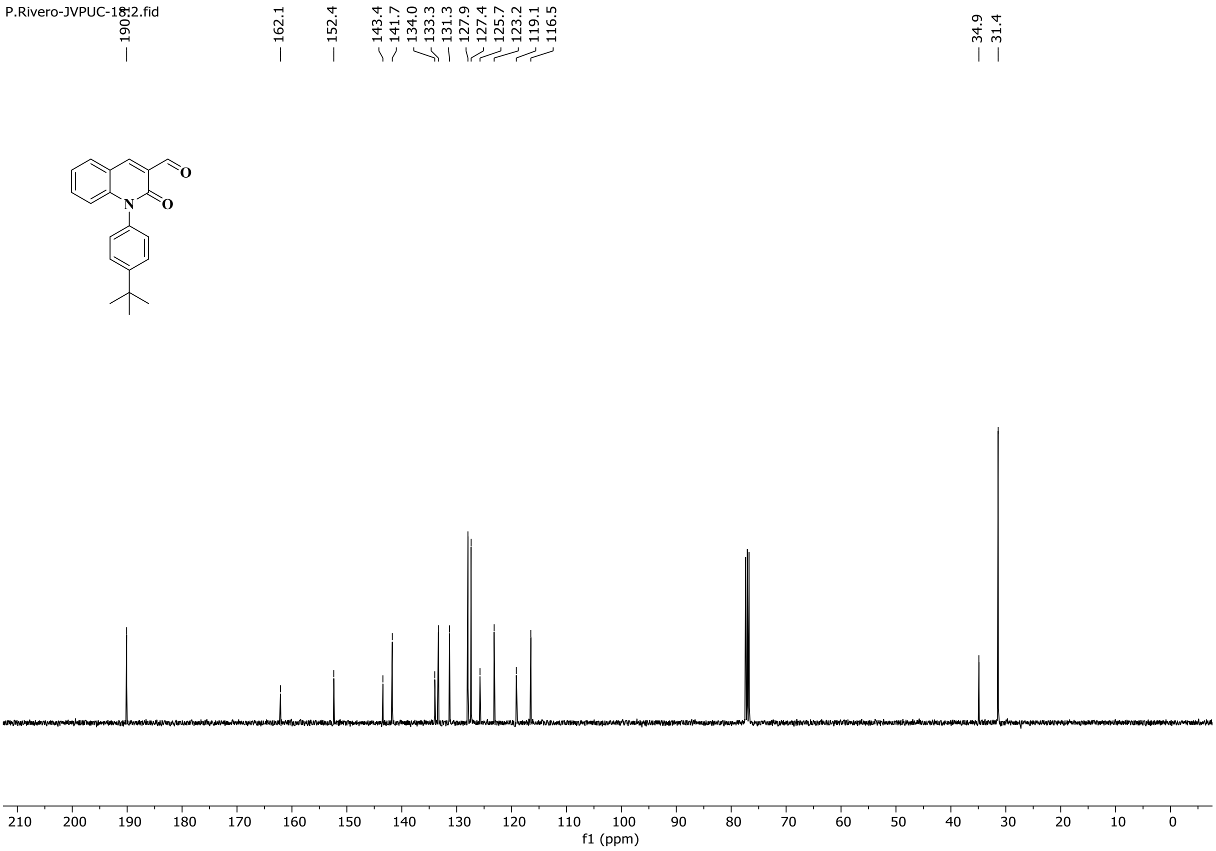

Figure S32. ^{13}C-NMR spectrum of **9n** ($CDCl_3$, 101 MHz)

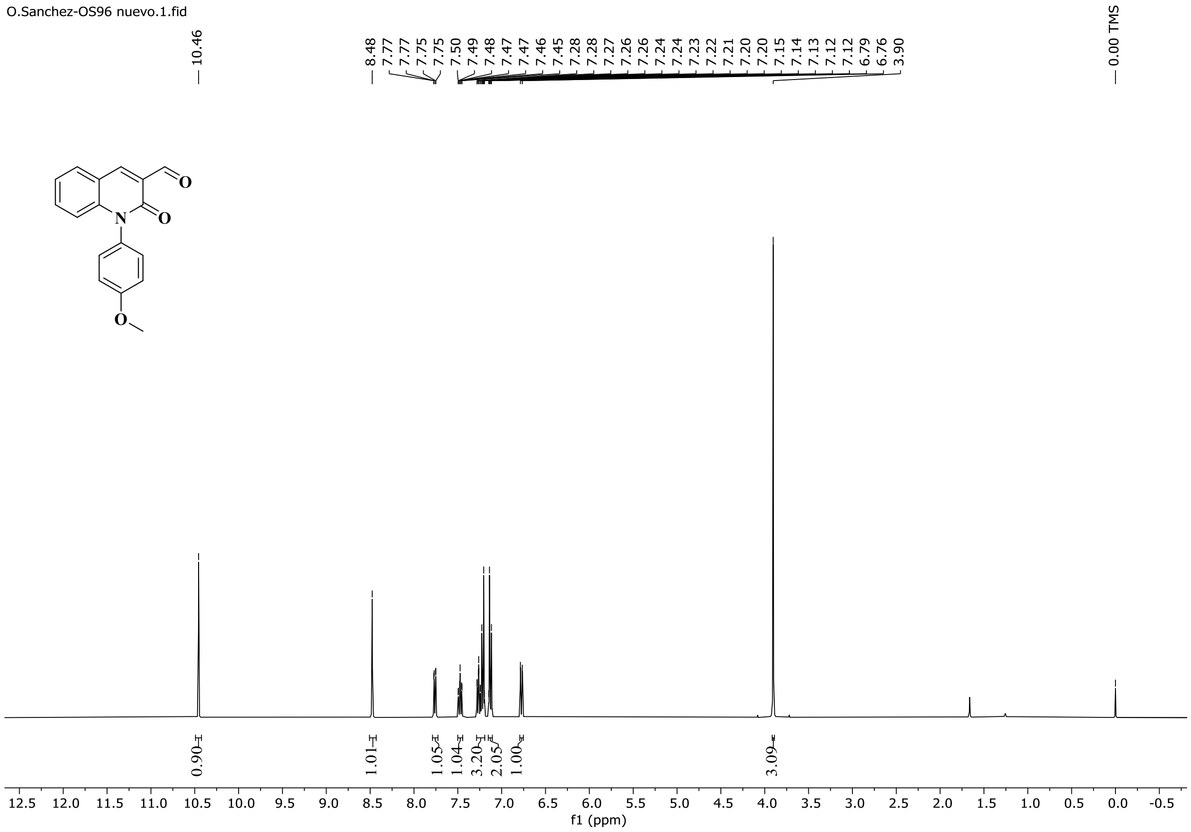

Figure S33. ^{1}H-NMR spectrum of **9o**(CDCl$_3$, 400 MHz)

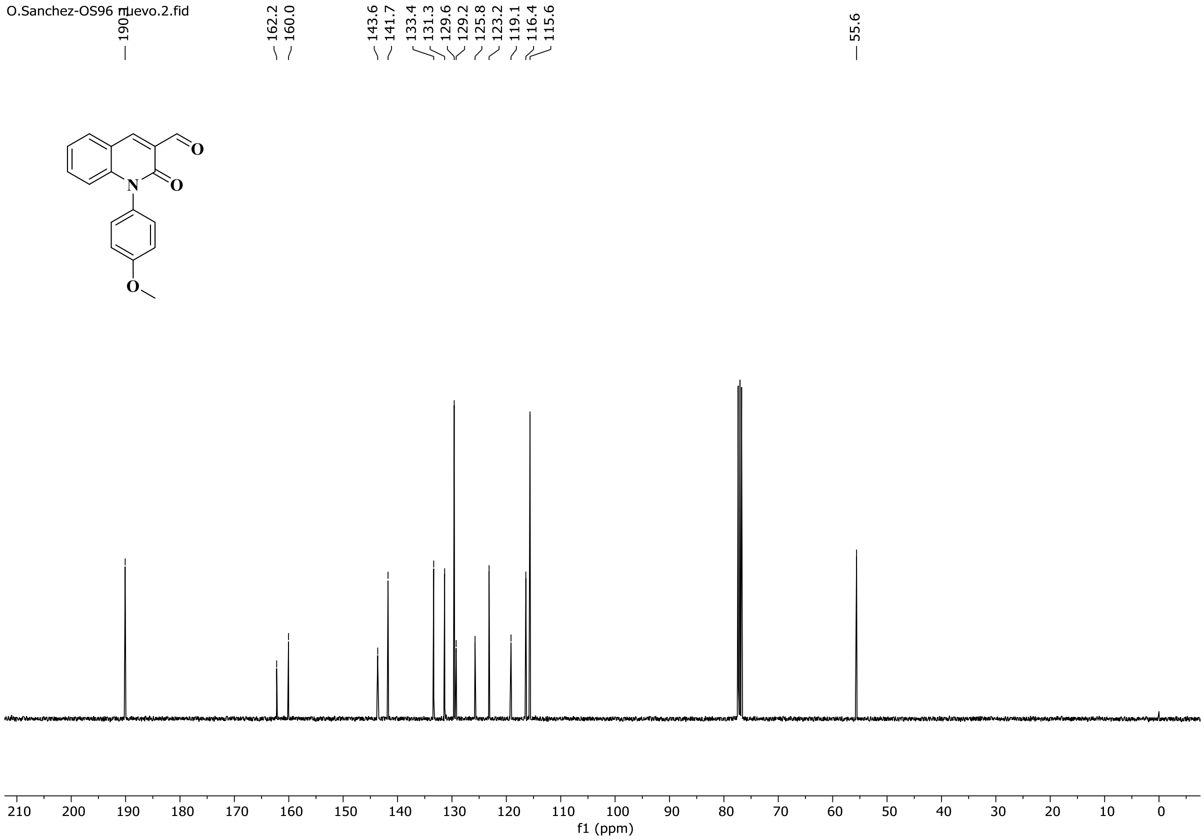

Figure S34. ^{13}C-NMR spectrum of **9o** (CDCl$_3$, 101 MHz)

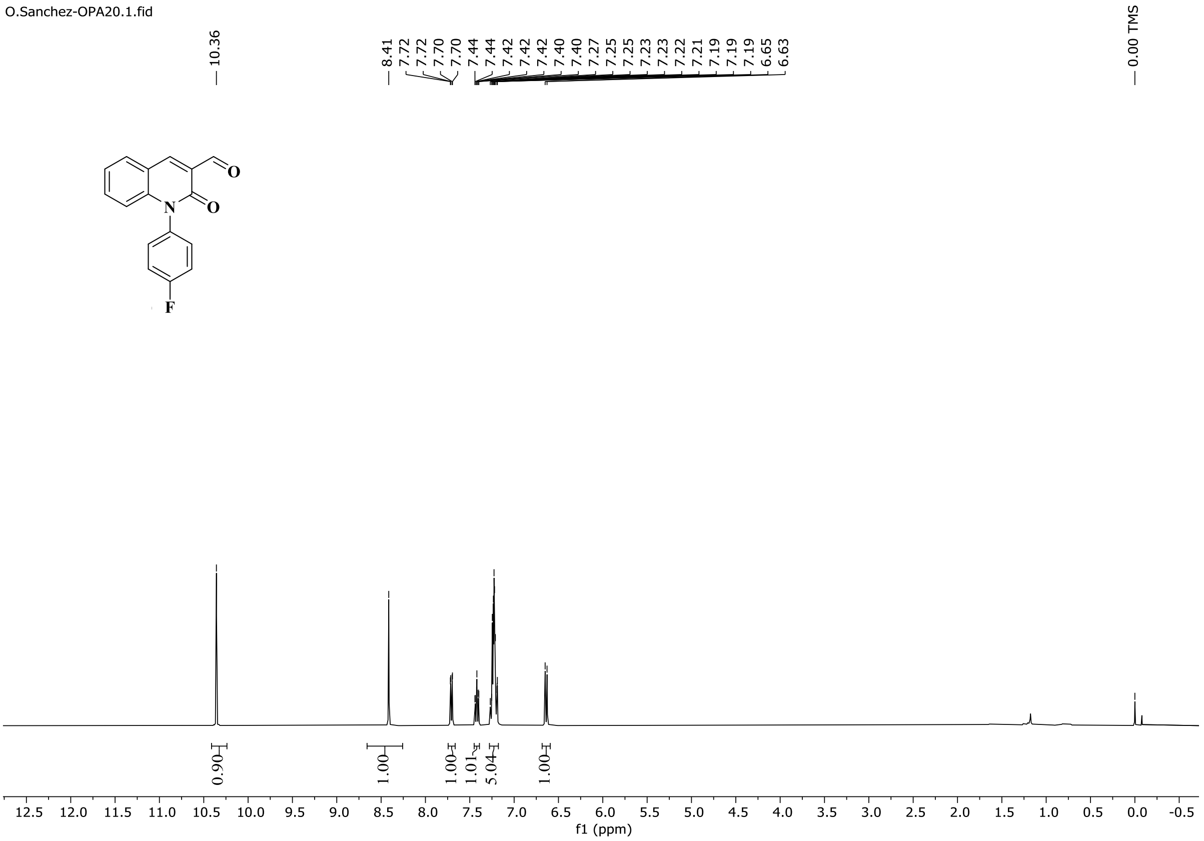

Figure S35. ^{1}H-NMR spectrum of **9p** ($CDCl_3$, 400 MHz)

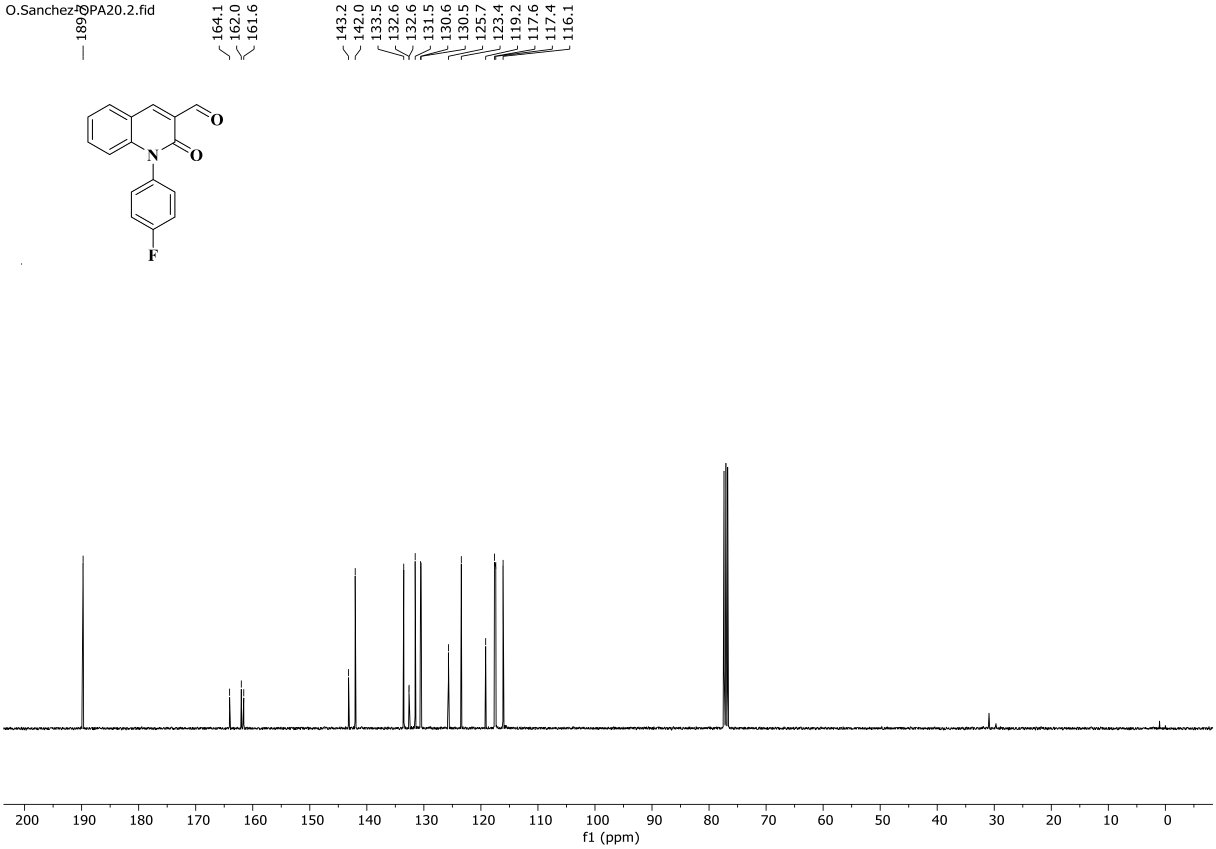

Figure S36. ^{13}C-NMR spectrum of **9p** ($CDCl_3$, 101 MHz)

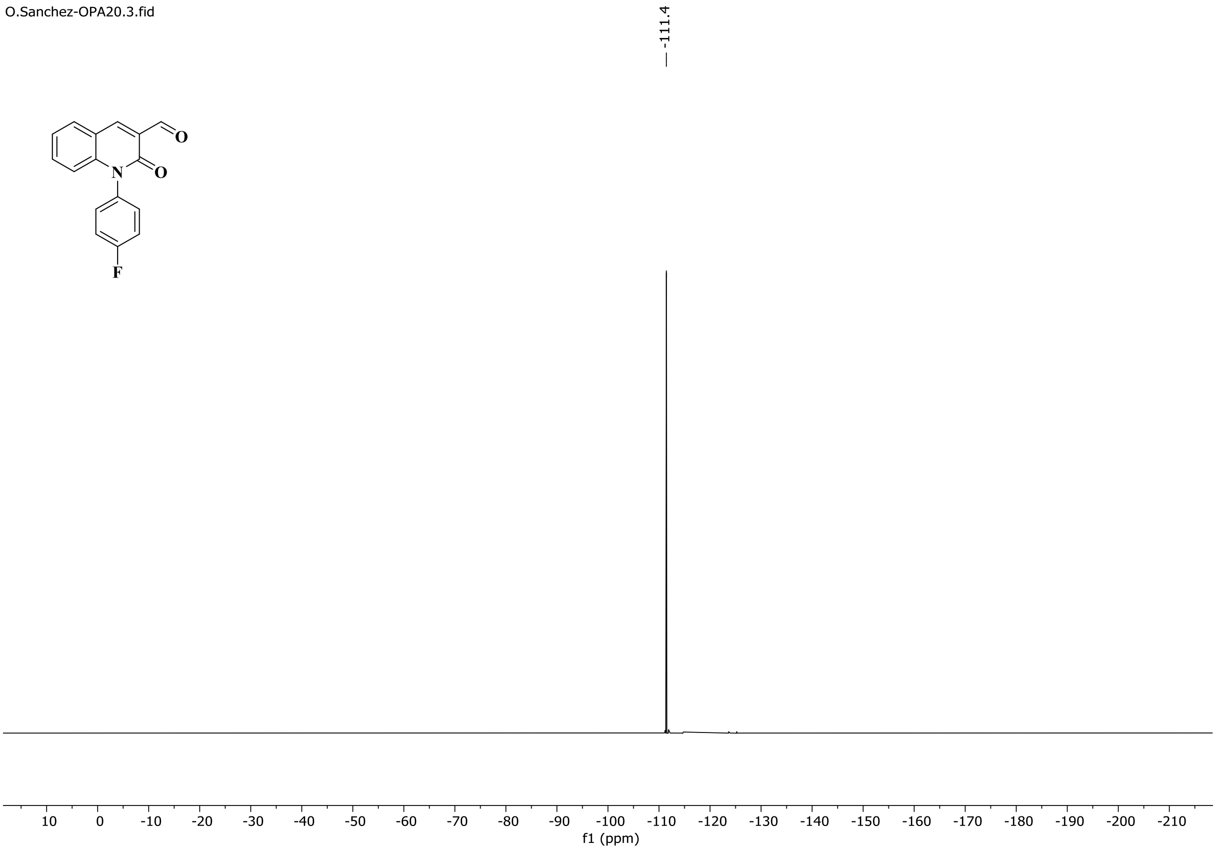

Figure S37. ^{19}F-NMR spectrum of **9p** ($CDCl_3$, 376 MHz)

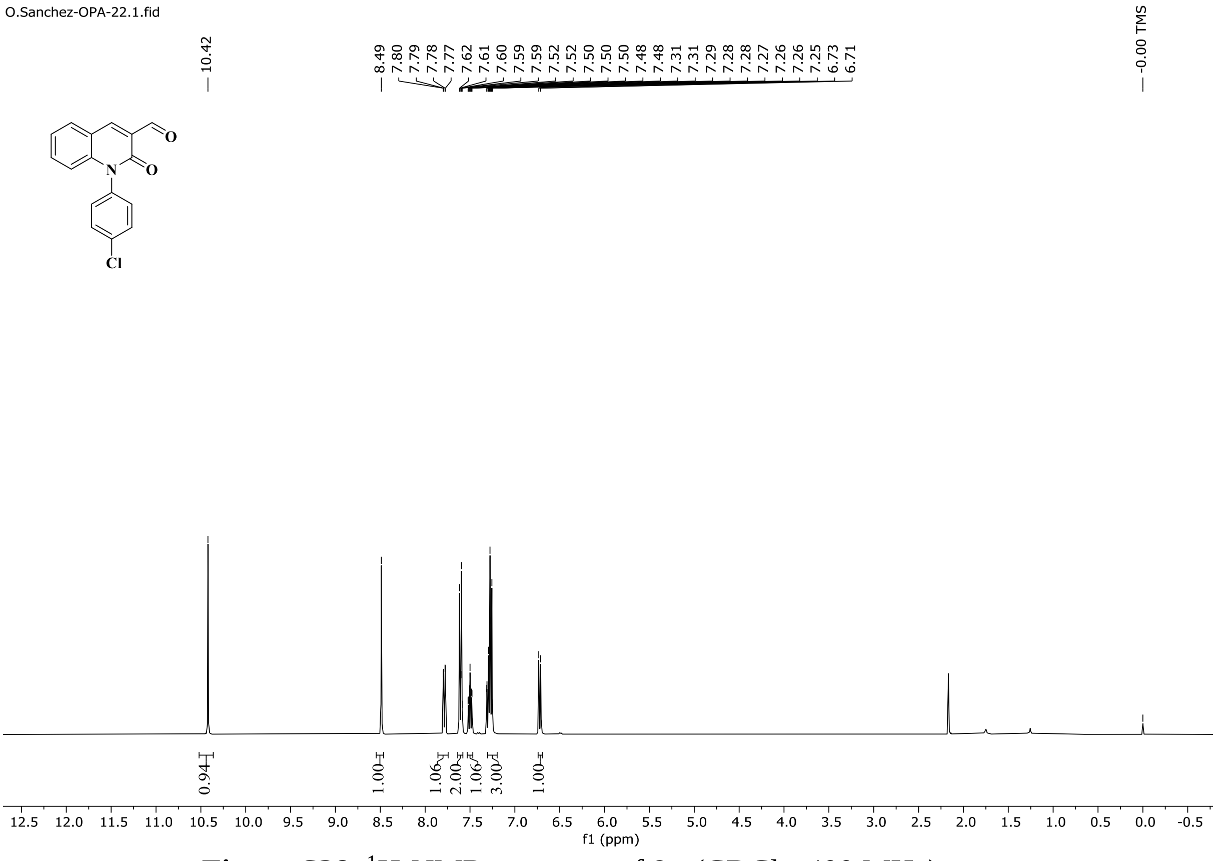

Figure S38. ^{1}H-NMR spectrum of **9q** ($CDCl_3$, 400 MHz)

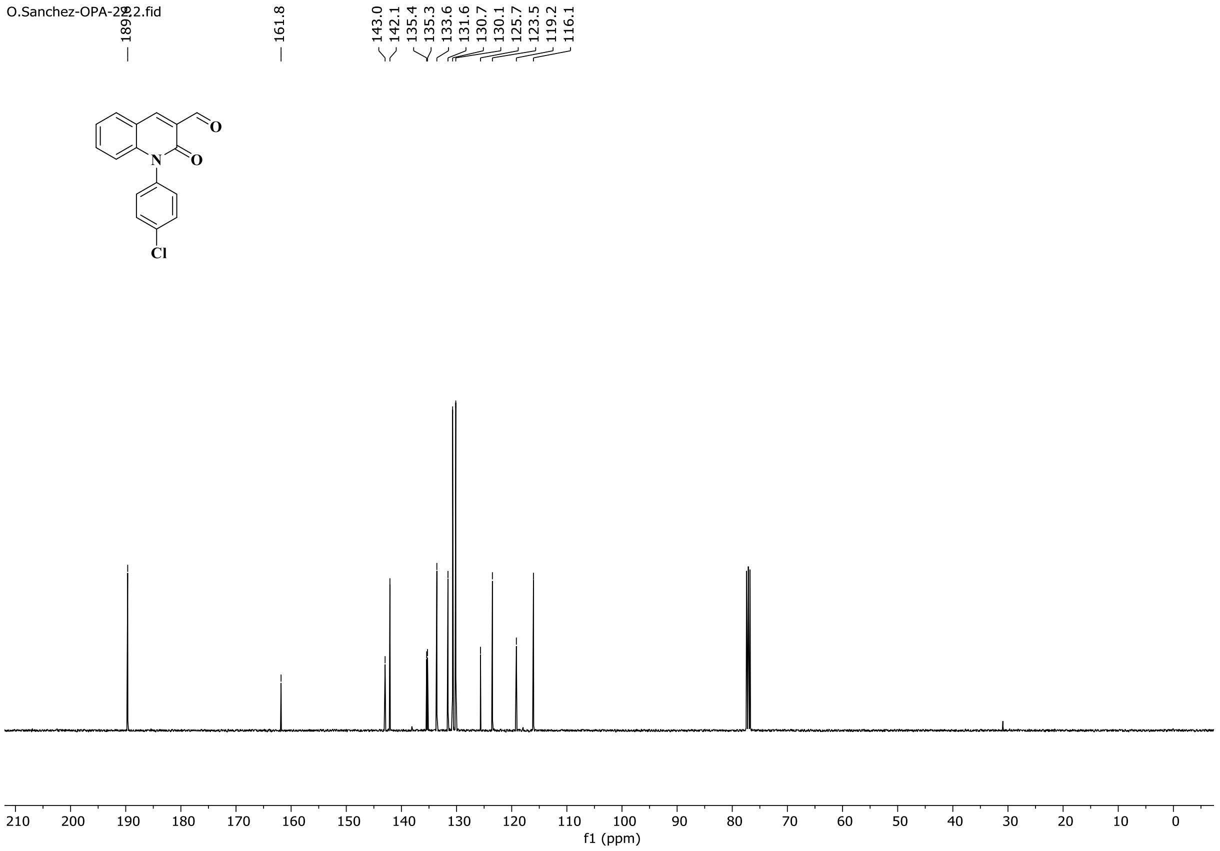

Figure S39. ^{13}C-NMR spectrum of **9q** ($CDCl_3$, 101 MHz)

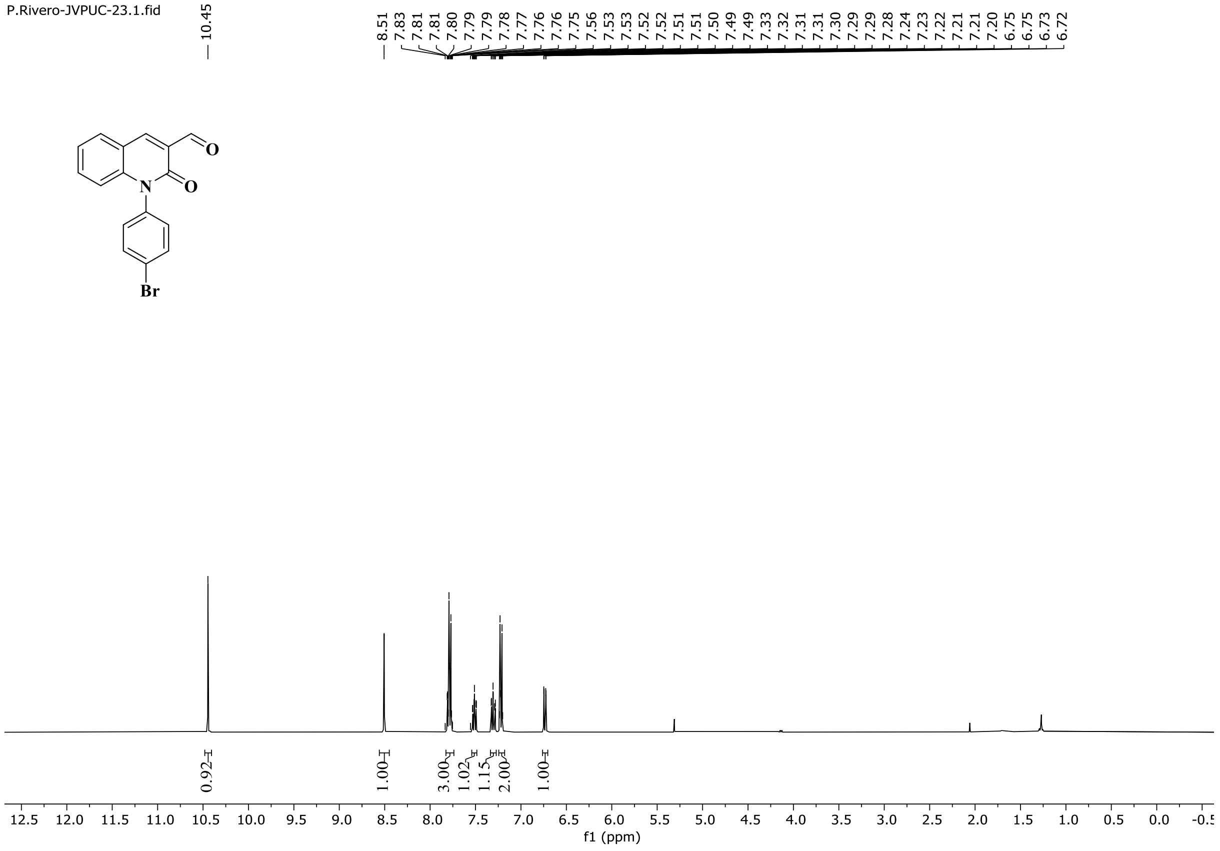

Figure S40. ^{1}H-NMR spectrum of **9r** ($CDCl_3$, 400 MHz)

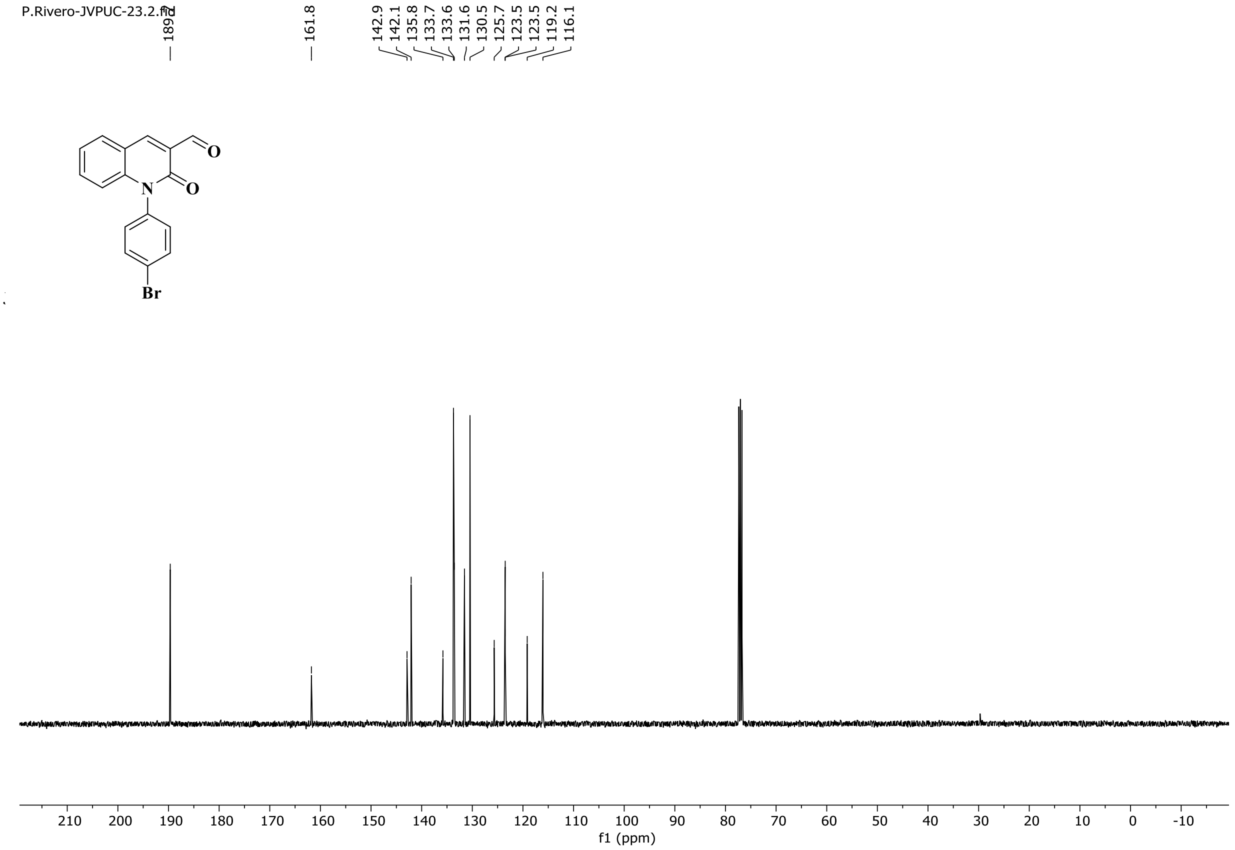

Figure S41. ^{13}C-NMR spectrum of **9r** ($CDCl_3$, 101 MHz)

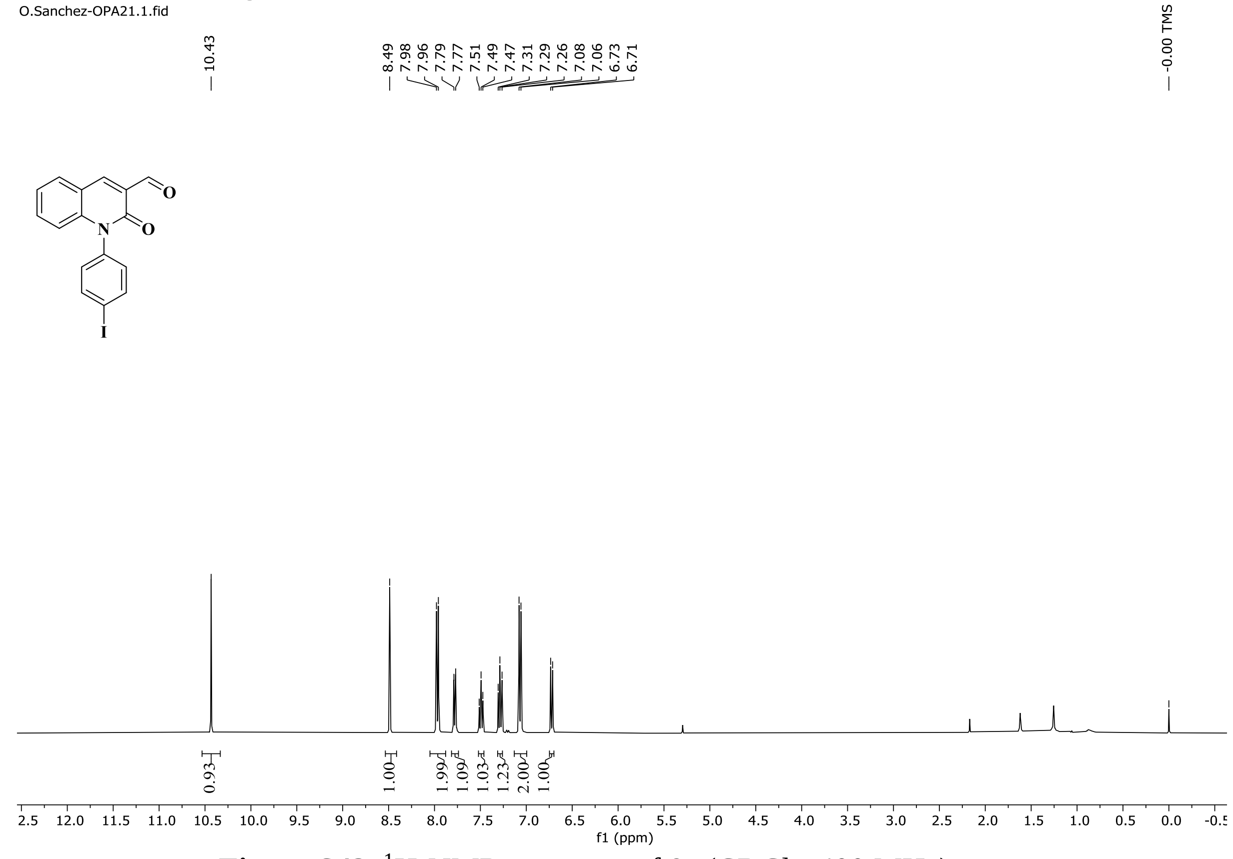

Figure S42. ^{1}H-NMR spectrum of **9s** ($CDCl_3$, 400 MHz)

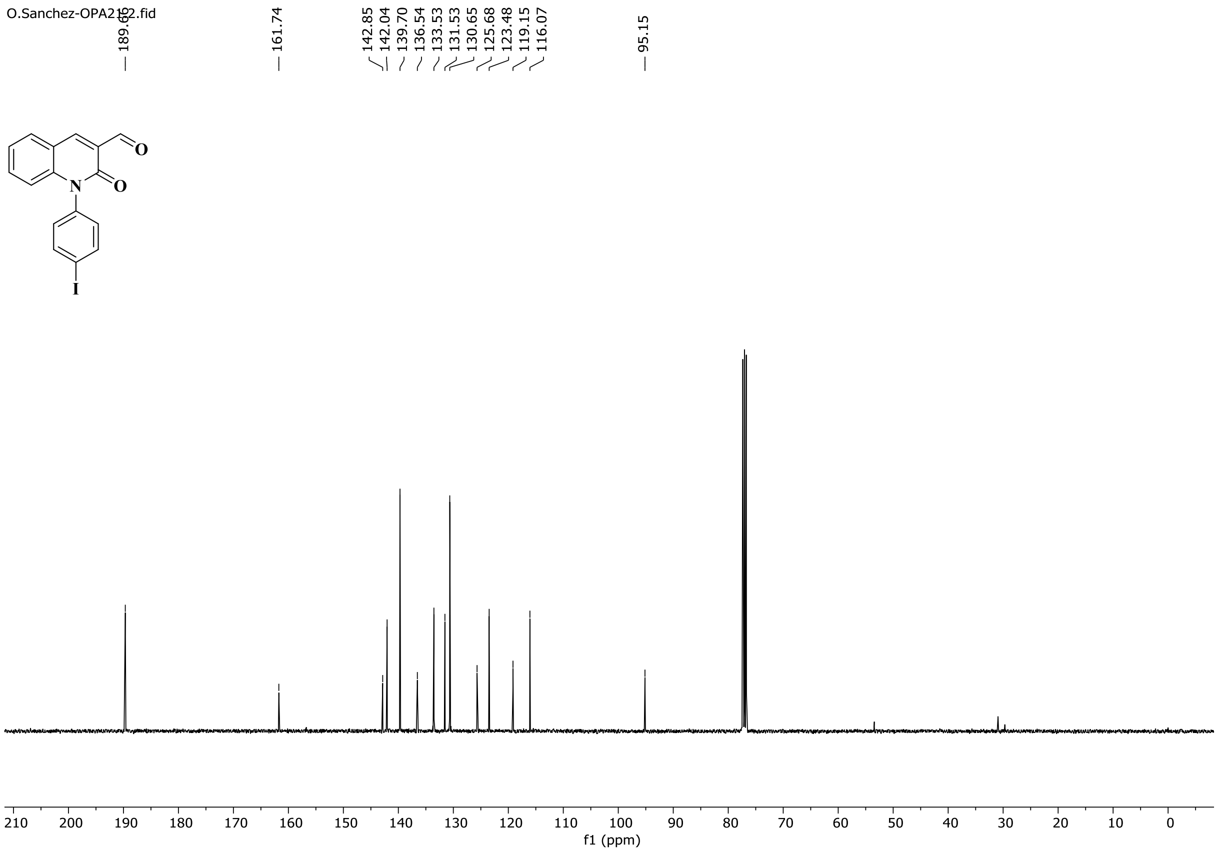

Figure S43. ^{13}C-NMR spectrum of **9s** ($CDCl_3$, 101 MHz)

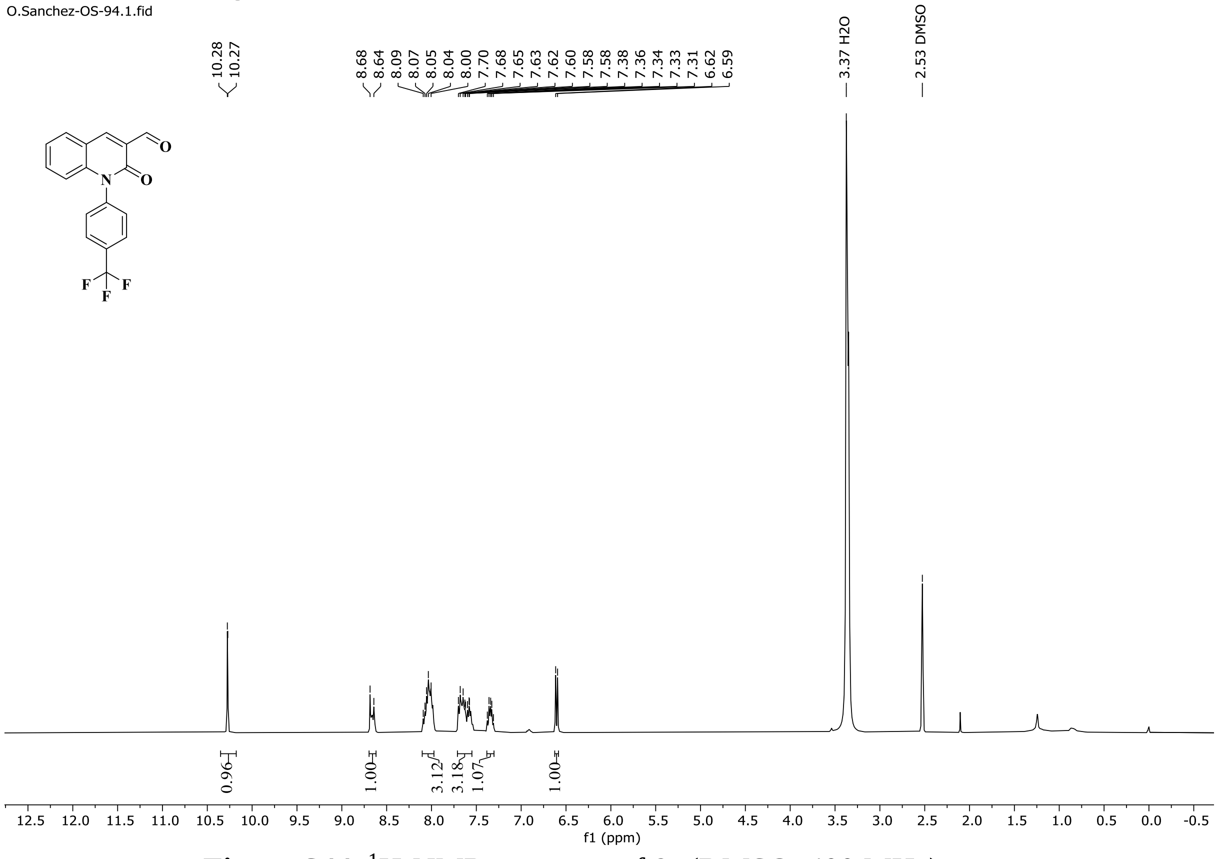

Figure S44. ^{1}H-NMR spectrum of **9t** (DMSO, 400 MHz)

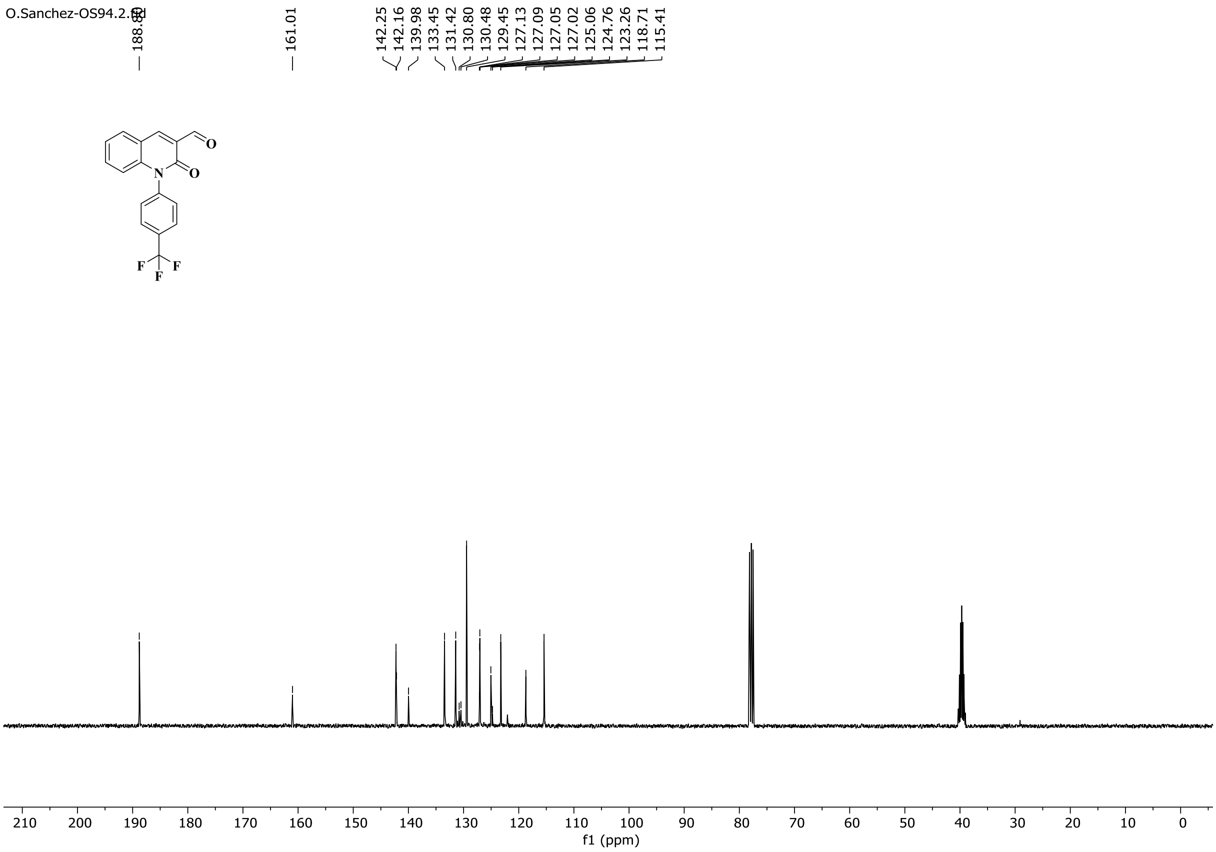

Figure S45. ^{13}C-NMR spectrum of **9t** (DMSO, 101 MHz)

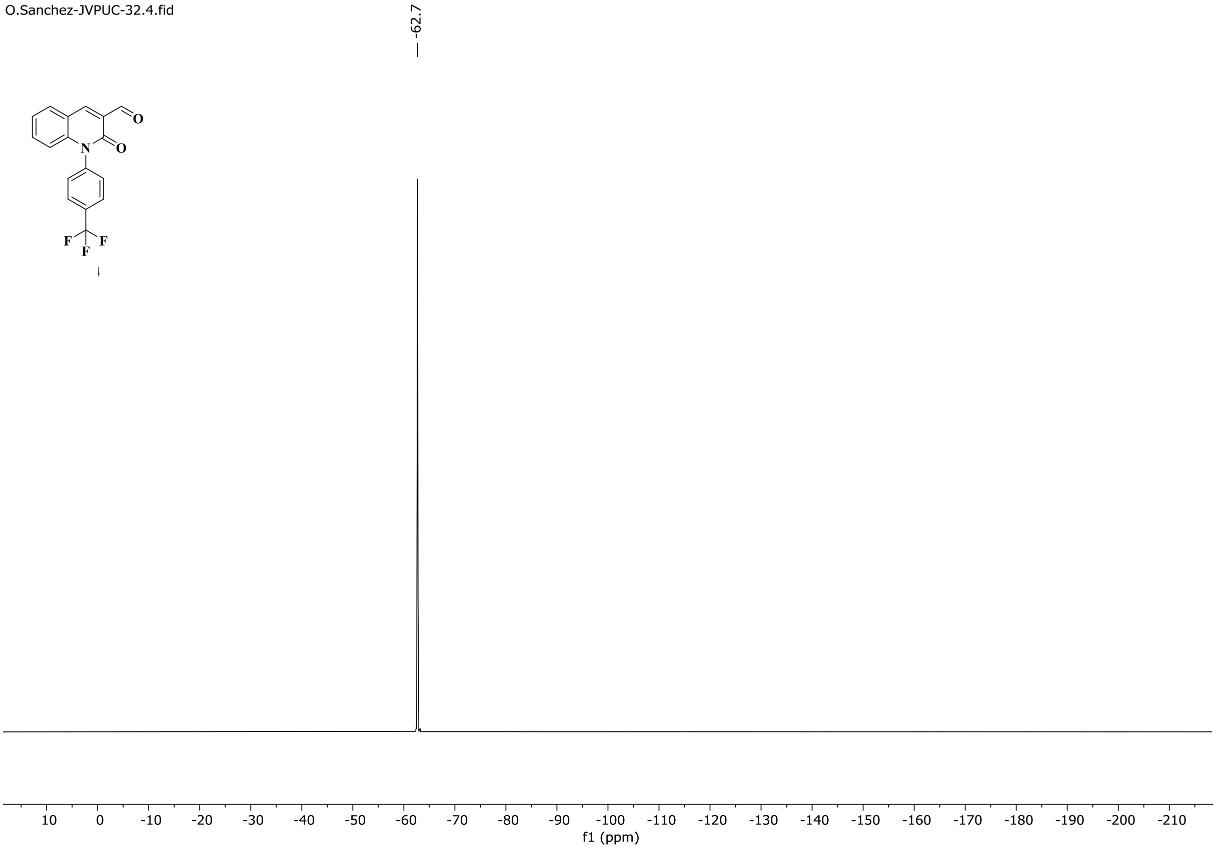

Figure S46. ^{19}F-NMR spectrum of **9t** (DMSO, 376 MHz)

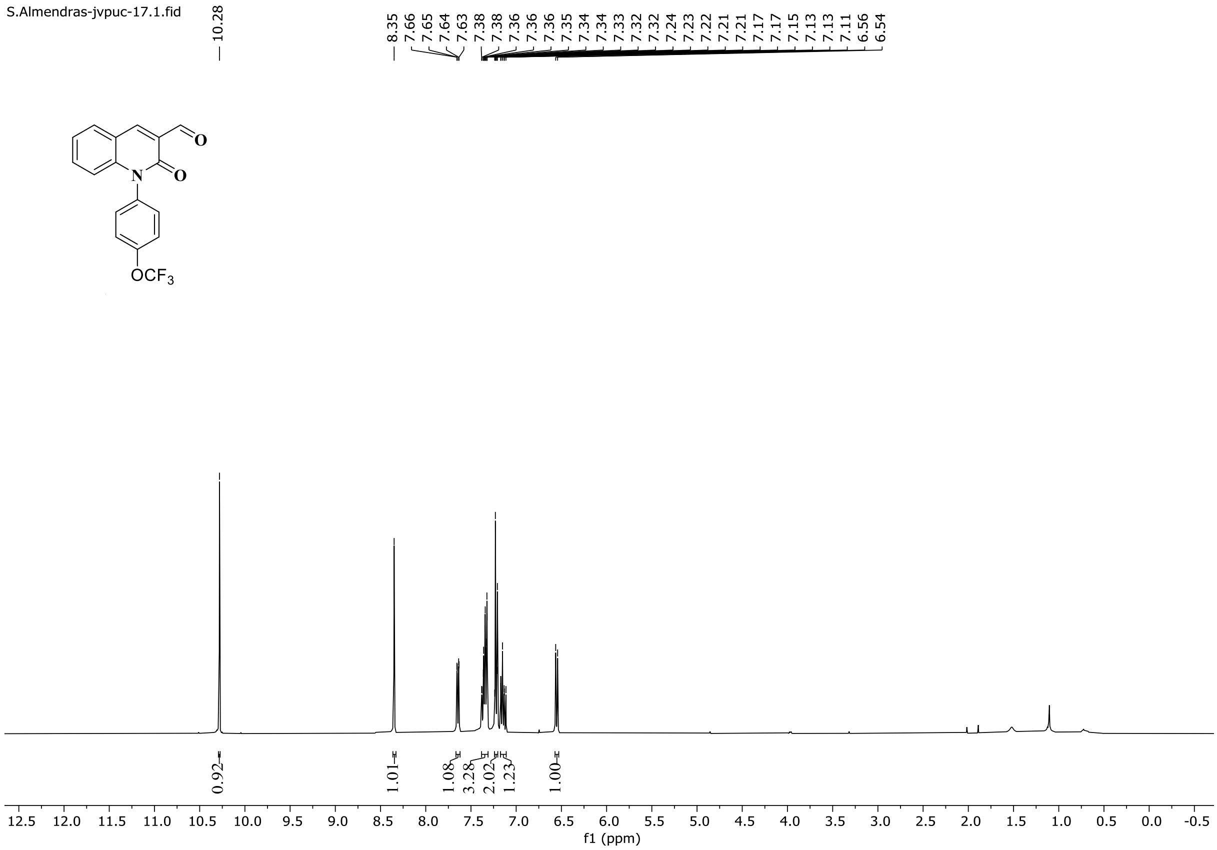

Figure S47. ^{1}H-NMR spectrum of **9u** ($CDCl_3$, 400 MHz)

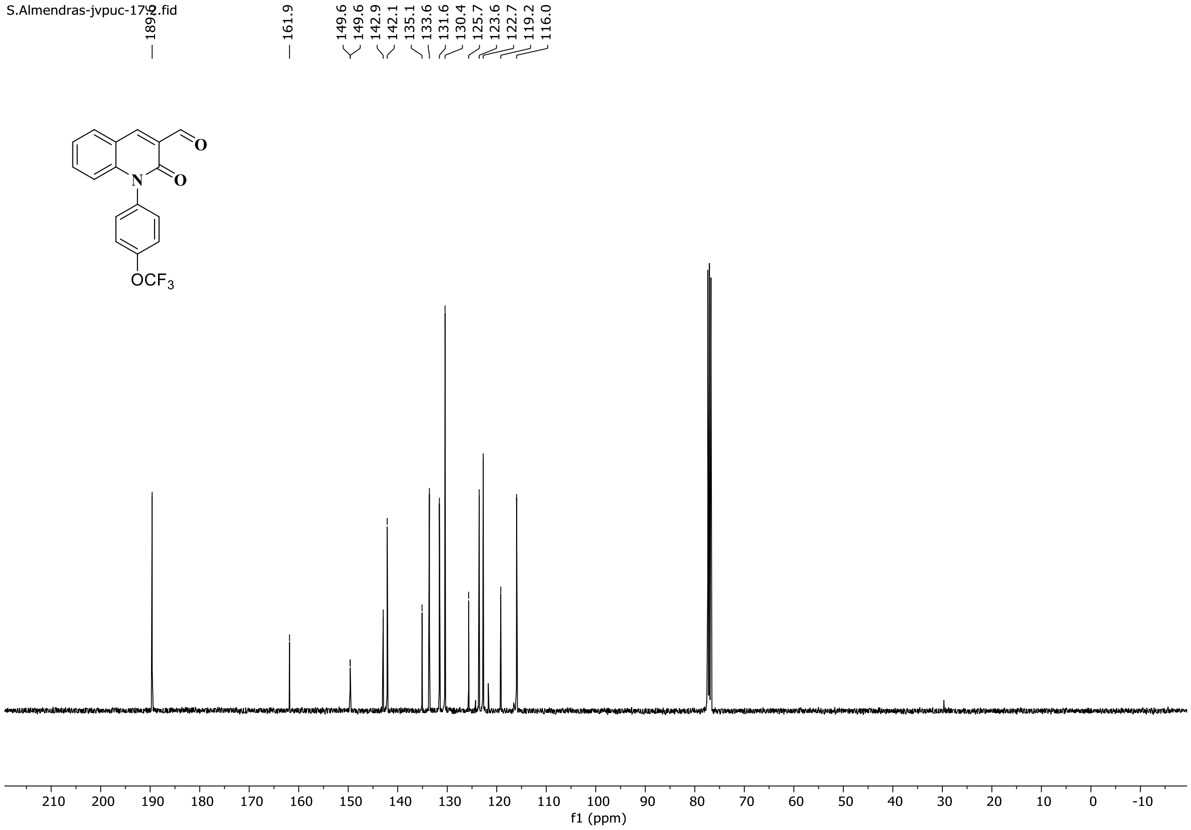

Figure S48. ^{13}C-NMR spectrum of **9u** ($CDCl_3$, 101 MHz)

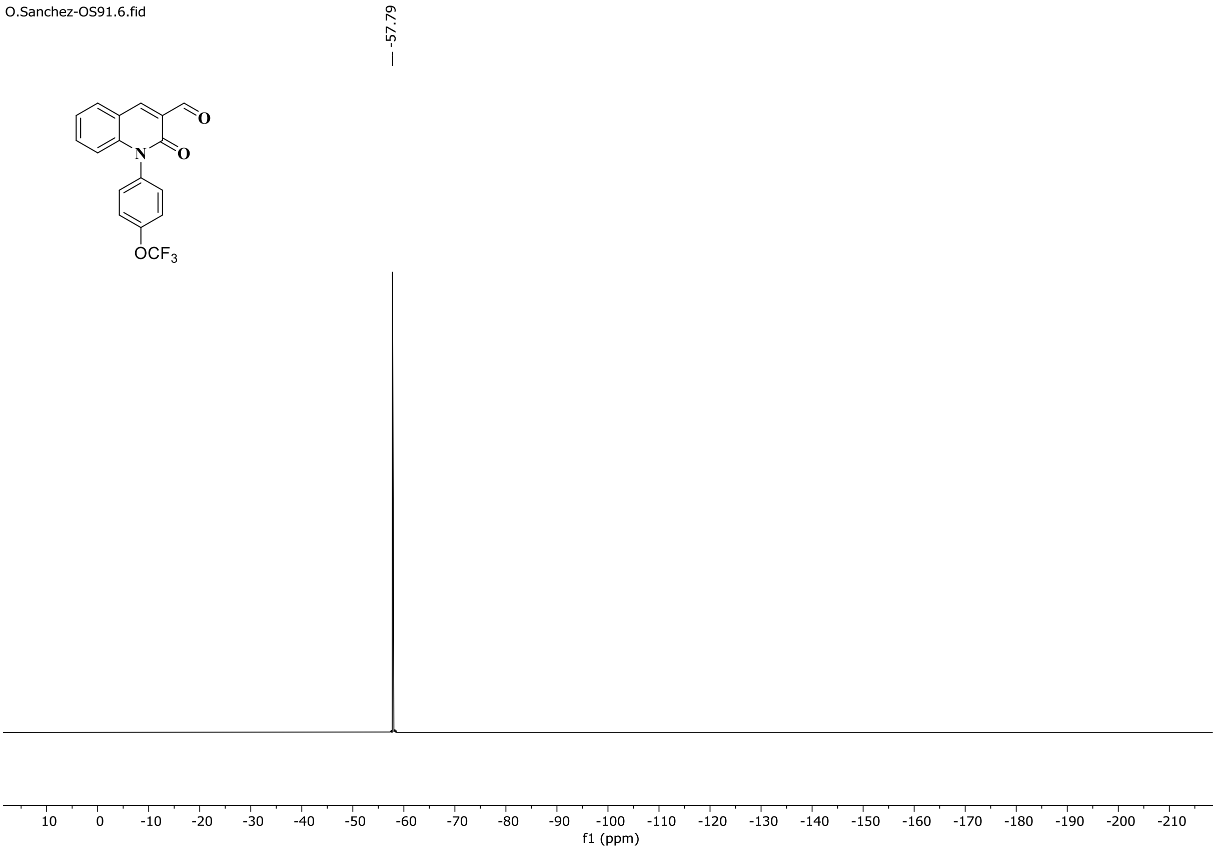

Figure S49. ^{19}F-NMR spectrum of **9u** ($CDCl_3$, 376 MHz)

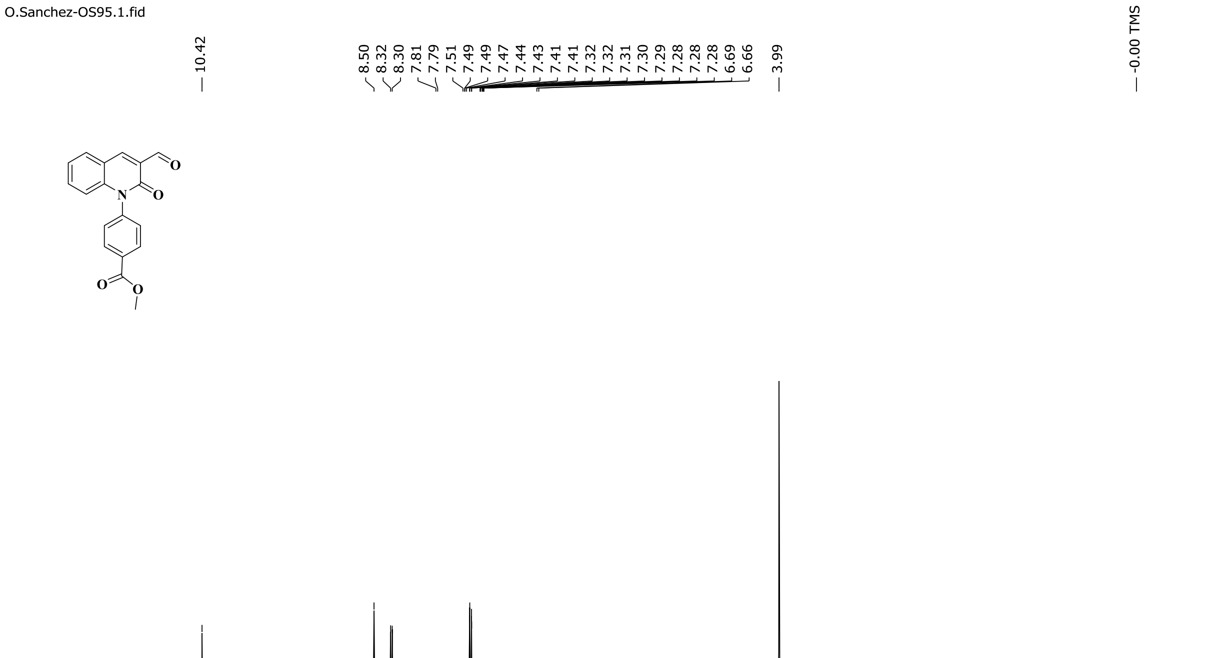

Figure S50. ^{1}H-NMR spectrum of **9v** ($CDCl_3$, 400 MHz)

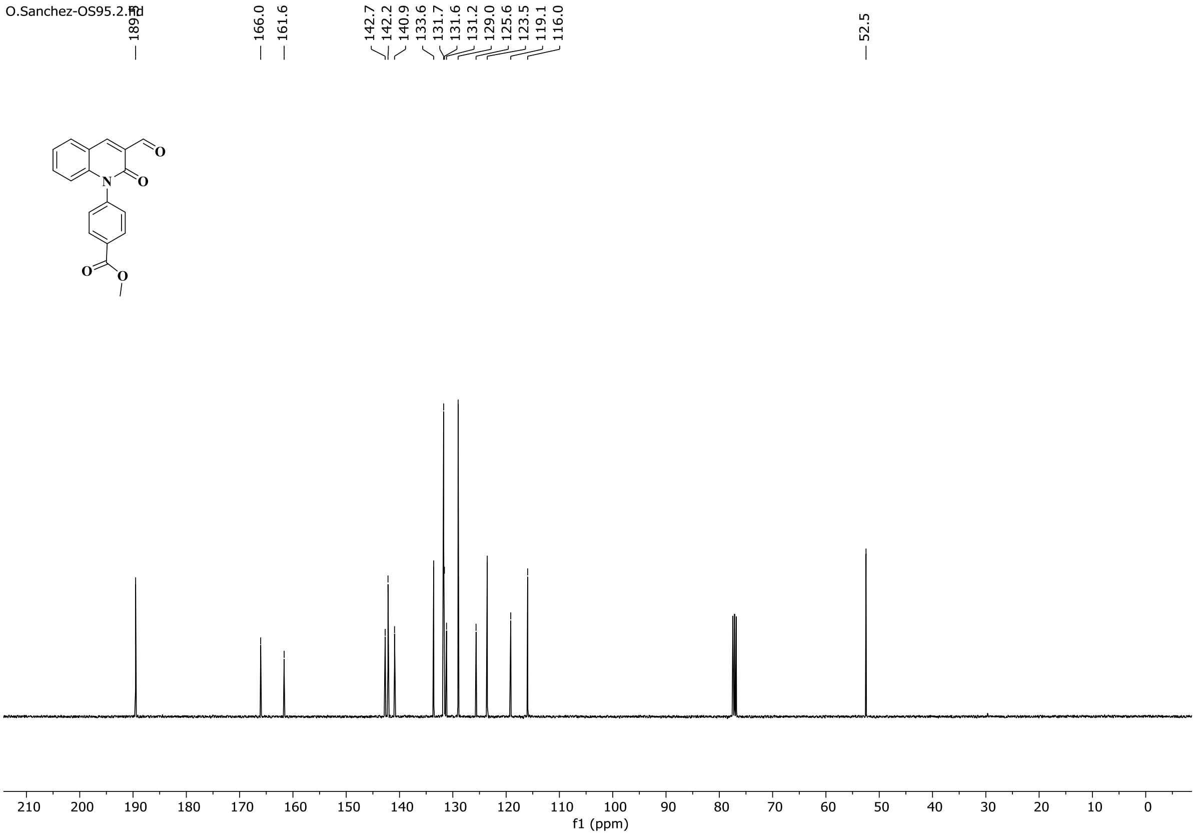

Figure S51. ^{13}C-NMR spectrum of **9v** ($CDCl_3$, 101 MHz)

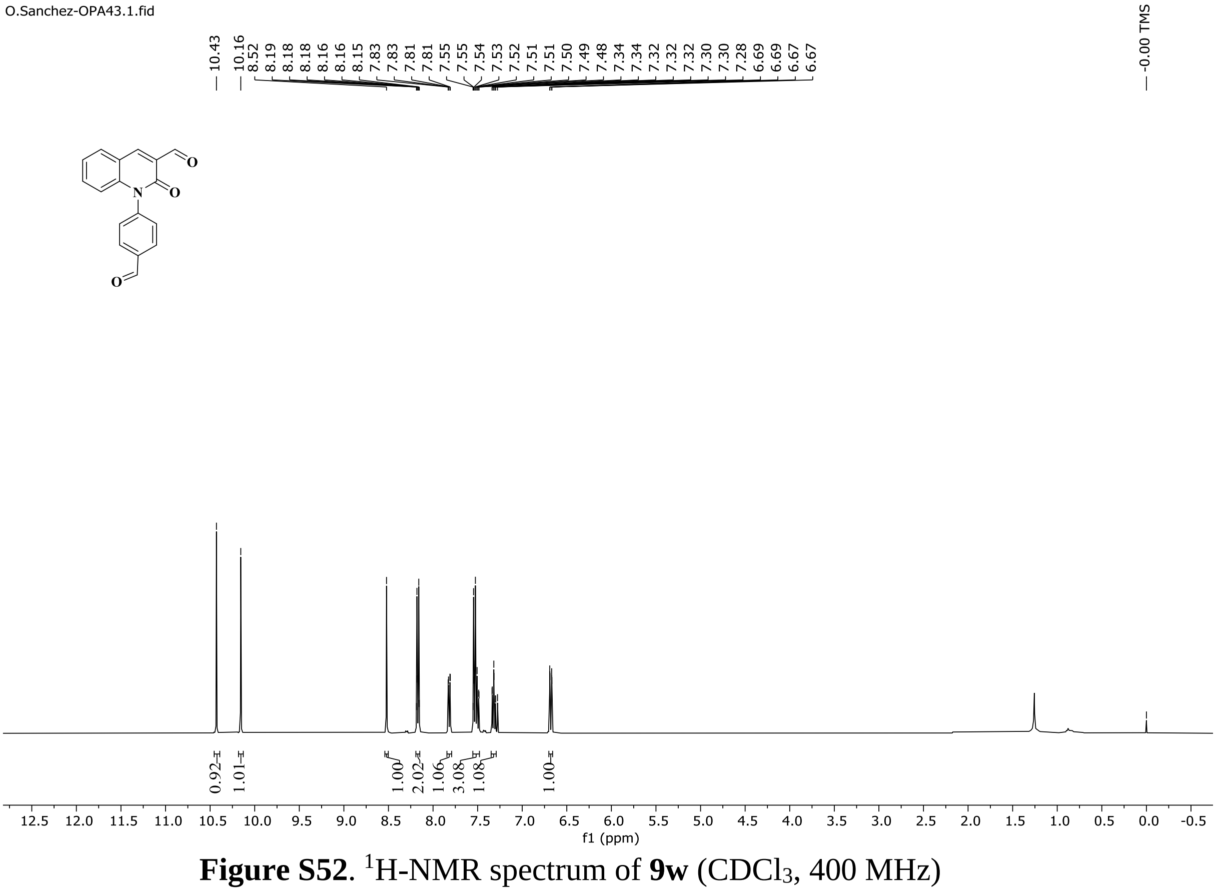

Figure S52. ^{1}H-NMR spectrum of **9w** ($CDCl_3$, 400 MHz)

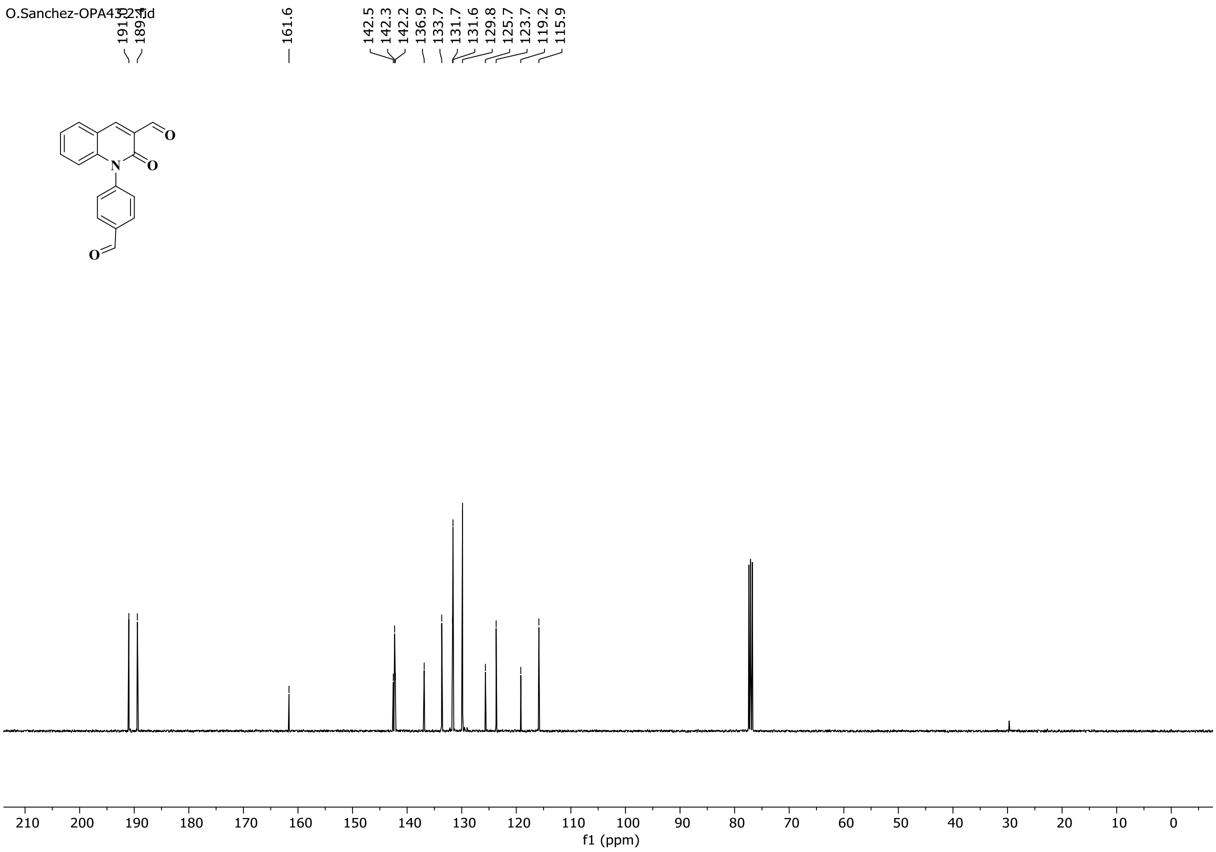

Figure S53. ^{13}C-NMR spectrum of **9w** ($CDCl_3$, 101 MHz)

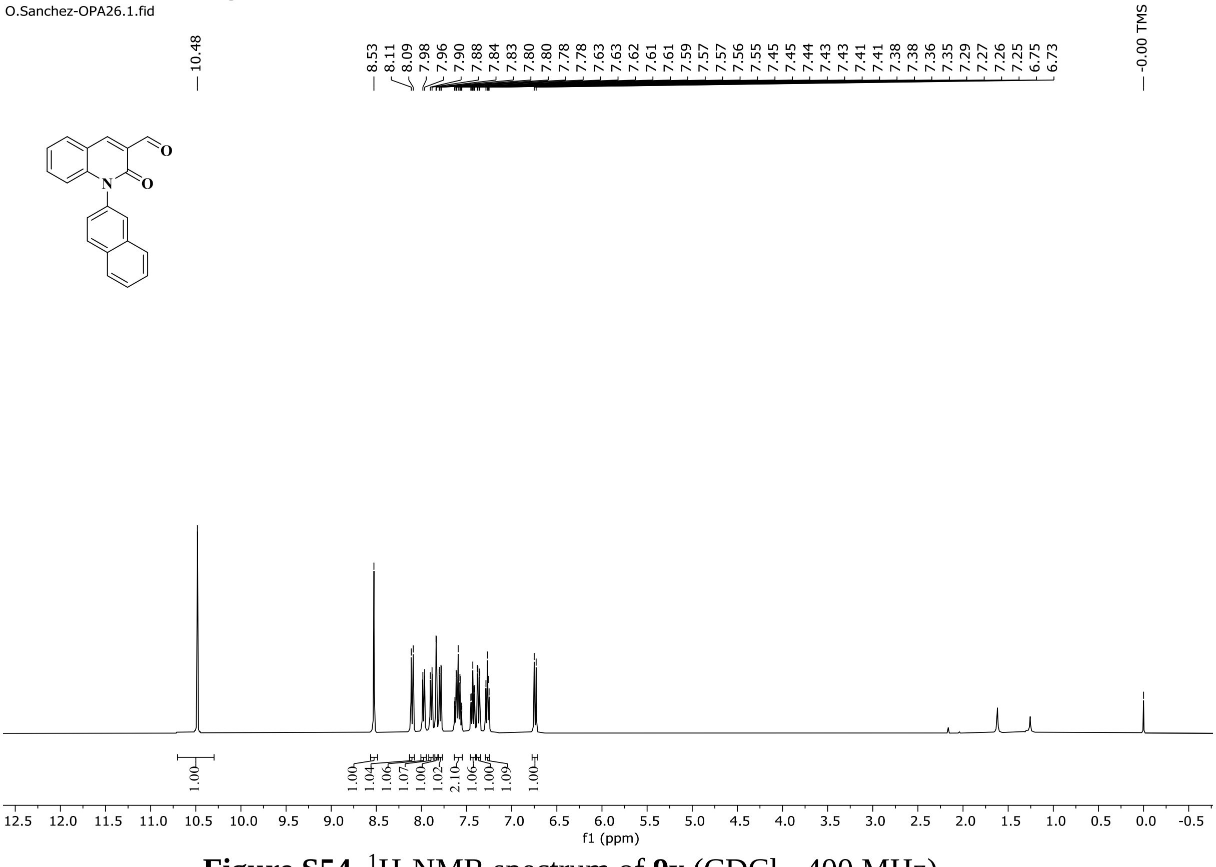

Figure S54. ^{1}H-NMR spectrum of **9x** ($CDCl_3$, 400 MHz)

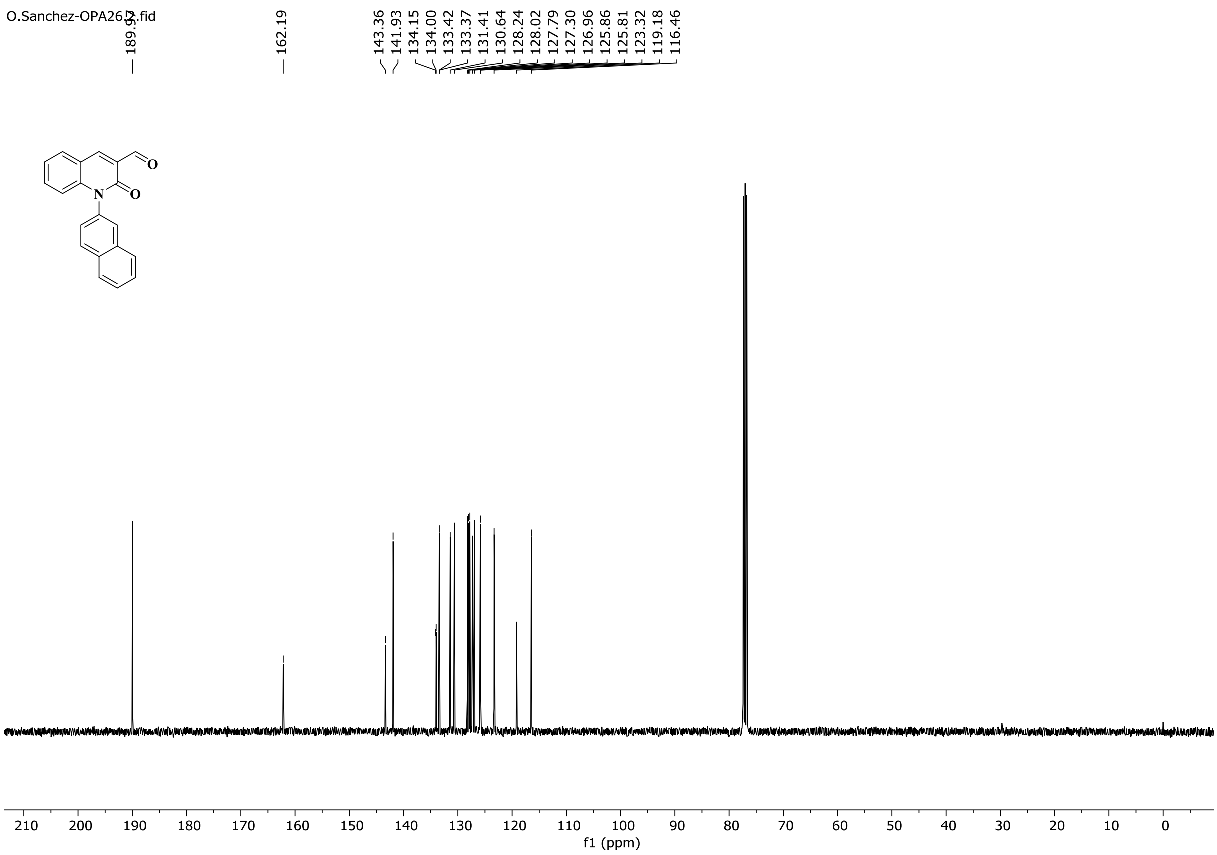

Figure S55. ^{13}C-NMR spectrum of **9x** ($CDCl_3$, 101 MHz)

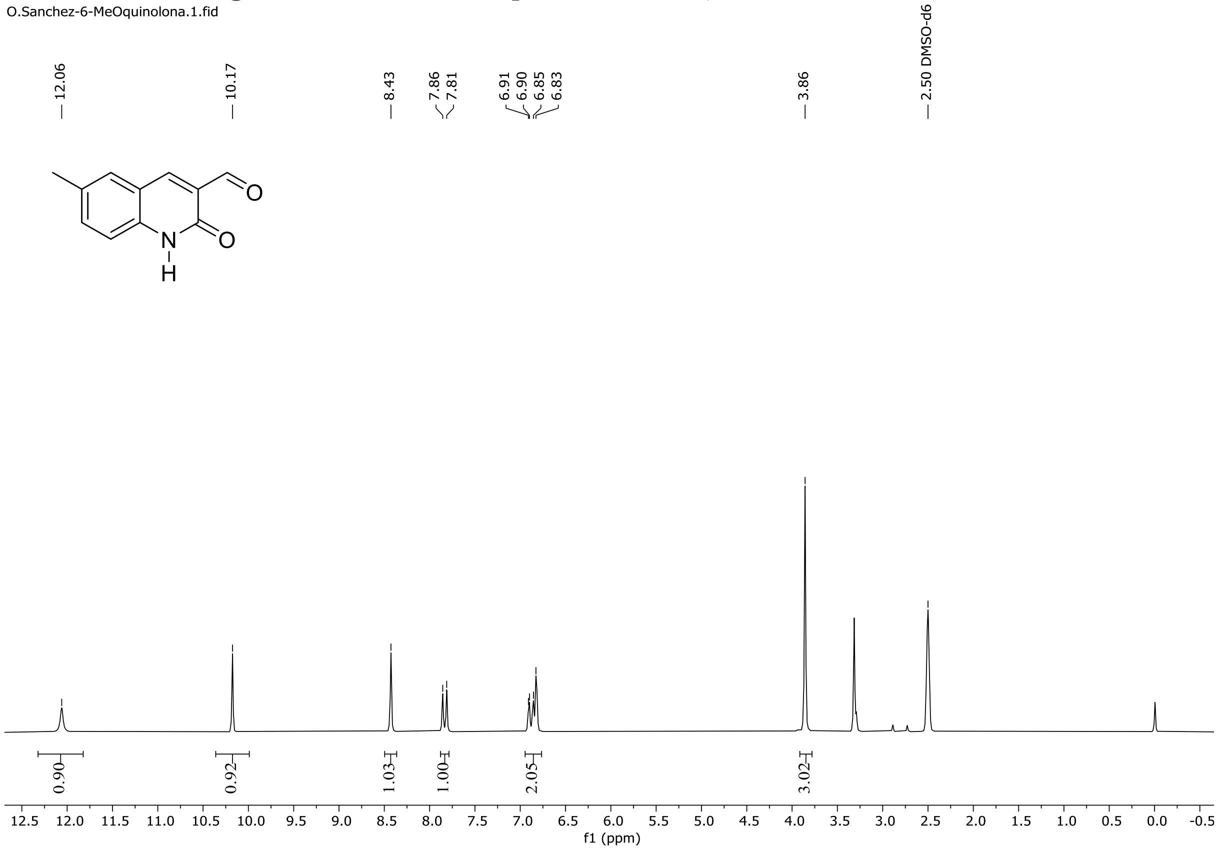

Figure S56. ^{1}H-NMR spectrum of **7b** (DMSO, 200 MHz)

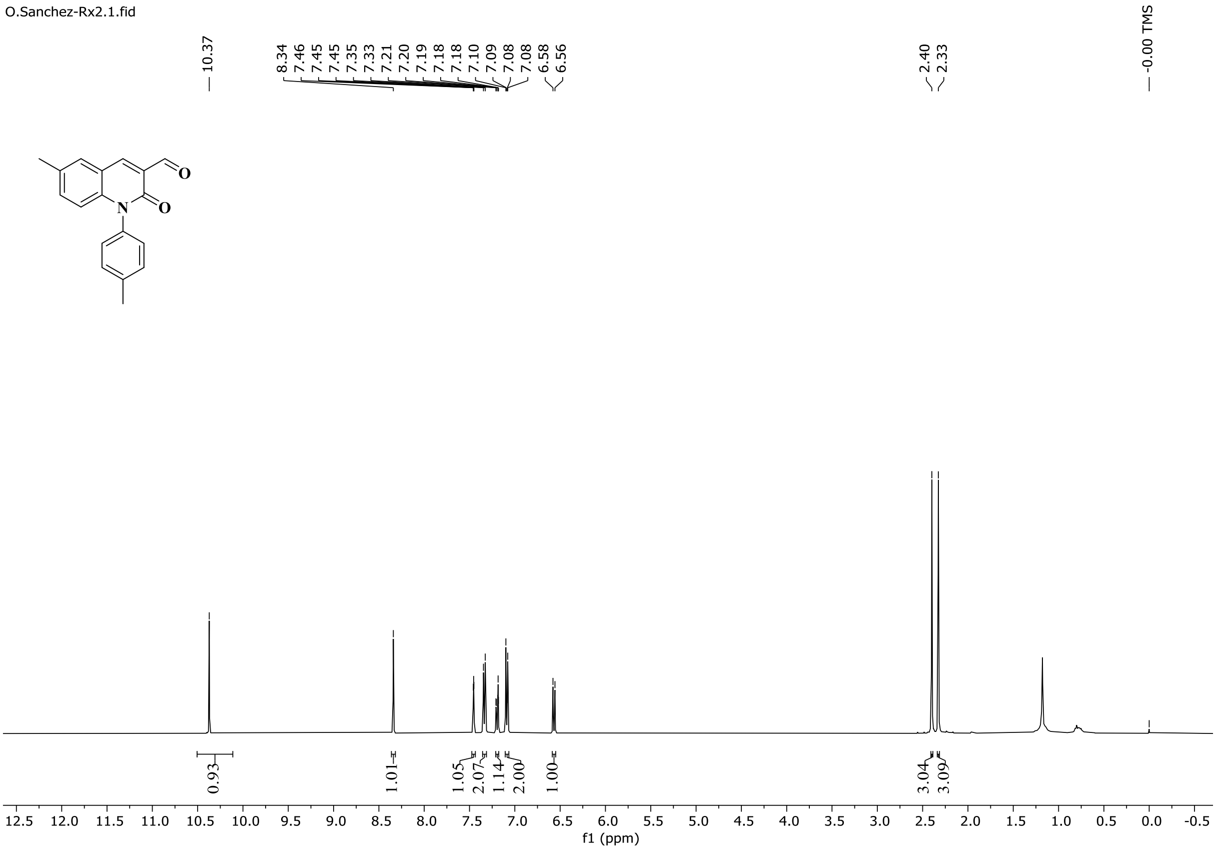

Figure S57. ^{1}H-NMR spectrum of **10a** ($CDCl_3$, 400 MHz)

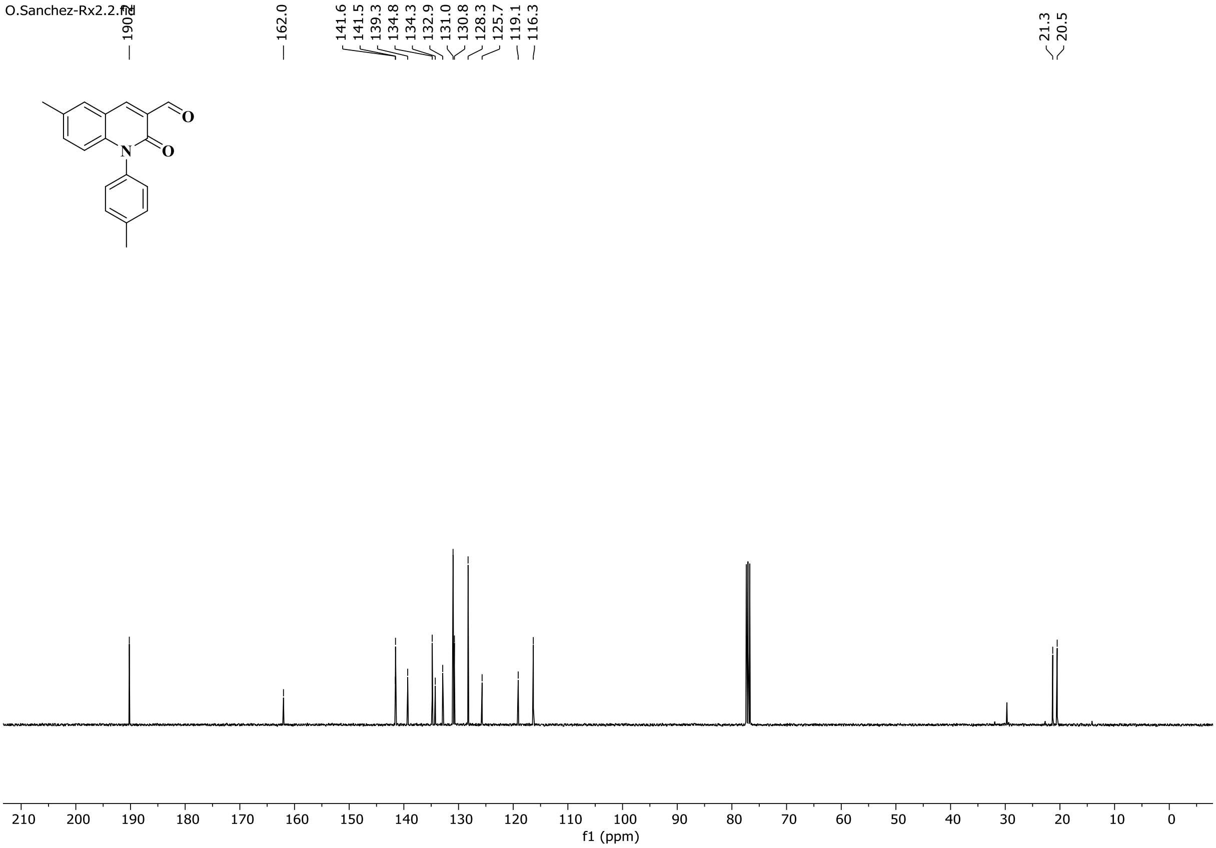

Figure S58. ^{13}C-NMR spectrum of **10a** ($CDCl_3$, 101 MHz)

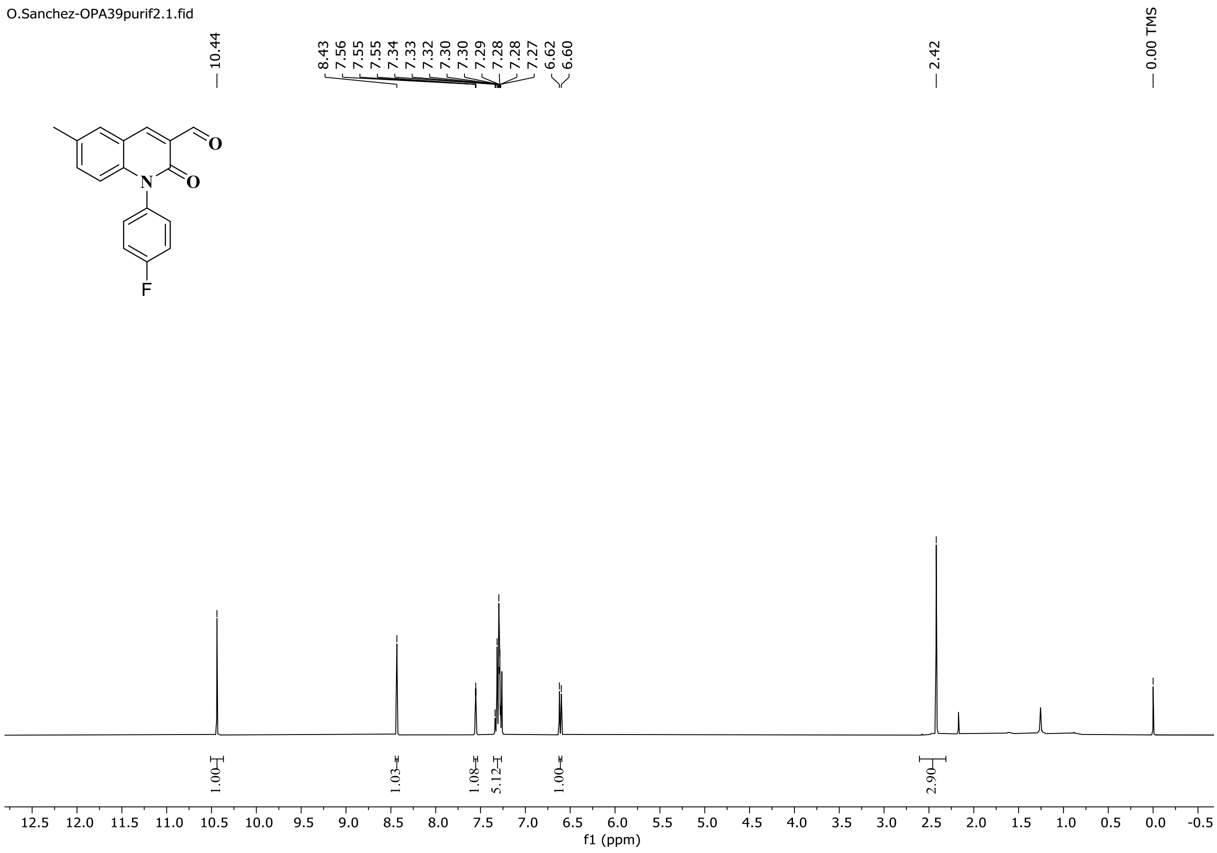

Figure S59. ^{1}H-NMR spectrum of **10b** ($CDCl_3$, 400 MHz)

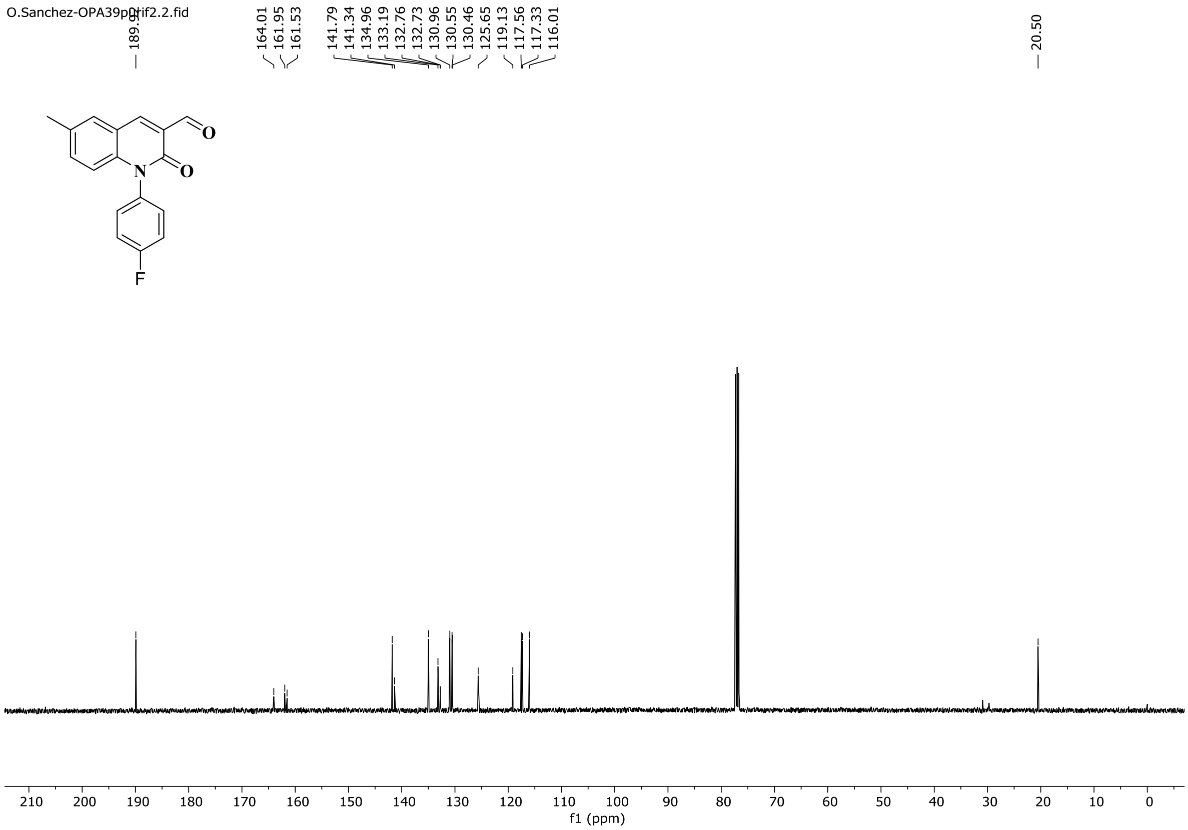

Figure S60. ^{13}C-NMR spectrum of **10b** ($CDCl_3$, 101 MHz)

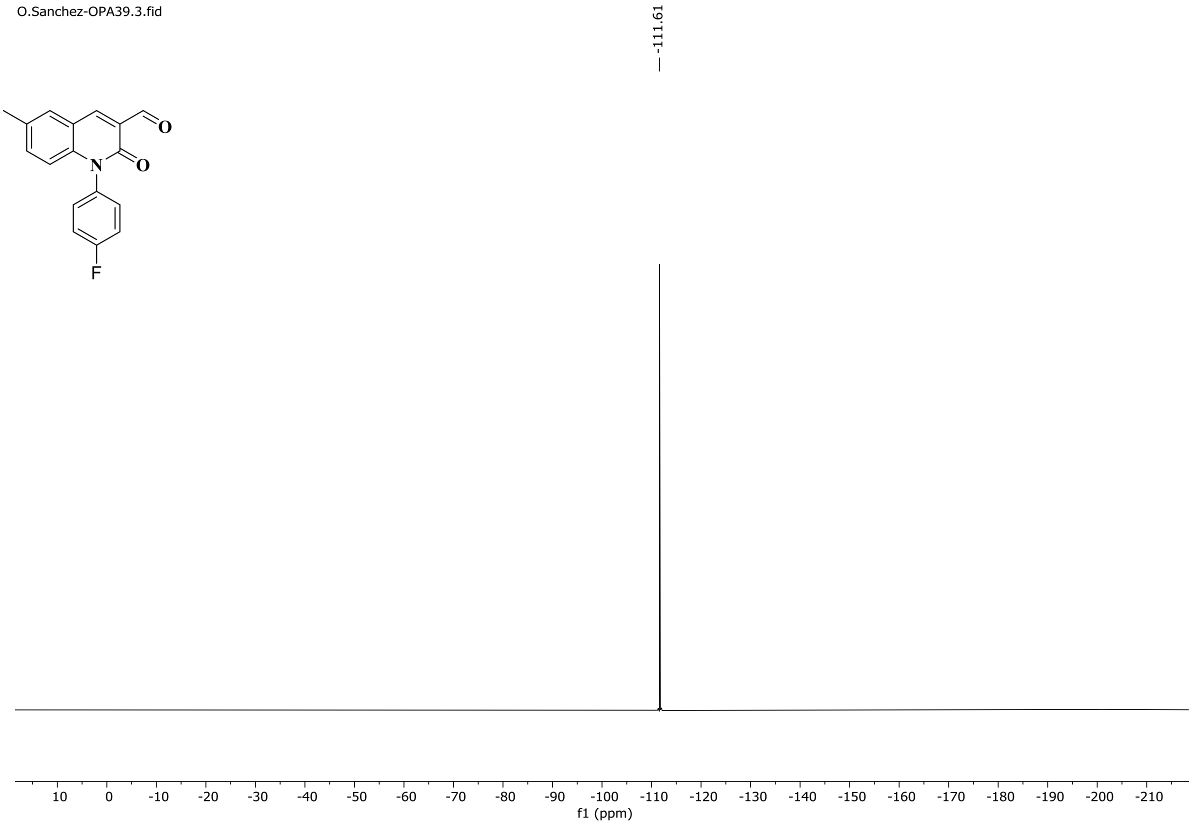

Figure S61. ^{19}F-NMR spectrum of **10b** ($CDCl_3$, 376 MHz)

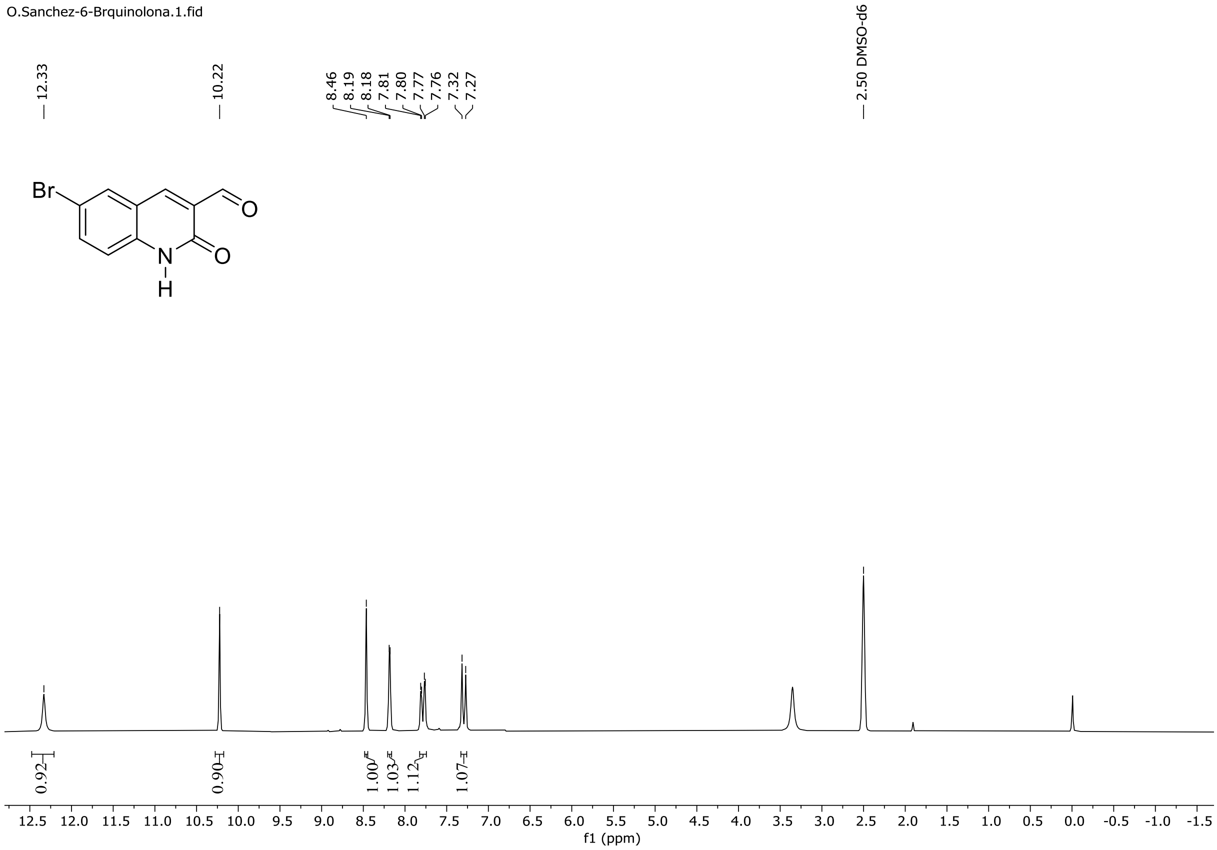

Figure S62. ^{1}H-NMR spectrum of **7c** (DMSO, 200 MHz)

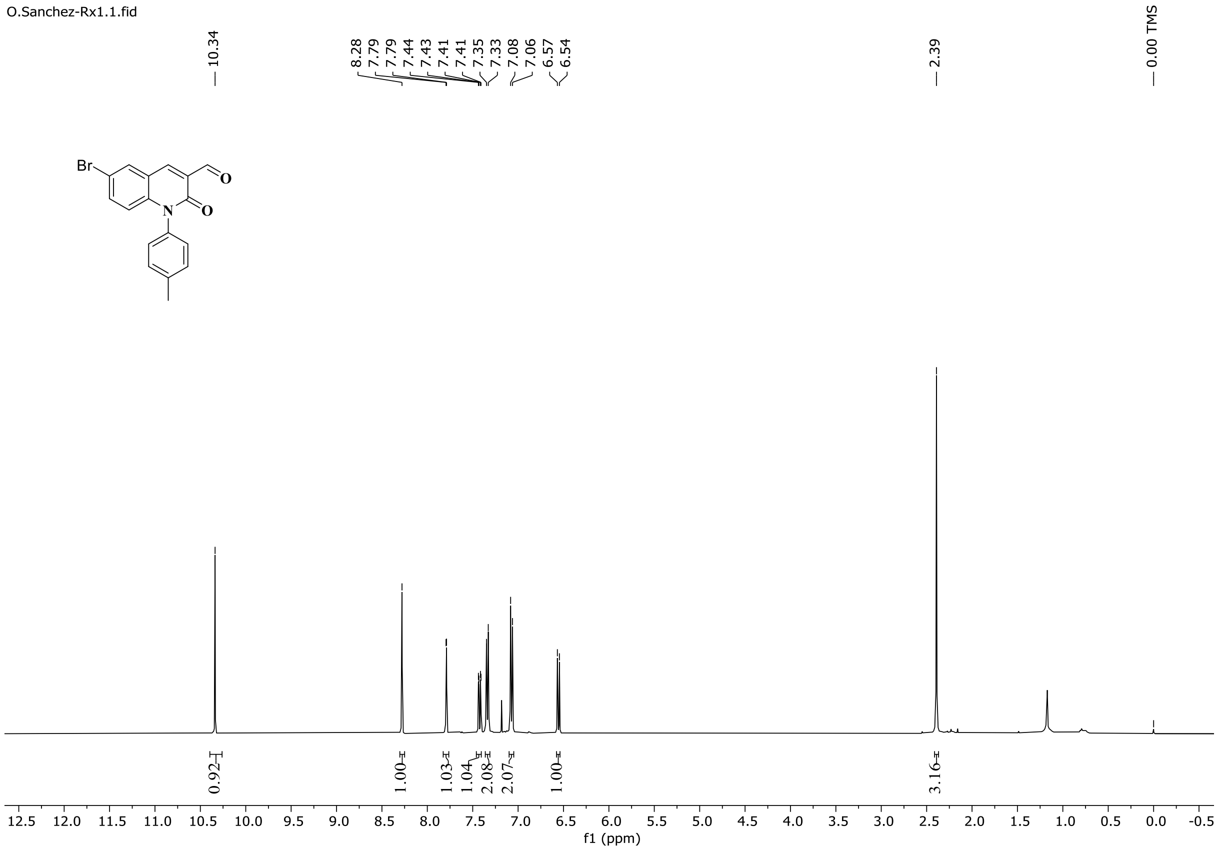

Figure S63. ^{1}H-NMR spectrum of **10c** ($CDCl_3$, 400 MHz)

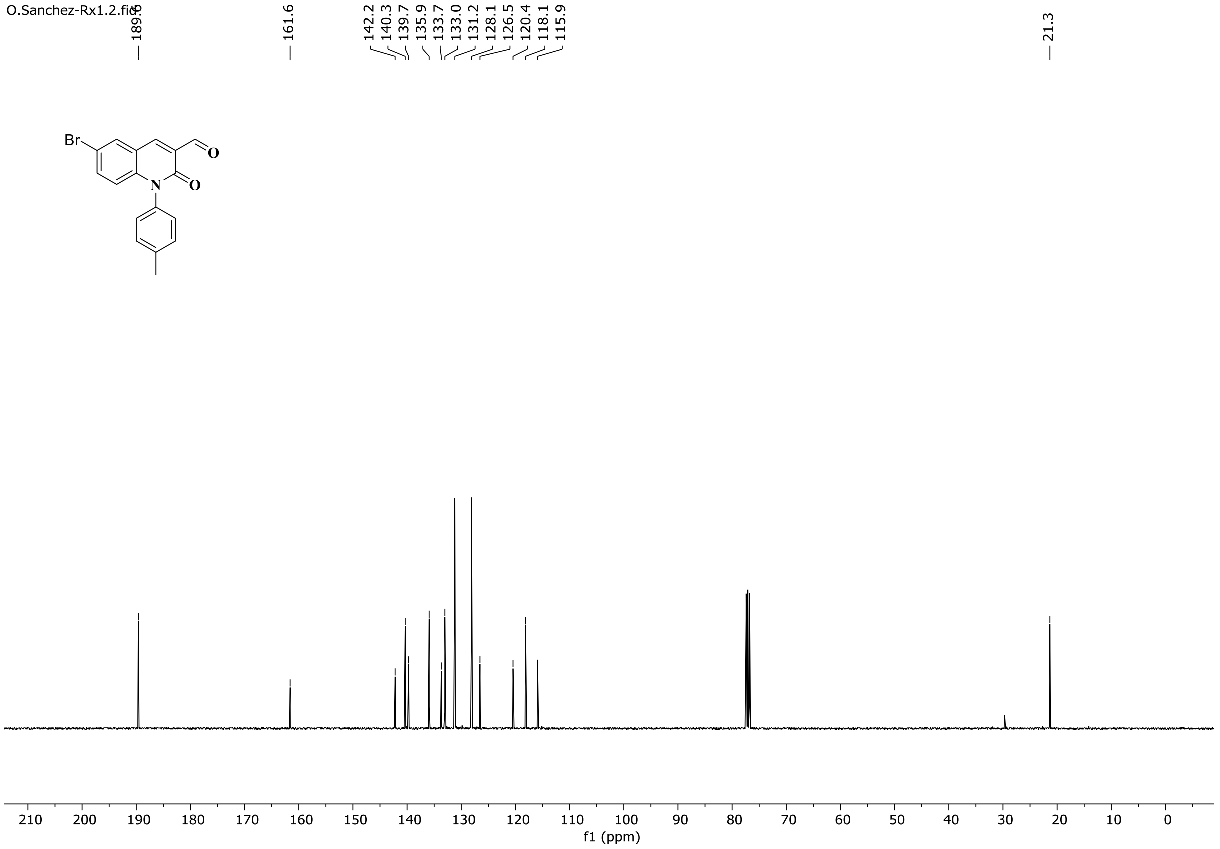

Figure S64. ^{13}C-NMR spectrum of **10c** ($CDCl_3$, 101 MHz)

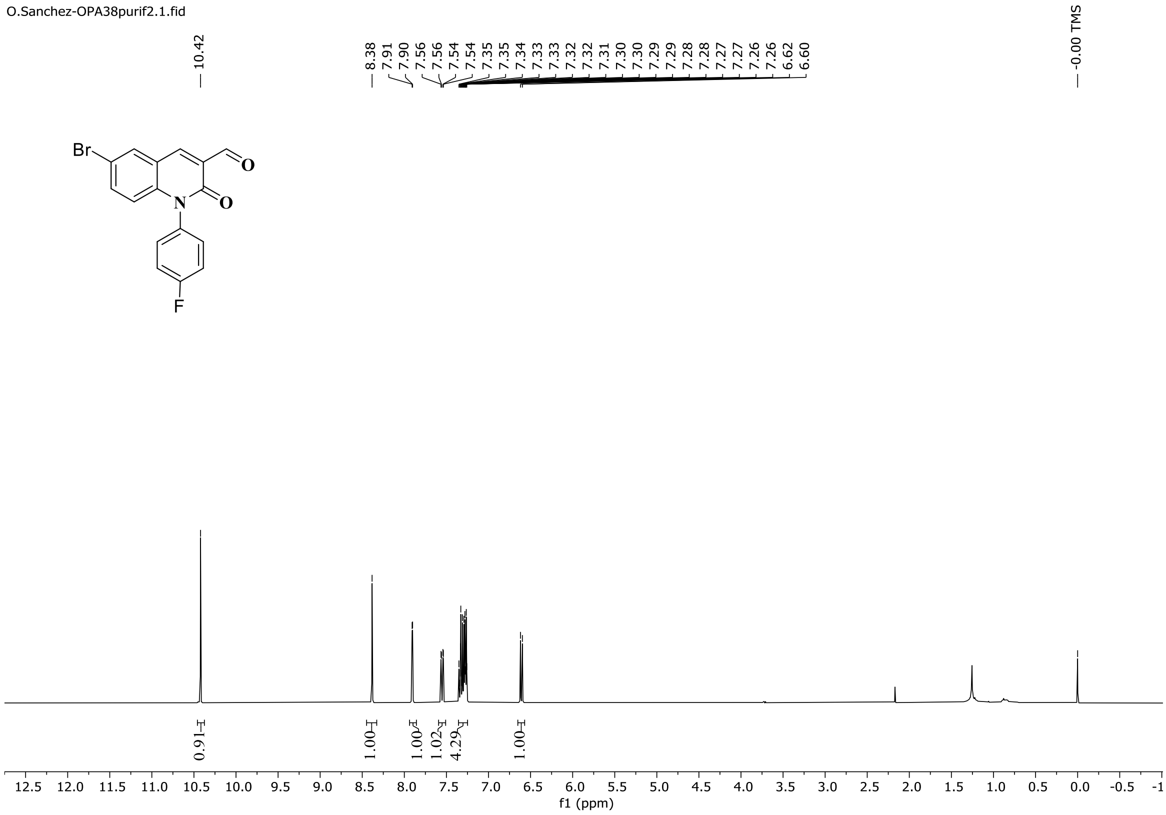

Figure S65. ^{1}H-NMR spectrum of **10d** ($CDCl_3$, 400 MHz)

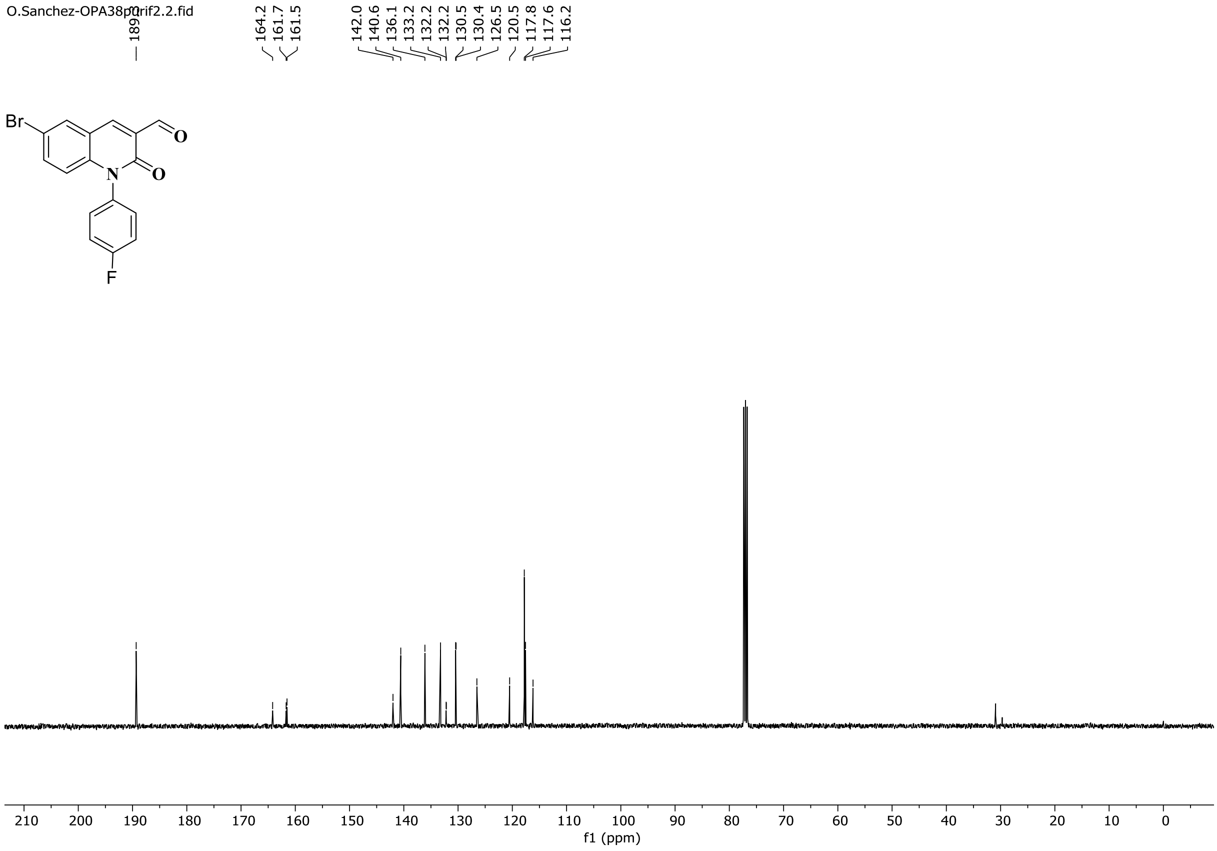

Figure S66. ^{13}C-NMR spectrum of **10d** ($CDCl_3$, 101 MHz)

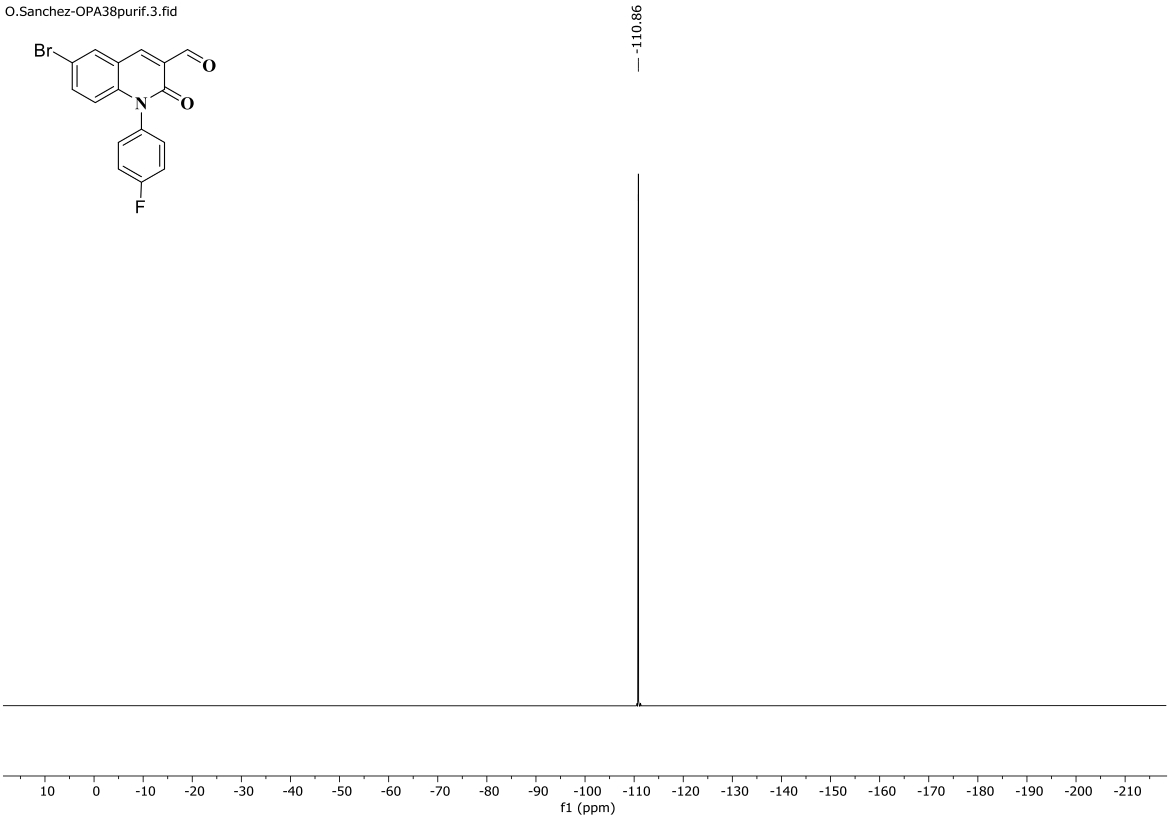

Figure S67. ^{19}F-NMR spectrum of **10d** ($CDCl_3$, 376 MHz)

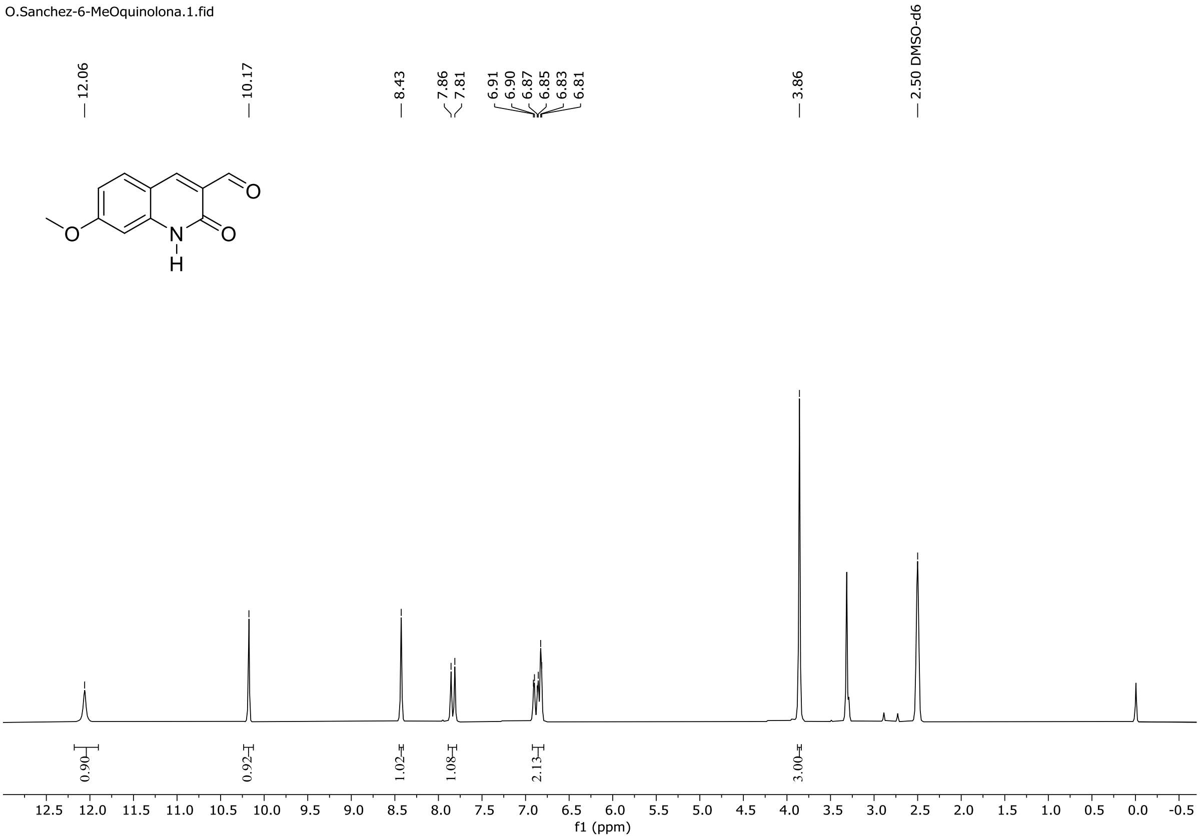

Figure S68. ^{1}H-NMR spectrum of **7d** (DMSO, 200 MHz)

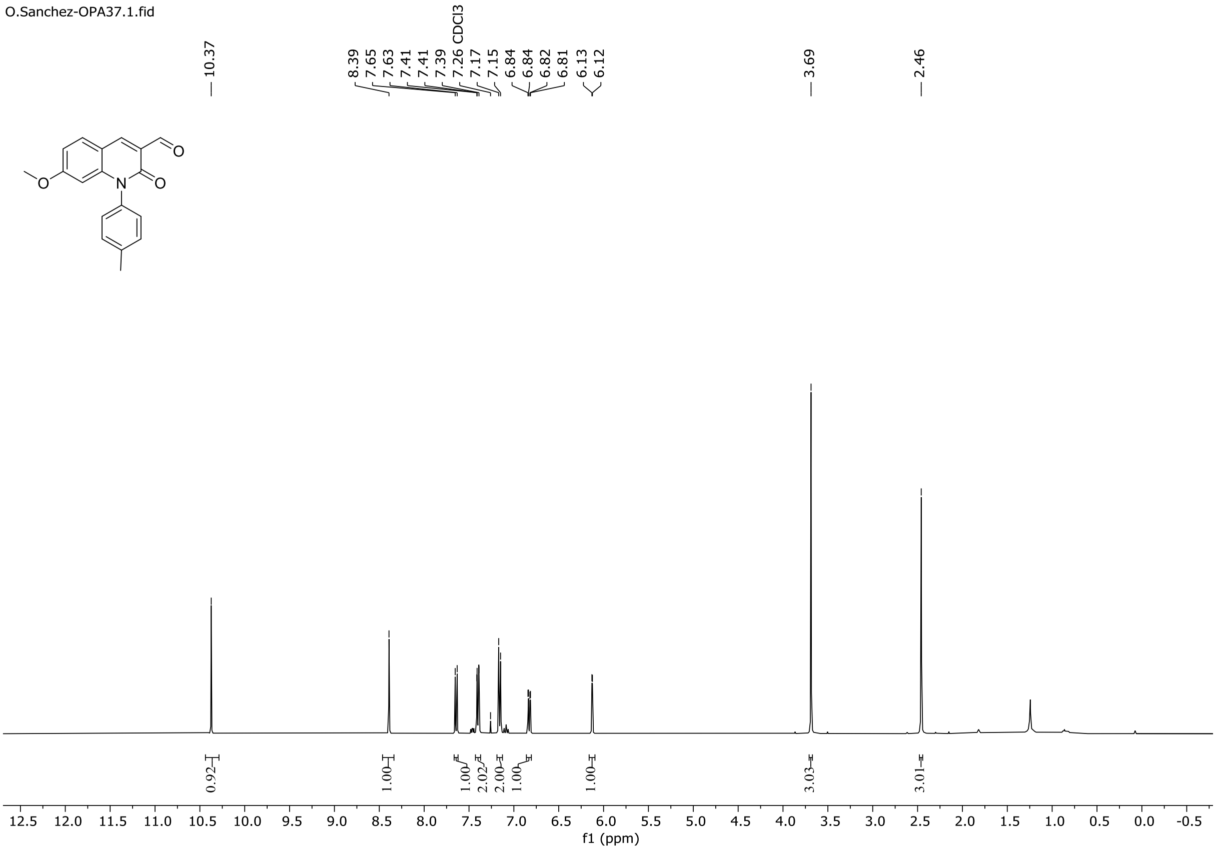

Figure S69. ^{1}H-NMR spectrum of **10e** ($CDCl_3$, 400 MHz)

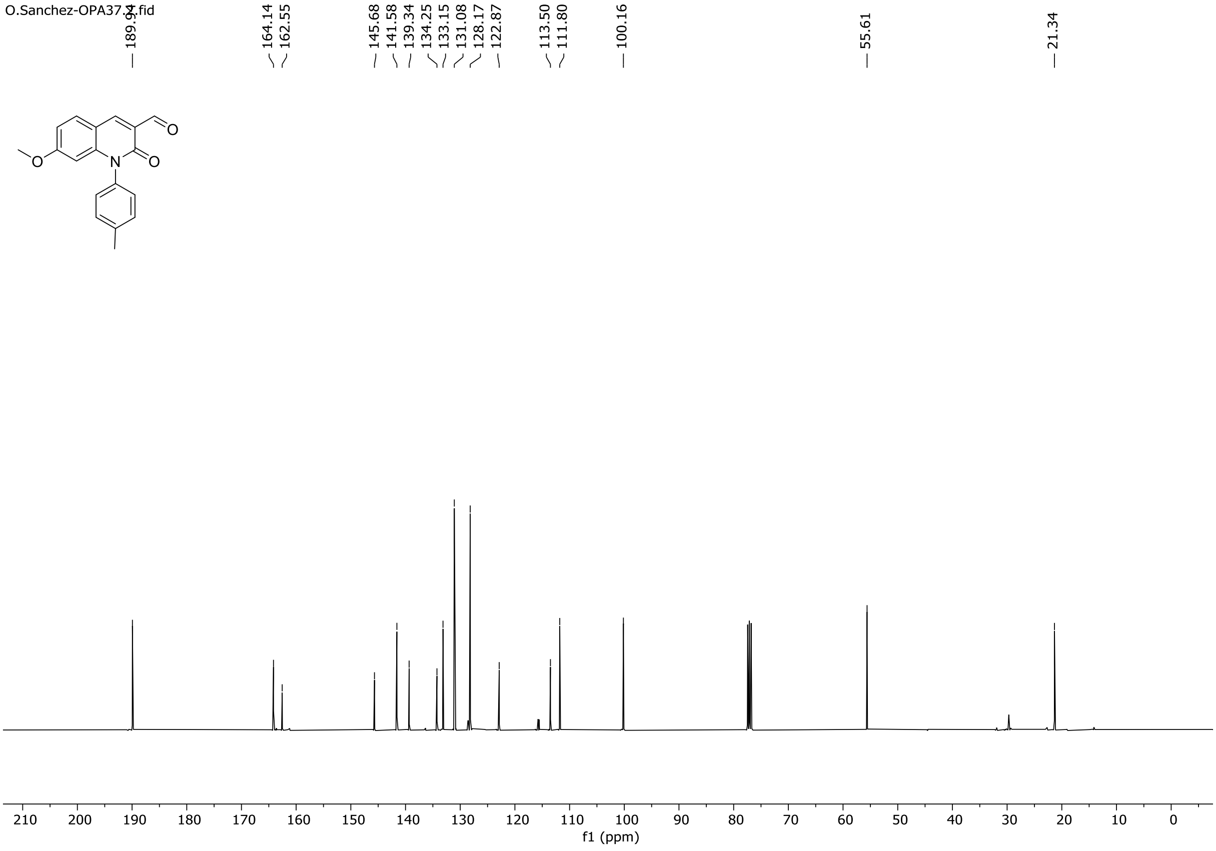

Figure S70. ^{13}C-NMR spectrum of **10e** ($CDCl_3$, 101 MHz)

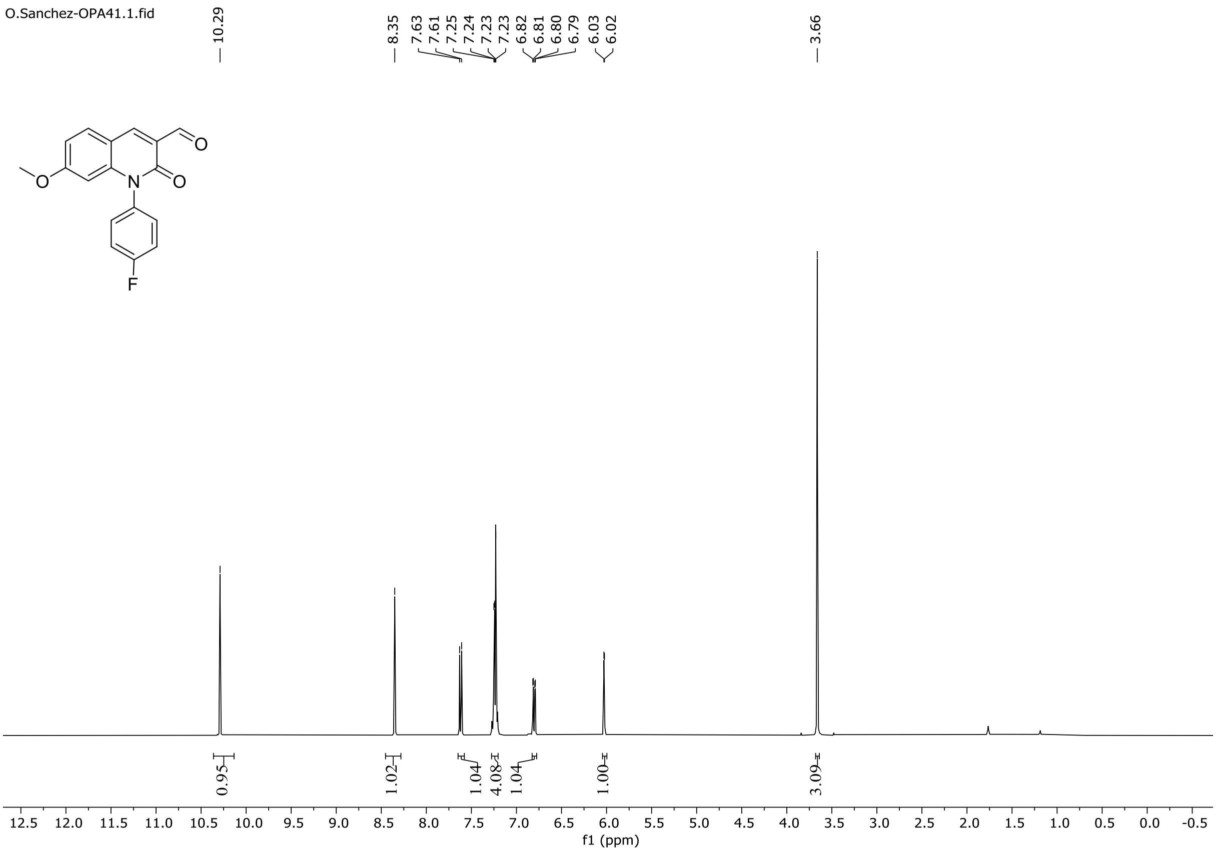

Figure S71. ^{1}H-NMR spectrum of **10h** ($CDCl_3$, 400 MHz)

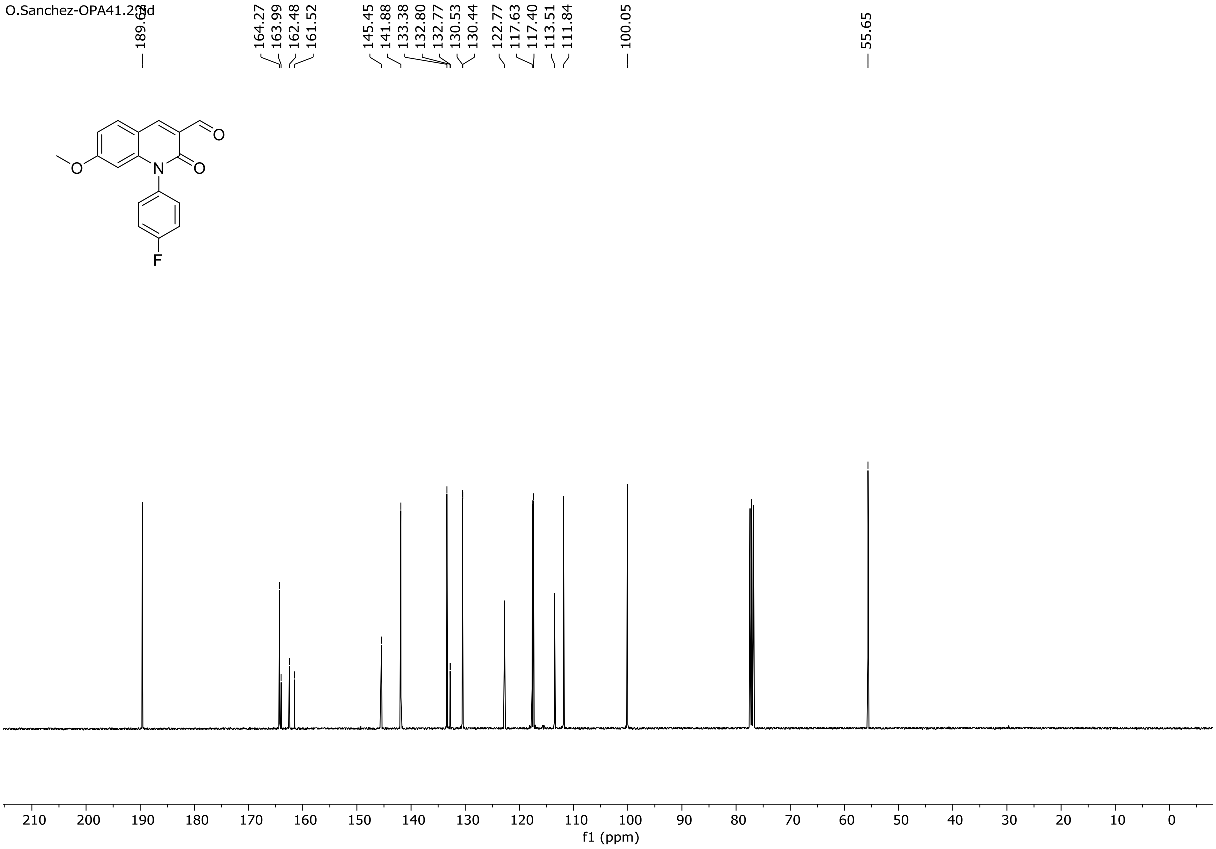

Figure S72. ^{13}C-NMR spectrum of **10h** ($CDCl_3$, 101 MHz)

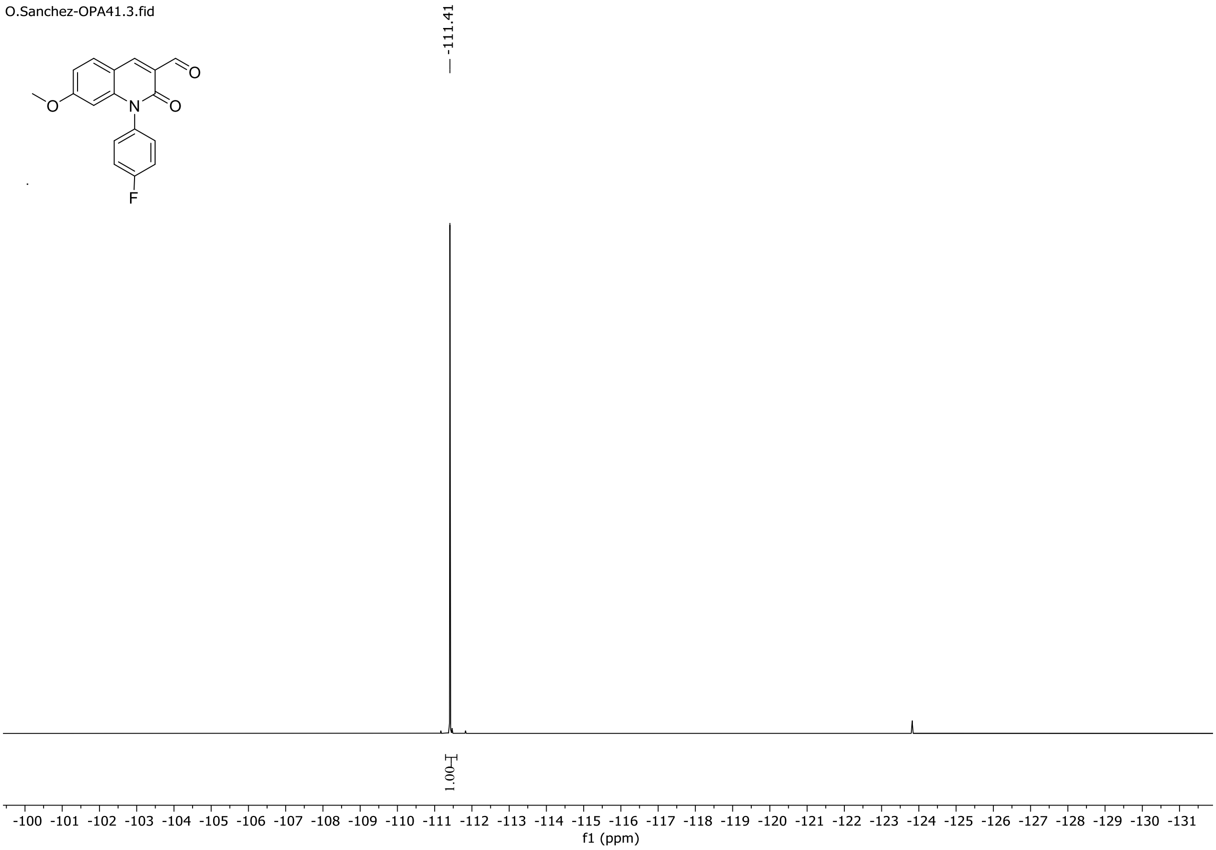

Figure S73. ^{19}F-NMR spectrum of **10h** ($CDCl_3$, 376 MHz)

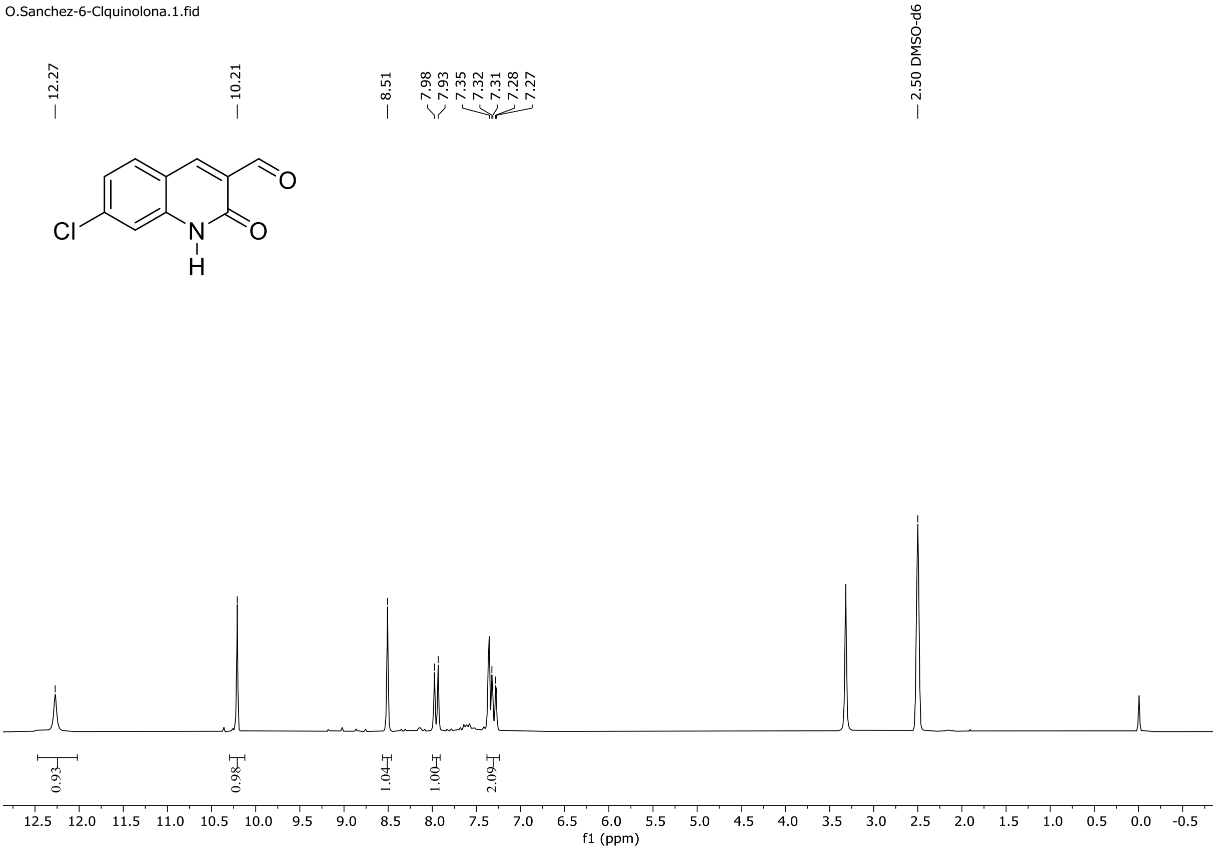

Figure S74. ^{1}H-NMR spectrum of **7e** (DMSO, 200 MHz)

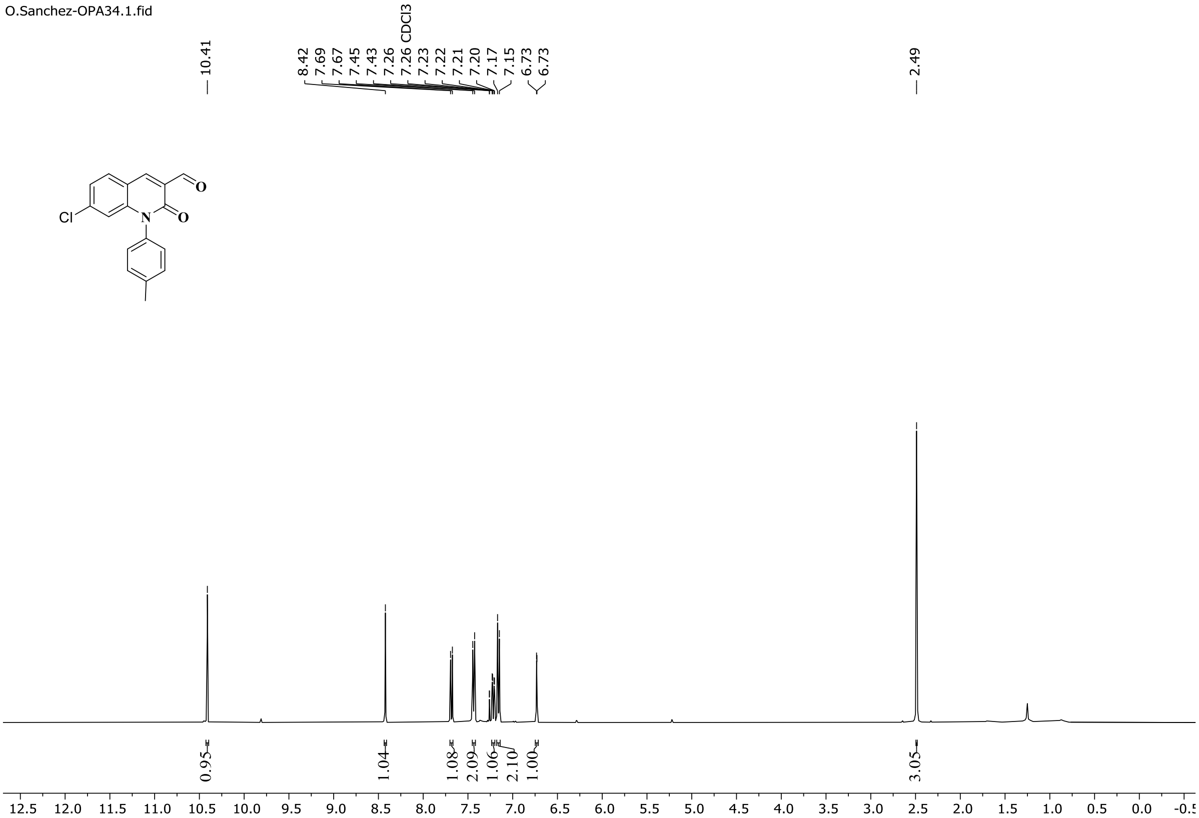

Figure S75. ^{1}H-NMR spectrum of **10g** ($CDCl_3$, 400 MHz)

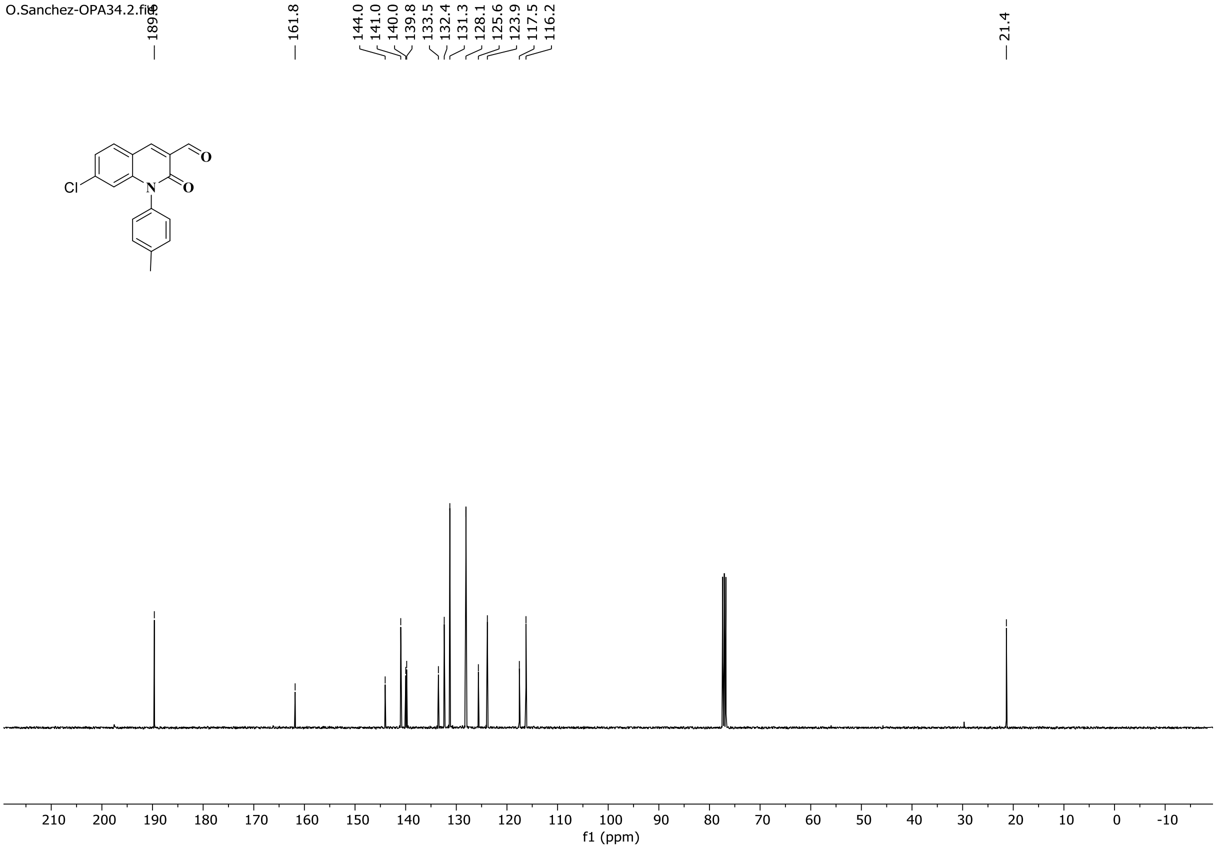

Figure S76. ^{13}C-NMR spectrum of **10g** ($CDCl_3$, 101 MHz)

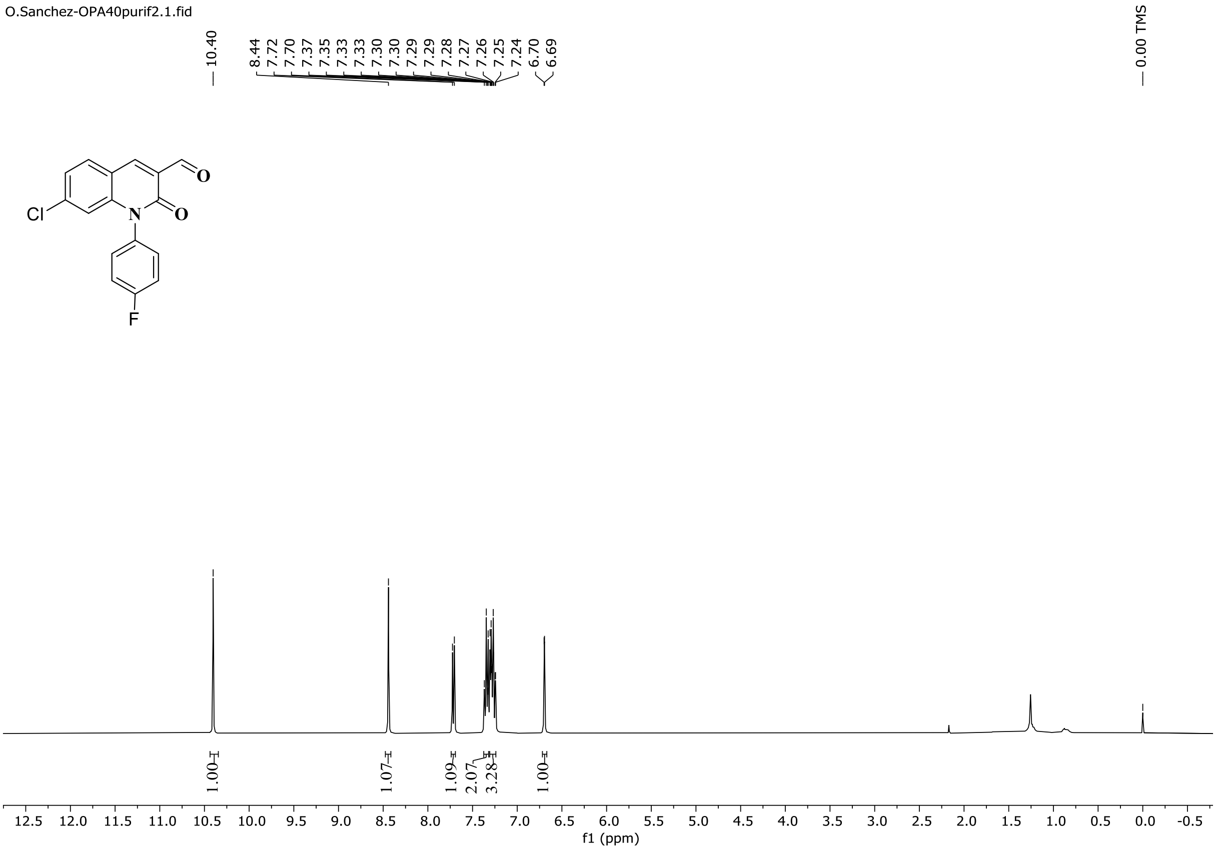

Figure S77. ^{1}H-NMR spectrum of **10h** ($CDCl_3$, 400 MHz)

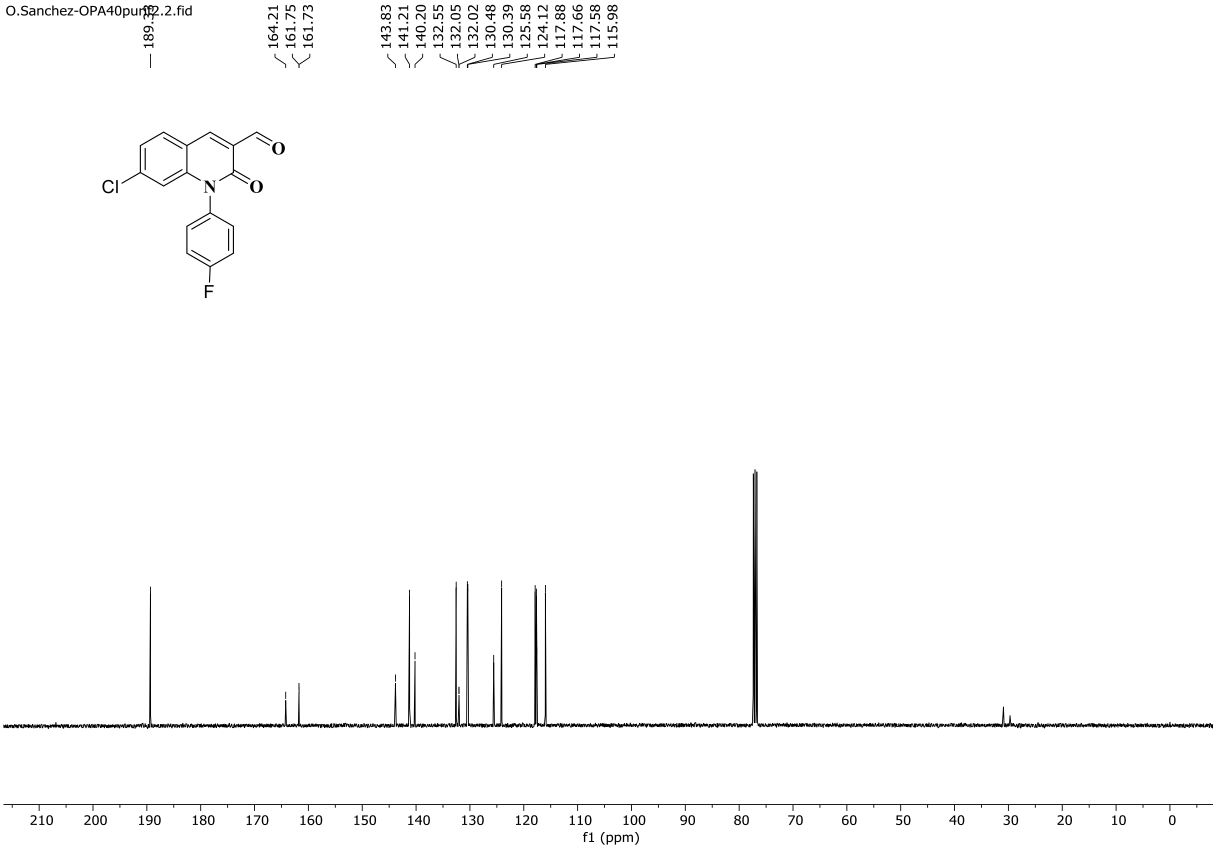

Figure S78. ^{13}C-NMR spectrum of **10h**($CDCl_3$, 101 MHz)

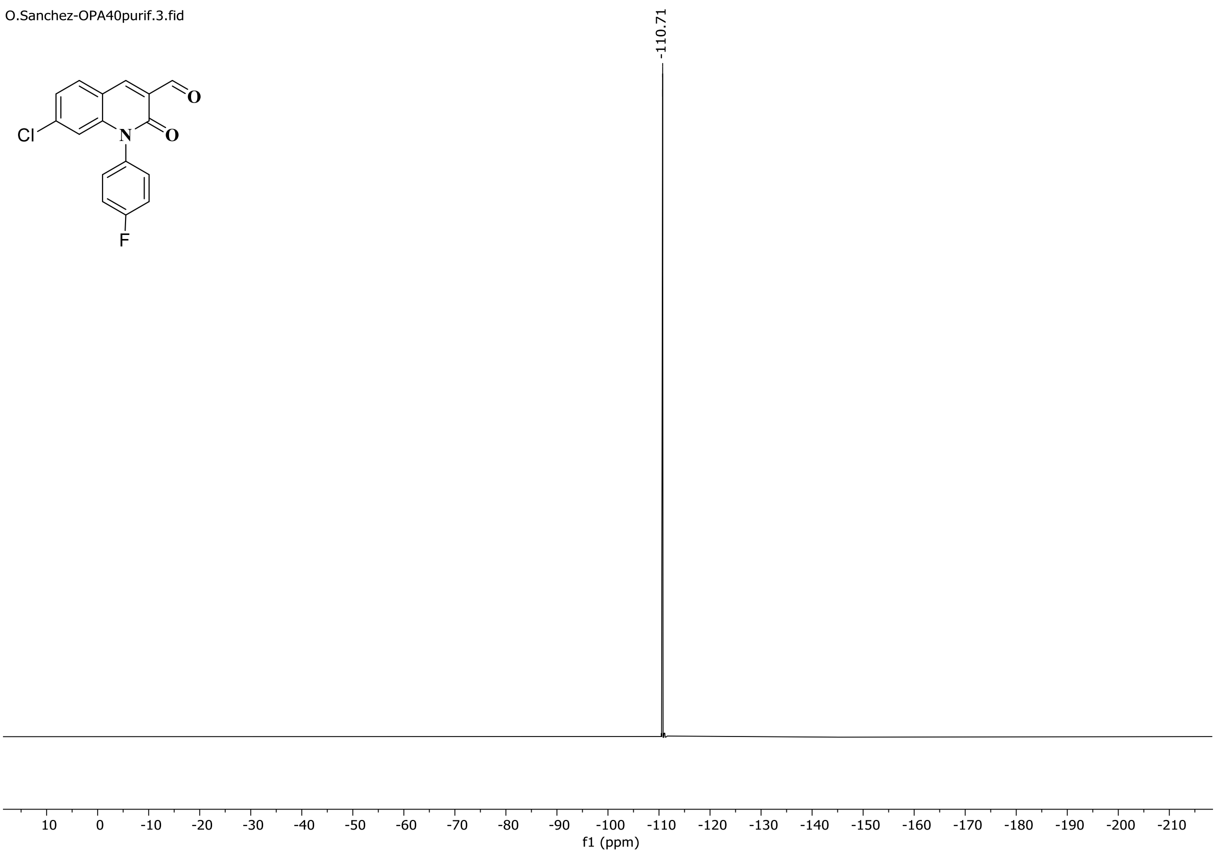

Figure S79. ^{19}F-NMR spectrum of **10h** ($CDCl_3$, 376 MHz)

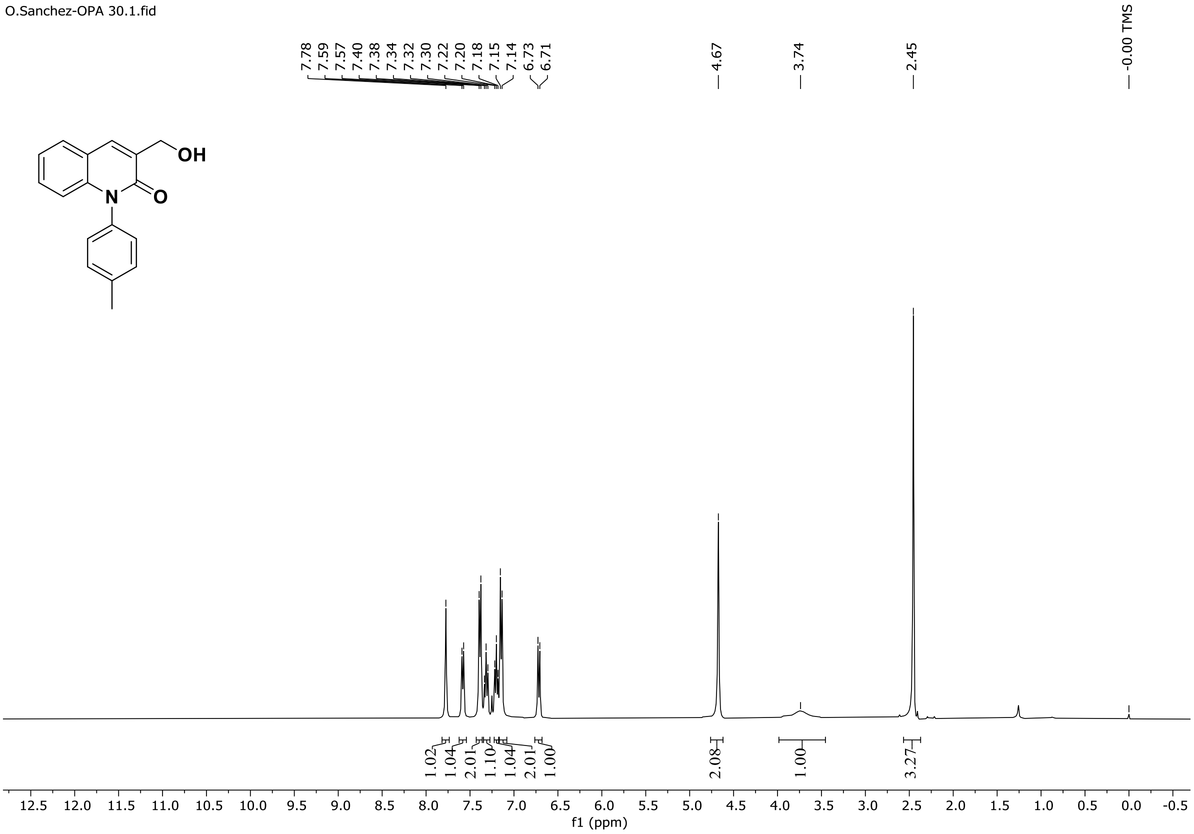

Figure S80. ^{1}H-NMR spectrum of **11** ($CDCl_3$, 400 MHz)

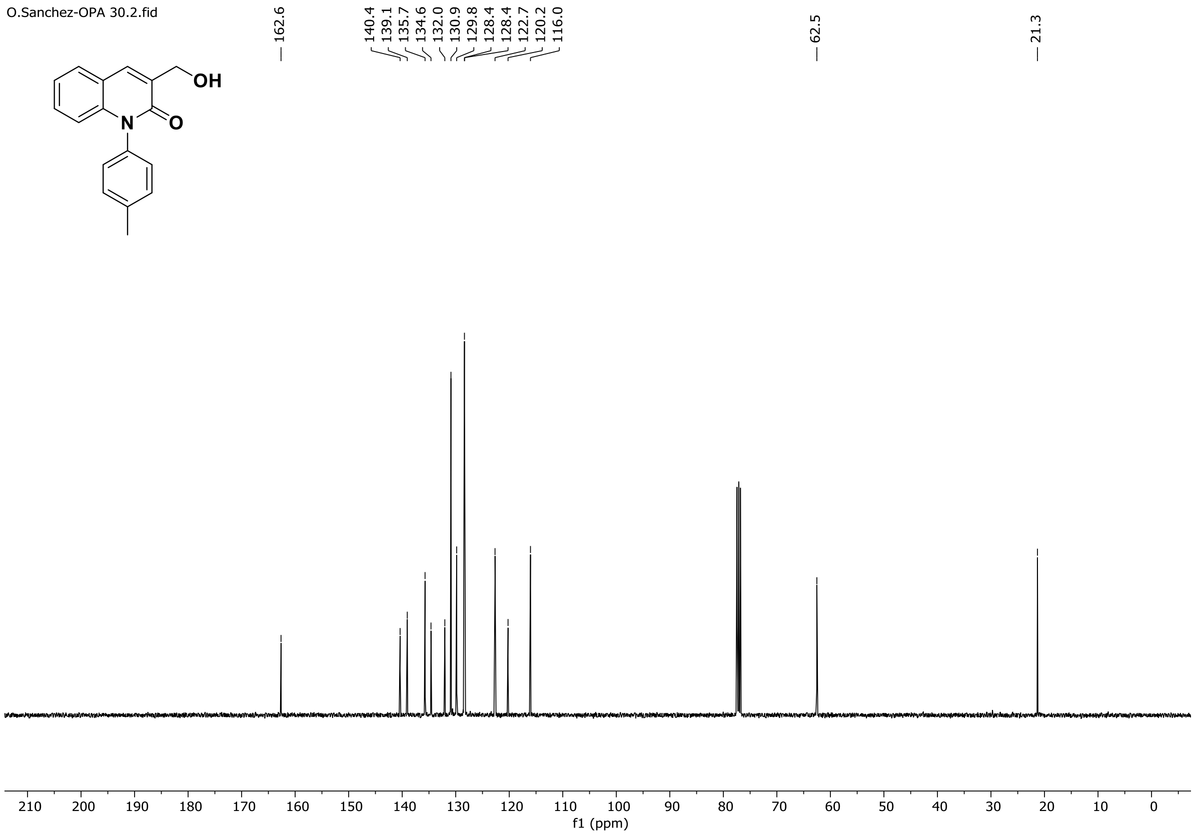

Figure S81. ^{13}C-NMR spectrum of **11** ($CDCl_3$, 101 MHz)

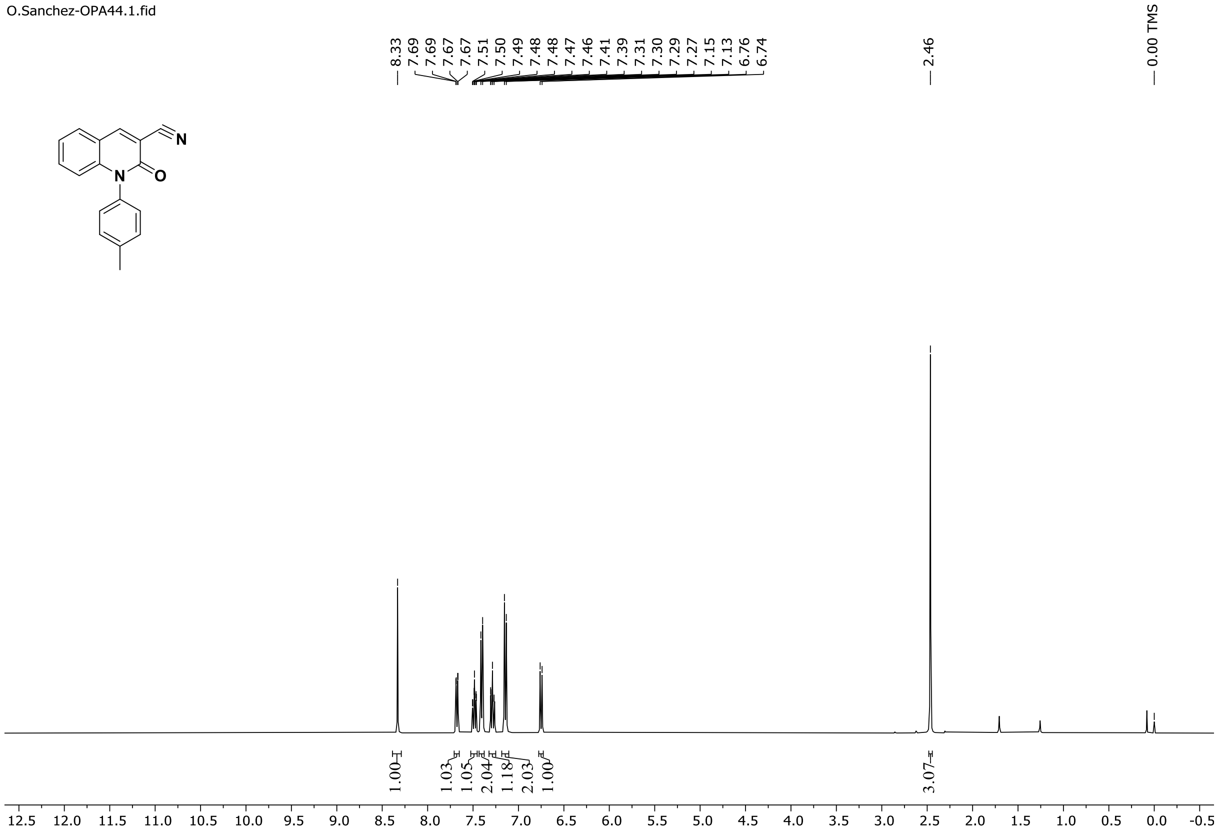

Figure S82. ^{1}H-NMR spectrum of **12** ($CDCl_3$, 400 MHz)

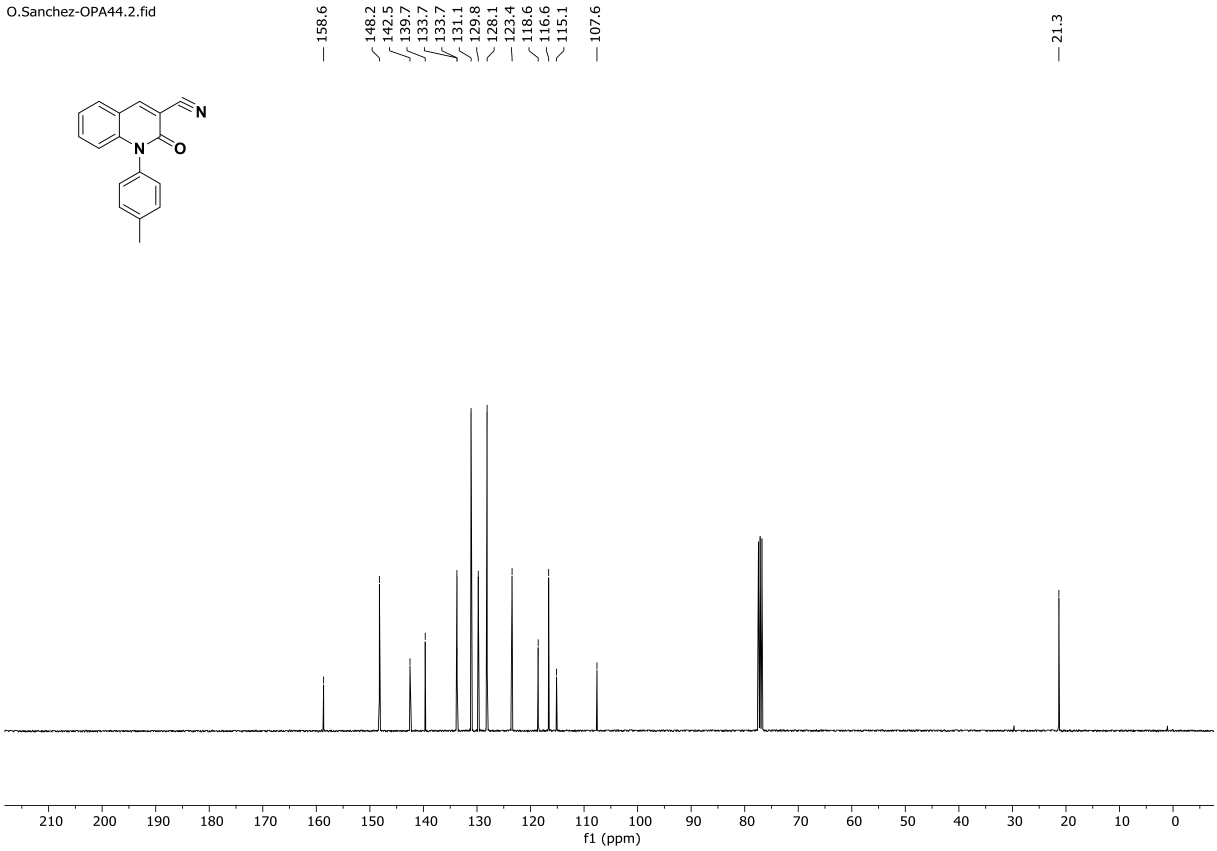

Figure S83. ^{13}C-NMR spectrum of **12** ($CDCl_3$, 101 MHz)

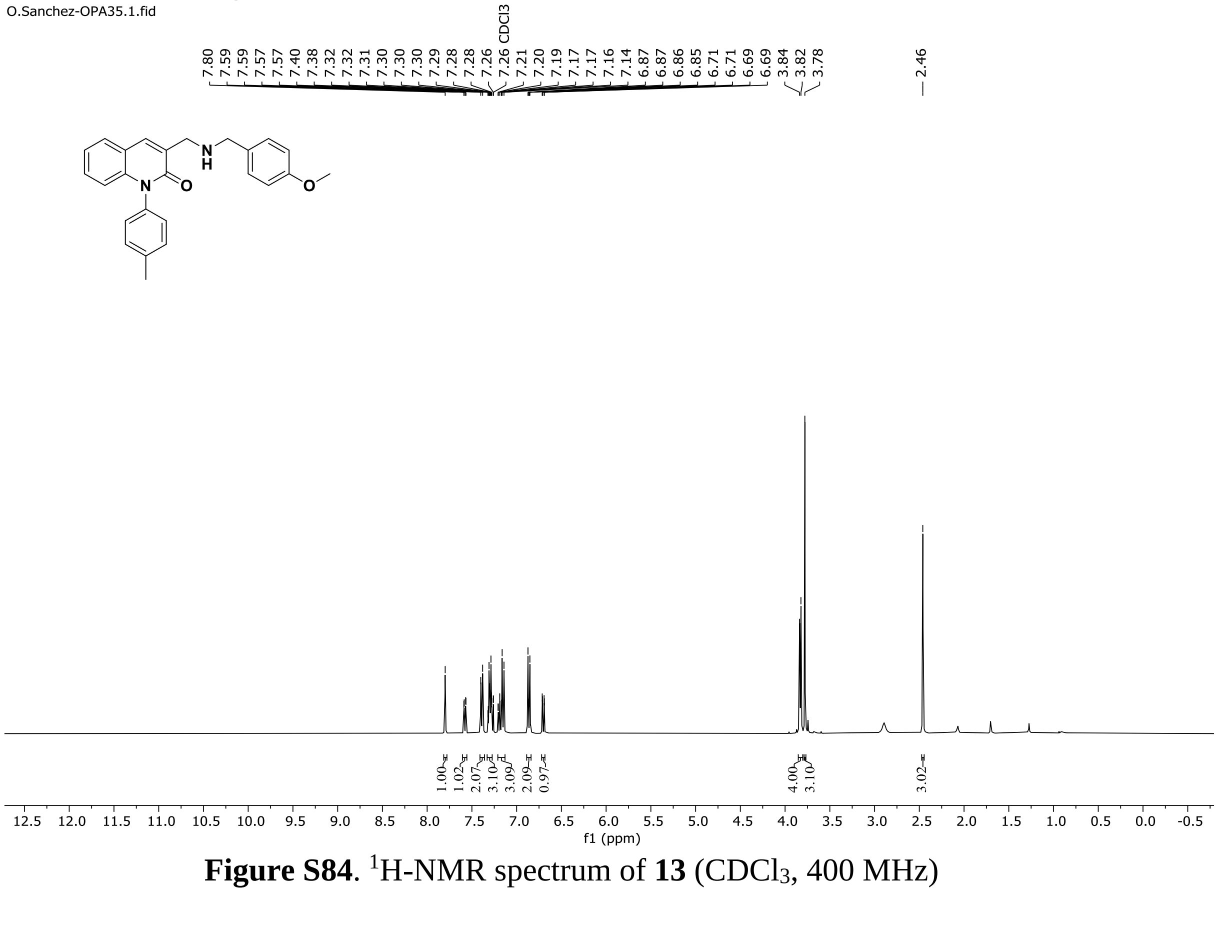

Figure S84. ^{1}H-NMR spectrum of **13** ($CDCl_3$, 400 MHz)

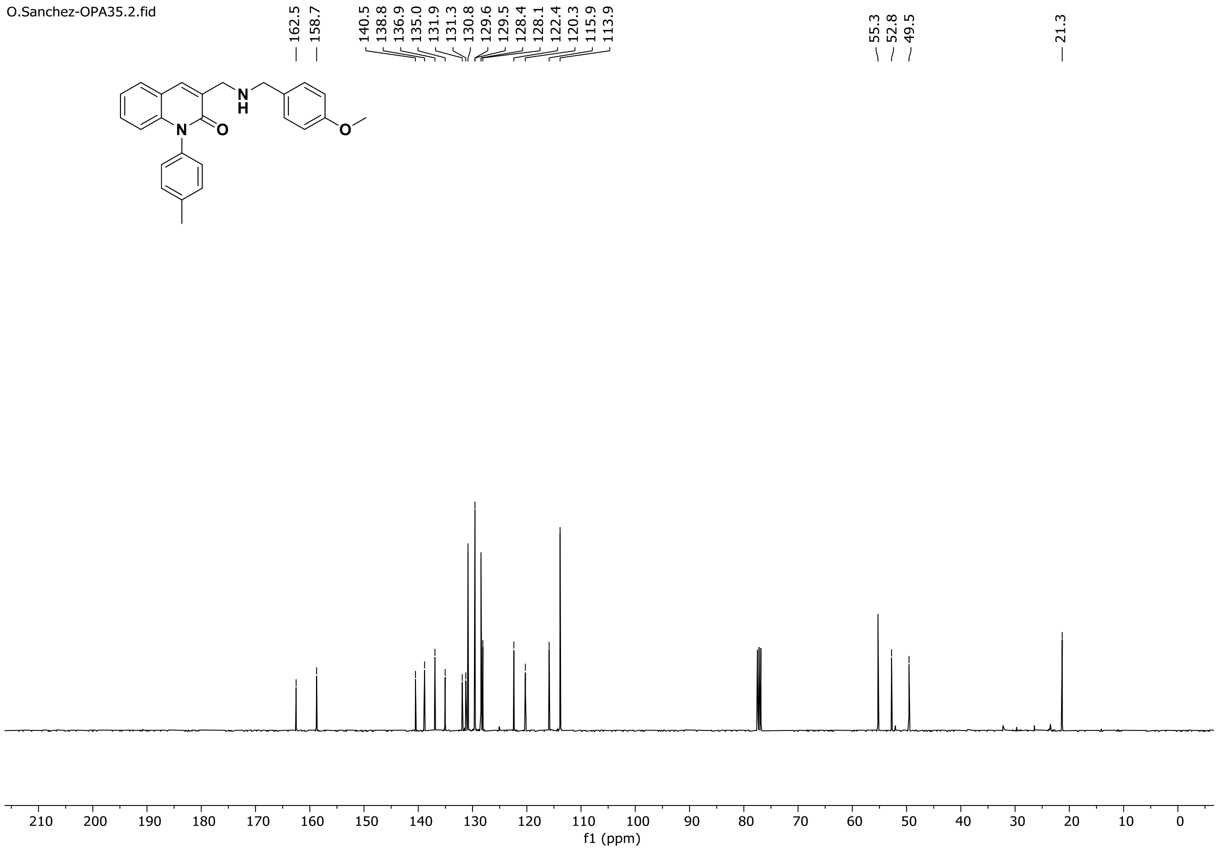

Figure S85. ^{13}C-NMR spectrum of **13** ($CDCl_3$, 101 MHz)

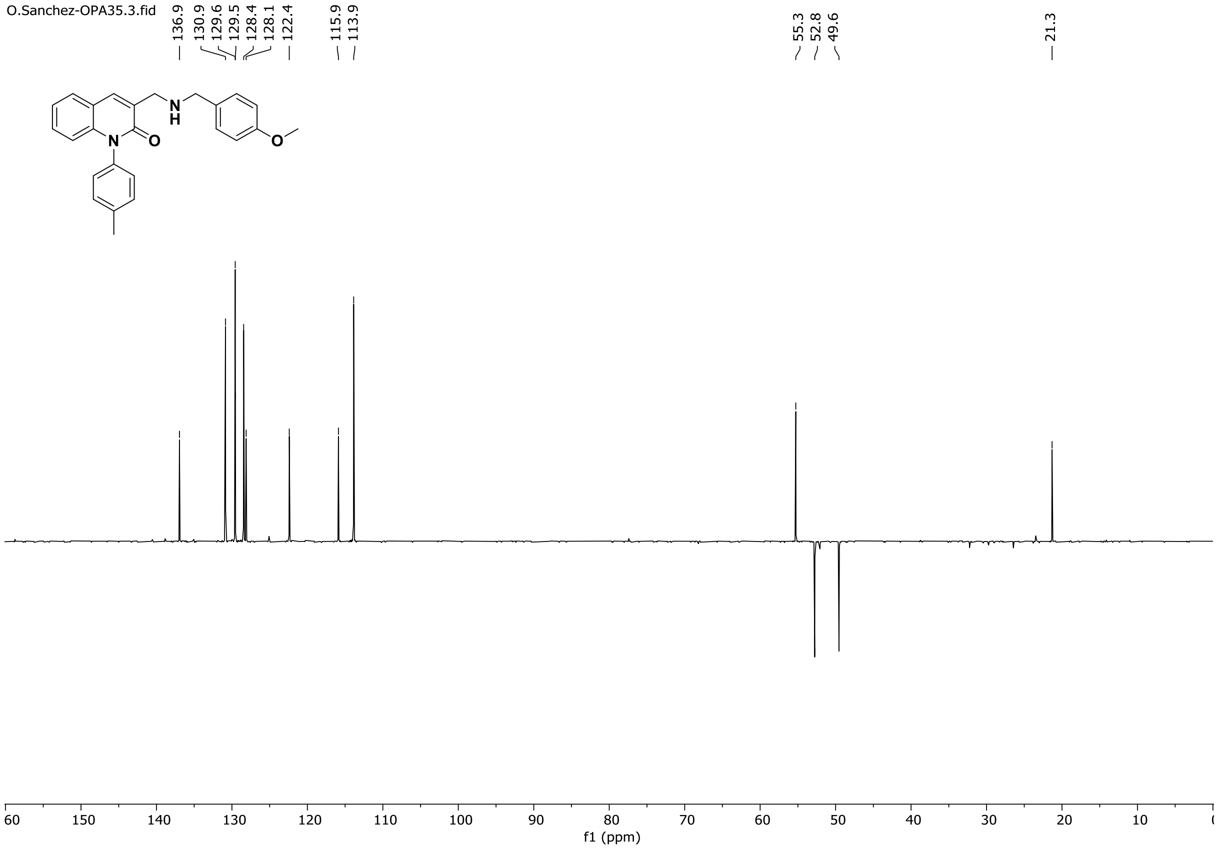

Figure S86. DEPT spectrum of **13** ($CDCl_3$, 101 MHz)

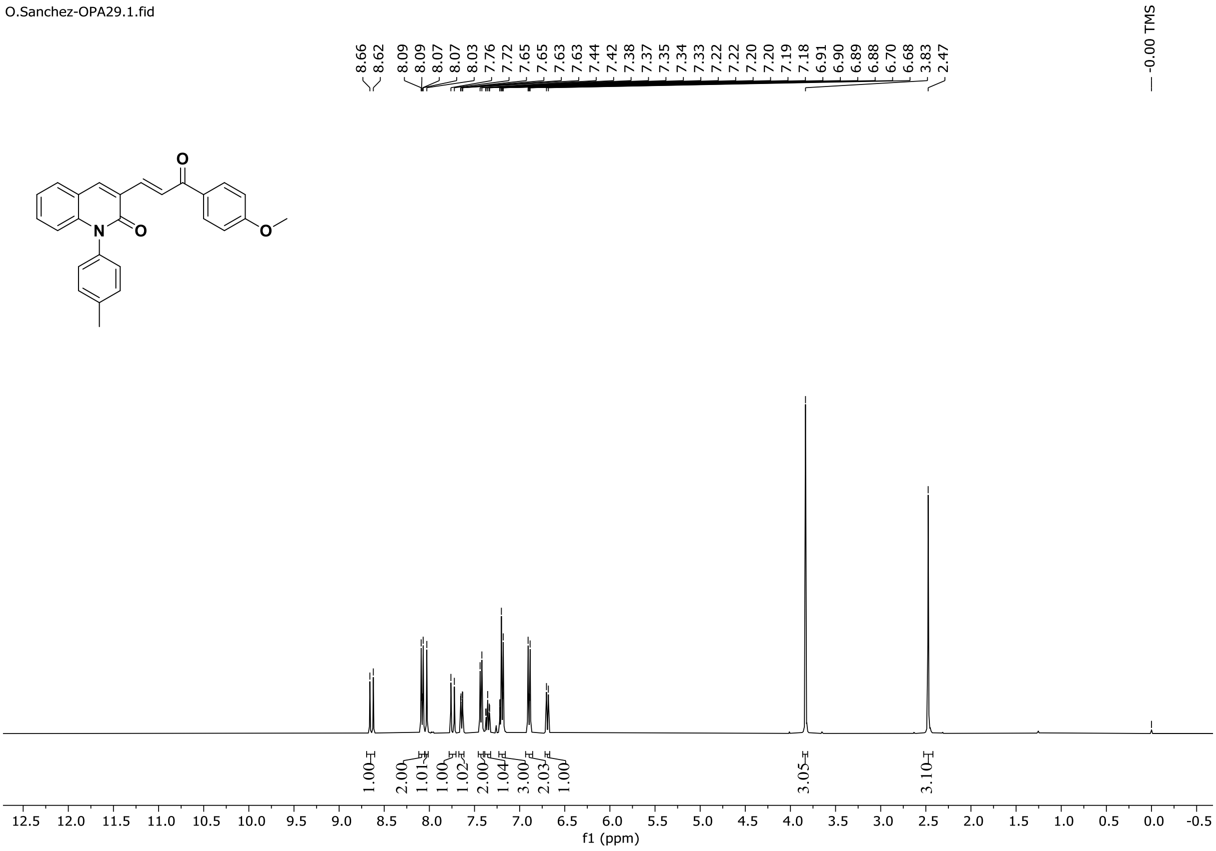

Figure S87. ^{1}H-NMR spectrum of **14** ($CDCl_3$, 400 MHz)

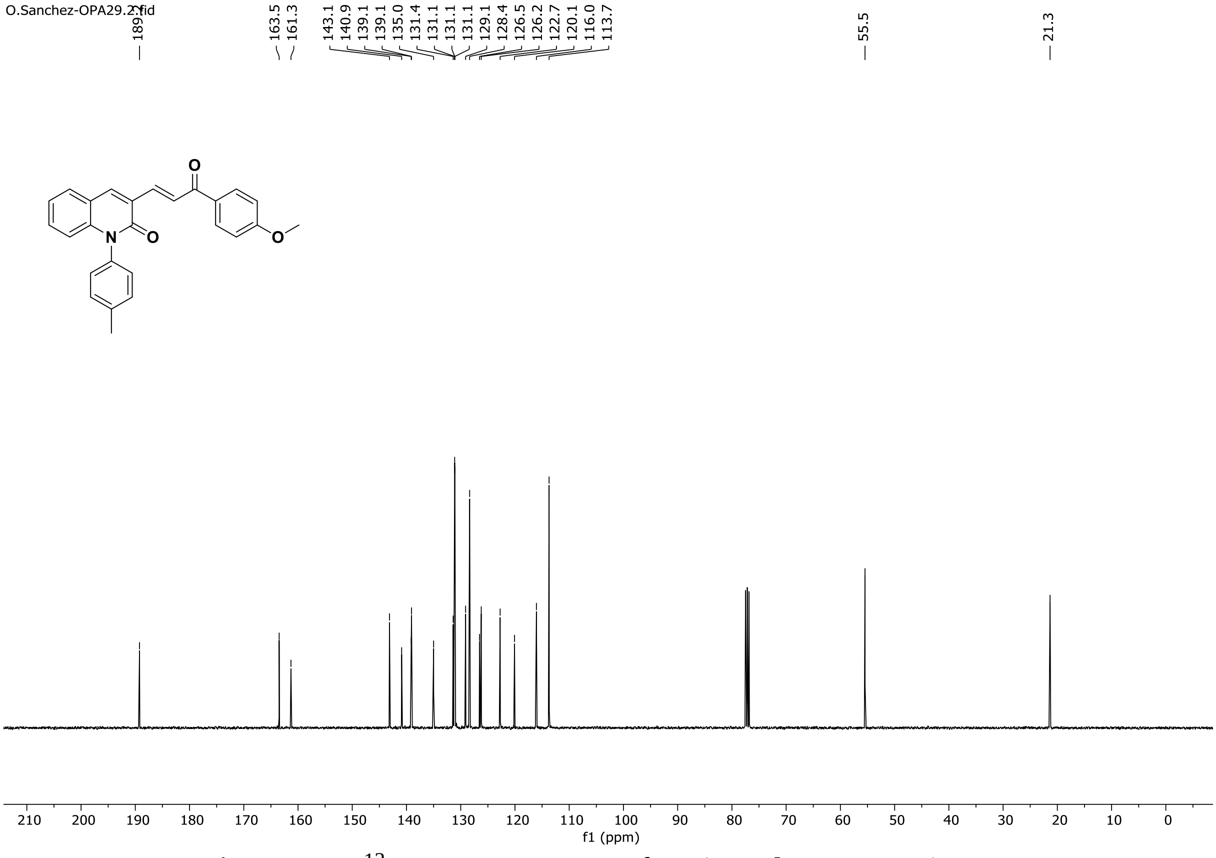

Figure S88. ^{13}C-NMR spectrum of **14** ($CDCl_3$, 101 MHz)

4-Unsuccessful reactions

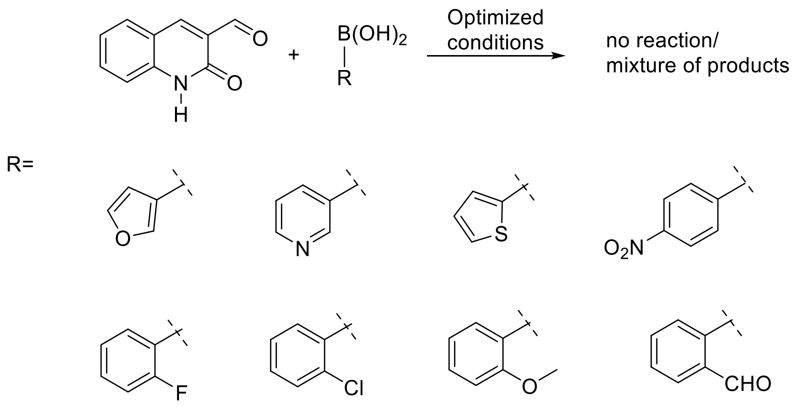

Supplement: Supplementary file 1 [file molecules-27-08345-s001.zip › molecules-1979457-supplementary.pdf]
